# Exploring physiotherapists’ knowledge and perception of exercise intensity in outpatient stroke rehabilitation: A qualitative study

**DOI:** 10.1371/journal.pone.0325098

**Published:** 2025-06-11

**Authors:** Félix Nindorera, Bénédicte Jean-Michel, Noémie C. Duclos, Krista L. Best, Stéphane Mandigout, Stéphanie Goncalves

**Affiliations:** 1 Center for Interdisciplinary Research in Rehabilitation and Social Integration (Cirris), Centre intégré universitaire de santé et de services sociaux de la Capitale-Nationale (CIUSSS-CN), Quebec City, Canada; 2 School of Rehabilitation Sciences, Faculty of Medicine, Université Laval, Quebec City, Canada; 3 HAVAE UR 20217, University of Limoges, F-87000 Limoges, France; 4 Université of Bordeaux, INSERM, BPH, U1219, Bordeaux, France; University of Pretoria, SOUTH AFRICA

## Abstract

**Objectives:**

To explore physiotherapists’ knowledge and perception of exercise intensity and to identify factors influencing the attainment of recommended exercise intensity during rehabilitation sessions.

**Methods:**

A qualitative study was conducted using semi-structured, face-to-face interviews with physiotherapists (PTs) who provide stroke rehabilitation within outpatient private settings. Data were audio-recorded, transcribed, and analysed using an inductive content analysis approach.

**Results:**

Twelve physiotherapists (five women; median age: 44.5 years; median experience: 20 years) who routinely provide stroke rehabilitation participated in the study. Three overarching themes emerged: knowledge about exercise intensity, perception of provided exercise intensity and factors influencing the achievement of recommended intensity. Although 92% of PTs recognized the importance of exercise intensity, only 33% were aware of exercise intensity guidelines. A lack of knowledge regarding exercise intensity assessment, grading, and monitoring was commonly reported. Nearly half (41%) of the PTs reported providing moderate-to-vigorous intensity exercise to stroke survivors. Limiting factors to achieving recommended exercise intensity included patient-related factors (e.g., stroke severity, motivation, cognitive impairments, fatigue), work environment constraints (e.g., limited space), and PT-related challenges (e.g., lack of knowledge, limited contact with doctors). On the other hand, facilitators included high motivation among stroke survivors, large workspace, equipment availability, and the use of group-based training modalities.

**Conclusion:**

Achieving the recommended exercise intensity in outpatient stroke rehabilitation is influenced by personal, environmental, and PT-related barriers. Multifactorial facilitators, such as improved knowledge, enhanced workspaces, interdisciplinary collaboration, and group-based training approaches, should be considered by stakeholders to ensure the effective implementation of evidence-based stroke rehabilitation practices in private settings. Future research involving PTs with diverse educational backgrounds are needed to strengthen the transferability of these findings.

## Introduction

Stroke is the leading cause of long-term disability worldwide [1], with disability prevalence remaining high over 3 years after stroke [2,3]. More than 60% of stroke survivors experience long-term impairments that interfere with activities of daily living (ADL) [4]. Moreover, mobility limitations observed in the year after stroke [5] can lead to sedentary lifestyles [6] which is associated with an increased risk of cardiovascular disease and recurrent stroke [7]. Exercise during physiotherapy is recognised as an important component of rehabilitation for secondary stroke prevention and enhancement of post-stroke outcomes [8]. Applying the FITT principle (frequency, intensity, time and type of exercise) during stroke rehabilitation is recommended to enhance motor and functional recovery and progress [9]. Exercise intensity, defined as a measure of the effort level or challenge placed on the body during physical activity, is commonly measured through an individual’s maximum capacity, such as heart rate (%HR max), %VO2 max, or % 1RM (One-repetition Maximum for resistance training). Intensity is a key factor in designing exercise programs to achieve specific fitness goals, with varying levels typically categorized as low, moderate, or vigorous [10].

Although participation in formal rehabilitation has been shown to reduce disability after stroke, accessibility and usability remain limited [11]. In developed countries, the attendance rate is around 30%, with fewer than one-third of stroke survivors benefiting from outpatient physiotherapy services in the USA [12] and 38.8% in France [13]. Private practices account for the majority of outpatient rehabilitation [14]. Furthermore, since private outpatient practices are self-managed and self-financed, organizational procedures such as performance monitoring, training priorities, quality and quantity of human resources, standardization of protocols, and leadership could negatively influence the implementation of exercise at recommended intensity [14–16]. Understanding how exercise is implemented into outpatient private rehabilitation settings, and how recommended exercise intensity is achieved, may help enhance accessibility and quality care.

The current stroke guidelines from the American Heart and Stroke Association recommend 20–60 minutes of moderate-to-vigorous intensity exercise (55% to 80% of maximum heart rate) 3–5 times per week and resistance training (50% to 80% of maximum resistance, 1RM) 2–3 times per week [17]. However, a limited number of stroke survivors achieve the recommended exercise guidelines, even during rehabilitation sessions [18–20]. For example, a recent systematic review revealed that the exercise intensity achieved during rehabilitation sessions was insufficient to induce a cardiopulmonary training effect post-stroke, with 21–80% of the sessions spent being inactive [21]. Furthermore, only 2% of 155 Canadian physical therapists reported using an exercise test that could be used to plan an appropriate exercise prescription program [22].

The most commonly documented barriers to adhering to recommended exercise intensity during rehabilitation include personal factors (such as stroke-related impairments, fatigue, embarrassment, fear of falling, and fear of recurrent stroke), environmental factors (like access, transportation, costs, equipment, time constraints, and staff availability), lack of knowledge, and social factors (such as lack of support) [23–29]. A recent scoping review highlighted that PTs’ knowledge and skills may contribute to the lack of emphasis on aerobic exercise during rehabilitation [30]. However, the four quantitative studies included in this review primarily focused on PTs working in inpatient or outpatient dedicated rehabilitation settings [30]. There is a lack of holistic qualitative research examining the factors that influence healthcare professionals, particularly PTs in outpatient private settings, in promoting and achieving the recommended exercise intensity during physical therapy with chronic stroke survivors. This study aimed to (1) explore PTs’ knowledge and perceptions regarding the exercise intensity provided to stroke survivors during rehabilitation sessions and (2) identify factors that affect the achievement of recommended exercise intensity in outpatient rehabilitation settings.

## Materials and methods

### Design

This is a descriptive qualitative study using semi-structured face-to-face interviews. It is part of a larger study (AVC-Lib) [31], which aimed to assess the physical activity levels of individuals with chronic stroke, both during outpatient physiotherapy sessions and at home. This study follows the Standards for reporting qualitative research (SRQR) guidelines [32]. PTs were initially contacted and informed about the study’s objectives. Verbal consent was obtained repeatedly before each interview, which was recorded and securely stored as both audio and transcript in the laboratory archives. The study was approved by the Limoges hospital ethics committee (CHU Limoges 35-2023-05) on July 10, 2023, and interviews commenced immediately thereafter. Interviews closed August 28, 2023.

### Participants

Fifty PTs working across 29 outpatient rehabilitation settings (private practices in the New Aquitaine region, France) were contacted via email by the study investigator (SG). All PTs were involved in the care of stroke survivors who had participated in previous studies [31,33]. Those who responded were screened based on the following inclusion criteria: 18 years of age or older, certified PT, working in outpatient private practice, and providing care for at least one chronic stroke survivor. PTs who expressed interest and met the inclusion criteria were invited to participate until data saturation was reached [34,35].

### Data collection

A public health student (BJM) with qualitative research experience collected sociodemographic information (age, gender, years of practice, type of additional training) and training modality (time session, frequency, schedule…), and conducted the interviews. All interviews took place in-person, in a calm and quiet room and lasted maximum 30 minutes. No previous contact or relationship existed between the interviewer (BJM) and the participants. The interview guide (S1 file) was developed by the authors (SG, SM) based on previous relevant literature [9,30] and was pilot tested with two PTs with 20 years of experience. The interview guide included questions about exercise intensity, knowledge of intensity guidelines, evaluation, training and monitoring of exercise intensity, perceptions of delivered exercise intensity, and factors (barriers and facilitators) influencing exercise intensity. Data collection was overseen by a senior trainee (SG) who maintained a detailed audit trail of data collection, enhancing dependability.

### Data analysis

Descriptive statistics were used to summarize participant characteristics. Qualitative interviews were transcribed verbatim using word-processing software and then analysed through an inductive content analysis process [36,37]. Participant statements were synthetized and adapted according to the conceptual domains described by Michie and colleagues, which served as the guiding conceptual framework [38]. The first author (FN) familiarized himself with the transcripts by reading them thoroughly before beginning coding, and re-reading transcripts throughout the coding and analysis. Transcripts were coded line-by-line using NVIVO 14 [39], and similarities between the codes and the interview guide were explored to generate main themes, sub-themes and categories [40]. A reflexive approach was used to enhance confirmability, such that FN critically examined biases, assumptions, and experiences as a PT to understand how this may influence interpretation of data. He also discussed interpretation of the finding frequently with KB and SG. To enhance credibility, codes and themes were discussed and reviewed among the research team, which comprised clinicians and rehabilitation scientists from two countries and was led by KB who is a professor and kinesiologist with experience in qualitative research. Findings were also discussed with colleagues who have expertise in stroke rehabilitation to gain additional perspectives. Themes, sub-themes and categories were named based on consensus. Finally supporting exemplars were selected to illustrate the themes and sub-themes.

## Results

Twelve outpatient PTs (5 women), median age 44.5 (range 28–66) years participated in this study between June and August 2022. PTs’ experience ranged from 7 to 41 years, with a median of 20 years. Three PTs (25%) had undergone complementary neurorehabilitation training. Regarding the training modalities provided to stroke survivors, the mean session duration was 60 (range 30–90) minutes, mostly 2–3 times per week (n = 6, 50%) on a regular schedule (n = 12, 100%). The training modality (individual or group) was equally performed among PTs (50% of PTs). Participant characteristics and sessions modalities are presented in Table 1.

**Table 1:** Sample characteristics.

ID	Age (yrs)	Gender	Experience (yrs)	Complementary training	Session duration (min)	Frequency of session per week	Training modality
**P1**	52	M	29	Neurorehabilitation	60	1–2	Individual
**P2**	45	M	22	Neurorehabilitation	90	2–3	Group
**P3**	48	W	27	No	90	1–2	Individual
**P4**	30	M	8	Other training	45	2–3	Group
**P5**	47	M	20	Other training	60	1–2	Group
**P6**	28	M	8	No	45	2–3	Group
**P7**	44	W	20	Neurorehabilitation	90	2–3	Group
**P8**	66	M	41	No	45	2–3	Individual
**P9**	41	M	19	Other training	30	2–3	Individual
**P10**	46	W	24	No	60	1–2	Individual
**P11**	34	W	12	Other training	45	1–2	Individual
**P12**	38	W	7	Other training	60	3 and more	Group

P=Physiotherapist; M=Man; W=Woman; yrs=years; min=minutes

**Fig 1:**
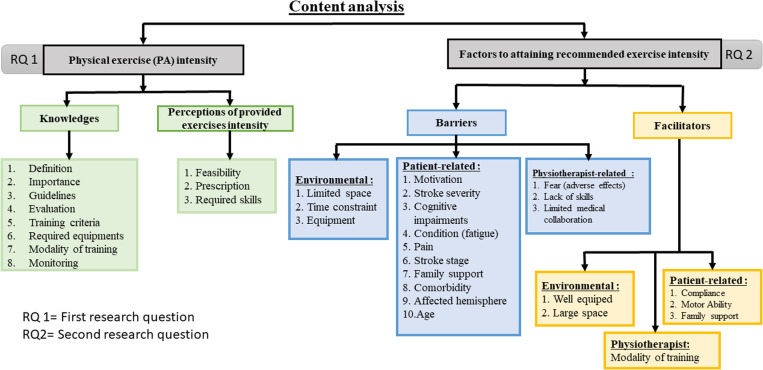
Overview of themes, sub-themes and categories. The first research question addresses information about exercise intensity (RQ1) and covers theme 1 (*knowledge of exercise intensity recommendations*) and theme 2 (*perception of applied exercise intensity during stroke rehabilitation*). The second research question (RQ2) covers facilitators and barriers to the implementation of exercise intensity and is summarized in a single theme (*theme 3*) as factor to attaining recommended exercise intensity.

Themes emphasizing knowledge to exercise intensity included the following sub-themes: definition, importance, and knowledge of exercise intensity guidelines, evaluation, training criteria, required training equipment, modality of training and intensity monitoring. Perception of provided exercise intensity included sub-themes of feasibility, prescription, implementation of exercise intensity, and required skills (training). The factors contributing to exercise intensity attainment comprised 2 sub-themes: barriers and facilitators to exercise intensity. The full overview of themes and sub-themes is presented in Fig 1.

### Theme 1: Knowledge

#### Definition.

The definition of exercise intensity was highly variable, including elements related to perceived fatigue (n = 4), exercise variations (n = 2), energy expenditure or heart rate variation (n = 2), exercise load (n = 1), and the number of exercise repetitions (n = 2). For example, a male PT (29 y experience, neurorehabilitation training; **P1**) defined intensity as, “*to train, for example on a stationary bike at 80% of the maximum load during an effort……...for example training at 70% of maximum resistance*…”

A 46-year-old woman (24 y experience, no neurorehabilitation training; **P10**) said: *[intensity] a more intense cardiovascular effort! …. getting out of your wheelchair and moving around… exercises shouldn’t last too long either, because in terms of muscular fatigue, they [*stroke survivors*] can quickly reach their limit. So, I leave them about the same number of repetitions on the muscle strengthening exercise, balance exercise and I try to change exercises about every 5, 10 minutes”*

A 38-year-old woman (7 y experience, no neurorehabilitation training; **P12**) defined exercise intensity as *“it’s a session where the patient will be put under sufficient strain, and we’ll see him/her more or less out of breath, or the heart rate will increase significantly*.”

The youngest among the PTs interviewed (28 years old, 8 y experience, no neurorehabilitation training, **P6**) reported that: [intensity]…*I ask them to take breaks, and they do movements after. The youngest patient who is 50 years old, performs 10 repetitions…and I think it’s enough*”.

#### Importance of intensity and knowledge of exercise intensity guidelines.

Although most surveyed PTs considered exercise intensity to be very important (n = 11, 92%), only four (33%) PTs reported being aware of guidelines. It should be noted that three of the four PTs completed neurorehabilitation training and worked in dedicated neurorehabilitation centres. *«That’s it, yeah, I think it [intensity] is pretty important for their independence, their muscular recovery, their cardiorespiratory recovery... [guidelines] no, not especially, I don’t quite feel up to it.” (***P6***, a 28-year-old PT with 8 years of experience but without neurorehabilitation or other training).* **P10** (46-year-old woman, 24 y experience, no neurorehabilitation training) reported: “*The way I look at [intensity], … it is important to give intensity so that patients can cope with a little more sustained effort*….[*exercise guidelines] Bah…… I had to redefine achievable objectives for the patient at each session, so that they wouldn’t find themselves in a situation of failure*”. Another 44-year-old PT (20 y experience and neurorehabilitation training reported that intensity is very important and highly recommended: “…*it’s very important because intense exercise is what’s effective, which means that patients who do intensive sessions progress faster than patients who do mobilization, so it’s... for the stroke population, I would say 180 minutes of moderate physical activity per week*.” (**P7**). P1 and P2 (45 and 52 years old, with over 20 years’ experience, neurorehabilitation training) said intensity is important to enhance everyday life activities: *“…[intensity] Ben... it’s important to cope with a more sustained effort in everyday life. So, innh, yeah, I know some HAS recommendations, but I don’t have much time for scientific reading”* said **P1**

#### Criteria for screening stroke survivors prior to exercise training.

PTs reported screening stroke survivors based on their age (young people are preferred to adhere to recommended exercise intensity), stroke severity (high level of physical and motor capacity preferred), and stroke stage (chronic stage preferred to adhere to recommended exercise intensity). Spasticity, multimorbidity, cognitive impairments, medication, as well as feelings and low motivation were considered limiting factors influencing adherence to recommended exercise intensity.

PTs with additional neurorehabilitation training support more intensive sessions, depending on the stroke survivor’s age and physical abilities. A 52-year-old PT (**P1,** 29 y experience) pointed out that young people easily adhere to recommended exercise intensity: “*Of course, there is the age criterion. but anyone can participate what counts is the evaluation.”* **P2** (45-year-old, 22 y experience) said he considers abilities when carrying out exercise sessions: “*Ah yeah, according to their capacity… So, during the medical history, we systematically ask people whether they have any cardio-respiratory problems, and we also ask them to bring in their complementary examinations.*”

Nevertheless, other PTs with no additional neurorehabilitation training were reluctant to push stroke survivors into moderate-to-vigorous intensity due to their restricted mobility, poor physical condition and low motivation. For example, a 47-year-old man (**P5**, 20 y experience) expressed “*I don’t do all the same exercises to all the patients, we adapt according to what they can do, depending on the mobility restrictions they have”*. On the other hand, they increase intensity in stroke survivors with good fitness and those in a chronic phase. A 46-year-old woman (24 y experience; **P10**) said: “*Yes, it’s for patients with much greater motor ability…...After 6 months post-stroke, I think you can increase the exercise intensity a little more*”. **P8**, the oldest of the respondent (41 y experience) expressed that, in addition to the person’s general condition, psychological criteria such as motivation are more important: “…*Because it’s not just physiological criteria that count [to adhere to moderate-to-vigorous intensity physical activity], there are psychological and motivational criteria that count too.”*

#### Assessment of exercise intensity, training criteria and monitoring of exercise intensity.

Three PTs (25%) claimed to perform objective assessments (e.g., submaximal tests, such as a 2 or 6 min walk test, or strength tests, such as sit-to-stand test) to determine the best exercise training zone, while 65% report using subjective assessments (stroke survivor’s state of health, pain, stroke severity, mood and motivation). None of the PTs reported performing or using an exercise stress test.

**P1** (Man, 52-year-old, 29 y experience, neurorehabilitation training) highlighted the importance of an exercise test to determine the training zone. He reported using heart rate and perceived exertion to monitor exercise intensity “*So, I have an exercise check-up, a customized health check-up. I do it in two ways; on treadmill or an overground test, or a resistance test…I train mostly in forms of 10 maximal resistance using a dynamometer or based on 1/10 patient’s weight…. It is always interesting to have a stress test, but it is not usually the case, it would be good to get it, as now we work based on maximal heart rate and age…. “*

It seems likely that PTs with no additional neurorehabilitation training preferred subjective versus objective exercise tests. For example, a 30-year-old PT (8 y experience; **P4)** explained that he did not required tests for assessment or monitoring exercise intensity; “*No specific test, most of my patients can’t manage a 6-minute walk test. We work on patient’s feeling to intensify exercise*”. **P11** who is 34 years old with 12 years’ experience follows the same approach as **P4**, reporting no need for a tool or test to monitor intensity during sessions: “*With a tool? No, I mean, I assess it [intensity] subjectively. I see if they are tired at the end of session…to find out if my patient is feeling well, I begin with 5 minutes exercise, then 10 and try to increase progressively…” “I am obviously not assessing it, I don’t have the time. Honestly, I don’t have much time…”*

Few participants (25%) agreed on objective criteria for grading and monitoring exercise intensity, such as resistance, training time or heart rate monitoring, while many PTs (66%), especially those without additional neurorehabilitation training, preferred perceptions of stroke survivors on fatigue, shortness of breath, or feeling. Perceived exertion was a commonly used method of monitoring exercise. Other indicators used, especially by PTs with additional experience included heart rate, blood oxygen saturation, blood pressure and clinical symptoms such as chest indrawing, breathlessness, dyspnea, sweating and skin coloration. The reported monitoring equipment were heart rate monitors, pulse oximeters and stopwatches. The number of repetitions and variation of exercises were the most widely used intensity grading methods.

**P1**, a 46-year-old man with 29 years’ experience, and **P3** a 48-year-old woman with 27 years’ experience, all reported that it’s important to use objective tools for exercise monitoring. **P1** said: “*So, I use the Borg scale most often, heart rate monitors sometimes for heart rate, I look at breathlessness, also a stopwatch of course is sometimes necessary*.” **P3** stated: “*We have an oxygen monitor, …. We also have a blood pressure monitor. So, we check blood oxygen saturation, heart rate and blood pressure”.*

#### Modality of training and equipment for exercise training.

The two types of training modalities (individual or group) were equally used, and the preference for group-based sessions was influenced by time efficiencies and motivation of the stroke survivor. Aerobic training equipment were the most used, while resistance training equipment were less common. Six PTs (50%) had at least one ergometer or treadmill in their office and six PTs (50%) had strengthening equipment (in addition to aerobic training equipment).

### Theme 2: Perceptions of provided exercise intensity during stroke rehabilitation

#### Feasibility of implementing exercise at recommended intensity.

Five (41%) PTs reported integrating moderate-to-vigorous intensity physical exercise during outpatient sessions. “*It [intensity] was moderate, I would say moderate …” said* **P1** (man, 52 y-old with neurorehabilitation training)*.* **P10** (46-year-old, woman, 24 y experience) reported*: “… the session I did with him wasn’t intense. it was a little adapted, may be low to moderate, depending on the patient’s fatigue, condition such as infection, any kind of pain, this can hamper the session.”* **P5** (47 years-old, man, 20 y experience) said *““with every patient, there’s obviously physical activity of moderate-to-vigorous intensity….”*

#### Prescription.

According to most PTs (75%), it’s the physician’s responsibility to specify the restrictions to exercise when prescribing rehabilitation sessions, but it’s up to the PT to set the training intensity. Exercise should be specified as a full detailed treatment on the prescription (minimum required intensity, frequency…). **P4** (man, 30-year-old, 8 y experience) said, “*When a medical doctor refers a patient to me, I expect he’s taken every precaution beforehand to make sure there’s no risk, and no, it’s not necessarily up to the physiotherapist to take that initiative”.* **P5** (man, 47 years old, 20 y experience) replied: “*Yes, it’s up to us [to calibrate intensity]. The doctor prescribes rehabilitation sessions and then we adapt the session as we see best!”*

#### Required skills.

All the respondents said it was important to have additional training in exercise intensity management. Only three PTs (25%) claim to have sufficient skills. One of the three PTs with additional neurorehabilitation training said “*Additional training is required. This was the case for me, because it’s something I was interested in, so additional training was already a basic requirement*.”

“*So skilled? I’m not sure, we can always do more… knowledge is required, the intensity level of patient sessions was not covered in our basic courses, obviously not*.” replied **P11**. Another 30-year-old PT with 8 years’ experience (**P4**) echoed the previous advice, saying: “*Yes, finally. I miss It [additional training], yes, finally. It needs to be integrated a little more into initial training*”.

### Theme 3: Factors influencing attainment of recommended exercise intensity

#### Sub-theme 1: Barriers to exercise intensity.

Challenges reported in ensuring exercise intensity during rehabilitation were related to the stroke survivor, work environment and PT.

PTs expressed barriers related to the stroke survivor as follows: lack of motivation (response from 83% of surveyed PTs), severity of stroke (83%), cognitive impairment (67%), fatigue or limited physical condition (58%), subacute stroke stage (33%), presence of pain (25%), co-morbidities (1%), isolation or lack of family support (1%), and affected hemisphere (1%).

#### Lack of motivation.

Regardless of experience (varying from less than 10 years to more than 20 years) or additional training, PTs reported lack of motivation among stroke survivors as a major barrier to achieving the recommended exercise intensity: “*Ah, it’s the motivation, the lack of motivation sometimes in some patients that makes it difficult for them to participate*”, argued **P1** (52-year-old, 29 y experience, neurorehabilitation training)”. **P6** (28 years-old, 8 y experience, no additional training) said: *“Sometimes the patient gets discouraged too....yeah, discouragement on the part of the patient, because of too much routine”.*

#### Stroke severity and affected hemisphere.

A 46-year-old PT said *«The stroke severity, level of disability? Oh yes, of course, this is a big obstacle*” **(P10)**. *“When patients don’t see any motor recovery and they still have a high level of disability, they give up*” said **P11** (34-year-old, 12 y experience). **P1**, a PT with 29 years’ experience and neurorehabilitation training, added: *“it’s a left hemiplegia Hein, it’s still too severe, and most of the time with cognitive problems”.* Similar arguments emerged from three PTs from 8 to 27 years of experience. “*Well, it depends on a lot of things, especially the stroke severity. It depends on the recovery*” **(P3); “***Mobility restrictions, so people who can’t stand up, no physical exercise in fact”* **(P4);** *“The level of disability too. I think that the more severe the person’s hemiplegia, the more complicated it is to go for higher intensity exercise”* **(P6)**.

#### Cognitive impairments.

Cognitive impairments have been identified as a limiting factor to understand exercise instructions. For example, 3 PTs, including one with additional training in neurorehabilitation, all reported the impact of cognitive impairment. A 30-year-old said: “*…as well, eh! cognitive limitations can be a hindrance to rehabilitation if the instructions are not properly understood or if the patient is in constant danger*.” A 52-year-old with 29 years of experience with additional neurorehabilitation training highlighted: *«Cognitive impairment is a major limitation”*; and a 46-year-old with 24 years’ experience completed: “… *patients with cognitive deficits are difficult to manage, no intensity*”

#### Fatigue and/or limited fitness.

Fatigue was identified as an important barrier to training intensity*.* **P11** (34-year-old PT, 12 y experience, no additional neurorehabilitation training declared; *“It’s more likely that patients will get tired very quickly, that’s for sure. Fatigue happens much more quickly compared to other patients”.* **P2** also said that stroke survivors are too fatigued, which prevents them from pushing the intensity too far: *“Too much fatigue is a limit”*.

#### Stroke stage.

Some PTs considered that exercise in the acute and sub-acute phases may represents a great risk for stroke survivors, and therefore preferred to engage moderate-to-vigorous intensity exercise during the chronic stage*.* **P7** (aged 45, 20 y experience and neurorehabilitation training) reported *“No, it’s not the same importance. Because for me, it’s more important in the chronic stage, because there’s the secondary prevention aspect, whereas in the acute or sub-acute stage, you still waiting for recovery,...and intensity adaptation is a risk”*

Other barriers raised by PTs were pain “*Left hemiplegia often leads to neurological pain which can be disabling and compromise intensity work”* said P1; the presence of co-morbidities “*If he has any cardiac problems, I don’t do a lot of exercise’* **(P5)**; older age *“Because, on the other hand, the man who’s 80 years old, I’m not going to do any strengthening for him other than the weight of his body*” **(P8);** and social isolation *“Sometimes it’s people who live alone, so when they don’t have a social support, they’re not stimulated”* **(P11)**.

Reported barriers linked to the work environment (outpatient private services) included the limited space (66% of PTs), the session time constraint (16%), and the lack of or high cost of training equipment (treadmill, bike, 2%). “*Having an adapted space that allows you to... to accommodate more severely disabled patients is a challenge*” (**P1)**; “*there must be a technical facility with equipment that is both accessible and safe*” (**P2**); “ *equipment. We really need it*” (**P5**)

Regarding barriers related to PTs, the lack of sufficient knowledge about exercise intensity, the fear of exercise training adverse effects, and limited contact with the medical doctor were the predominant barriers.

#### Fear of harmful effects.

A 47-year-old man with 20 years of experience reported: “*Barriers include the fear of* [moderate-to-vigorous intensity]*. Fear of hurting patients. Well, the fear of doing too much, of forcing the patient too hard, and then either having a heart attack or something else, for example, or intensifying the pain*”. **P11** (34-year-old, 7 y experience, no additional training): “*We avoid* [increasing exercise intensity] *for fear of something else happening because they’ve almost all had cardiac problems…. I wouldn’t put them in any difficulty about that*”

#### Lack of knowledge.

Lack of knowledge about PA guidelines and exercise intensity was reported as a major barrier by 42% of the respondents. A 66-year-old physio with 41 years’ experience said “…*I didn’t do any additional training related to physical exercise in stroke rehabilitation. So, my knowledge dated back to 1983. They didn’t tell us anything about intensity”.* A young 30-year-old PT (8 y experience) stated: *“yes, I need a refresher course, at last. I need it, yes, finally. It [additional training] needs to be integrated a little more into initial training*”. Finally, a 44-year-old PT reported that intense exercise was avoided to stroke people with spasticity: “*No, in my initial training, we were told that above all higher intensity exercise increased spasticity [exercise considered a contraindication], that it was very dangerous and that above all they shouldn’t get tired, that’s it. No, I didn’t do any specific training on the intensity of the sessions”*

#### Limited contact with the medical doctor.

Some PTs (16%) reported that it is difficult to have ongoing follow-up contact with referring physicians. **P5** said “*yes, we’re afraid of forcing the patient too much because he could get sick, for example pain could increase...as it’s not easy to get in touch with his doctor for an opinion”*.

#### Sub-theme 2: Facilitators to exercise intensity.

The most reported facilitators to implementing exercise at recommended intensity during outpatient private rehabilitation settings were large workspace, stroke survivor’s motivation, peer support, modality of training (group-based sessions) and continued professional education, such as additional neurorehabilitation training. “*in fact, to have an adapted space that allows you to feel at ease in your workspace, to be able to welcome patients who are more disabled than a sprained ankle, or whatever*; replied ***P1;*** patient’s motivation ***“****I’d say that a patient who’s motivated, who has the desire. It’s true that we’ll be able to push him to do more intense things,* said **P6***”;* and peer support & group-based sessions *“I think that the fact that patients are not alone is good for them to have other people around to talk to, to exchange ideas and to see that others are also working,* **P3***”*

## Discussion

This study aimed to investigate physiotherapists’ knowledge and perceptions regarding the exercise intensity prescribed to chronic stroke survivors, as well as to identify the factors that hinder or facilitate adherence to moderate-to-vigorous intensity exercise sessions in outpatient private practice settings. To our knowledge, this is the first European study that examined the perspectives of physiotherapists working in private outpatient settings with chronic stroke survivors. The private practice environment differs significantly from hospital and specialized care settings in terms of organizational structure, care management, coordination, and financial aspects [14]. The findings of this study revealed a lack of knowledge and awareness among physiotherapists regarding exercise intensity guidelines. Furthermore, fewer than half of physiotherapists reported delivering exercise at the recommended intensity. Several barriers and facilitators were identified that affect the implementation of these guidelines.

### Knowledge and perceived provided exercise intensity

Although all participants believed that increasing exercise intensity is important and should be incorporated into sessions for chronic stroke survivors, most lacked knowledge about physical exercise guidelines and were not aware of criteria for exercise implementation, screening, grading, and intensity monitoring. Furthermore, 41% of respondents (mostly PTs with additional neurorehabilitation training and working in dedicated practices) reported providing moderate-to-vigorous intensity to stroke survivors during their sessions. The limited knowledge about exercise intensity among PTs may be attributed to the prioritization of training courses by each facility, which is primarily influenced by the characteristics of the population served and the associated costs of training programs. Moreover, the limited implementation of exercise at recommended intensity in outpatient private practice may be attributable to mainly the French health system organization where only medical doctors can prescribe rehabilitation sessions, without, maybe stressing the importance of grading exercise intensity.

These findings confirm the existing discrepancy in the prescription and implementation of exercise between dedicated stroke units (specialized neurorehabilitation clinics) and private outpatient practices. In Canada and the USA, more than 60% of PTs working in dedicated facilities (neurorehabilitation clinics) prescribe and implement aerobic exercise for stroke survivors at the recommended intensity [41,42]. A recent systematic review on the implementation of clinical guidelines for stroke rehabilitation among healthcare professionals identified knowledge and skill gaps as the most frequently cited barriers to guideline adherence [43]. The review also highlighted a lack of collaboration, characterized by limited professional interactions, and insufficient organizational processes as key factors negatively influencing guideline implementation [43]. It is important to note that private practices are self-managed and self-financed, which may pose significant barriers to multidisciplinary collaboration, continuing education, standardized care, quality management, and funding.

### Challenges to integrating recommended exercise intensity in outpatient private practice

The primary barriers to implementing moderate-to-vigorous intensity exercise in private practice were associated with both stroke survivors and PTs.

The most frequently reported barriers related to the stroke survivor included lack of motivation and stroke severity (83%), cognitive impairments (67%), and fatigue (58%). These findings align with previous studies indicating that the most commonly perceived barriers are related to the individual’s health status, including concerns about safety and ability, cognitive limitations, fatigue, and emotional well-being [30,44]. Furthermore, physical symptoms has been identified as key barriers to adherence to physical activity among older adults with chronic conditions [45]. A systematic review by Moncion et al. showed that higher-functioning participants were more likely to be prioritized for aerobic physical exercise, while over 75% of rehabilitation programs excluded stroke survivors with severe impairments [30]. Our findings also identified novel barriers related to the stroke survivor, such as lack of social support, older age, and stroke stage (specifically the subacute phase). In outpatient private settings, stroke survivors are responsible for managing their own transportation and scheduling appointments. The lack of social support, including encouragement and accountability, combined with logistical challenges such as self-management of transportation and scheduling, can result in frustration, decreased motivation, and reduced adherence to exercise programs.

Limited knowledge and awareness of exercise intensity prescription was reported as the most common PT-related barrier, regardless of age or years of experience. This barrier may stem from concerns about potential adverse effects, particularly in the absence of immediate access to medical doctors—unlike hospital settings or specialized centers, which benefit from a multidisciplinary team. However, PTs with neurorehabilitation training did not report this barrier and expressed no apprehension about increasing exercise intensity during sessions. The issue of insufficient knowledge was also highlighted in Moncion’s scoping review, which concluded that access to and continued education in structured aerobic exercise programs could enhance adherence to recommended guidelines [30]. Additionally, limited interprofessional interactions and collaborations, along with the absence of organizational processes, may further hinder the effective implementation of guidelines [30]. Regarding the work environment, most PTs identified restricted workspace as the primary barrier, rather than session time or equipment availability. Consistent with previous studies, barriers such as lack of equipment, limited space, inadequate administrative support, and time constraints are frequently encountered in the implementation of evidence-based practice in stroke rehabilitation and across various physical therapy settings [30,43,46]. Although time limitations have been widely recognized in the literature as a significant challenge [43,47], it was not a predominant concern in our sample. This may be attributed to the training modality, as many physiotherapists in the present study conducted group sessions, which served as an effective time-management strategy.

### Facilitators to integrating moderate-to-vigorous intensity exercise

We identified several facilitators for achieving the recommended exercise intensity during outpatient stroke rehabilitation, which were categorized into three domains: work environment, stroke-survivor factors, and PT-related factors. Receiving continuing education on physical activity and exercise intensity in neurological conditions, as well as integrating physical activity courses into the core curriculum, emerged as key facilitators for implementation. Similar observations have been reported in the literature, indicating that PTs working in stroke and other rehabilitation settings express a strong interest in acquiring exercise-related knowledge and enhancing their physical activity skills [48,49].

**Clinical/practice implications**: Improving PTs’ knowledge through continuing education and fostering group-based sessions can enhance recommended exercise intensity adherence for chronic stroke survivors. Addressing patient motivation and environmental constraints is essential for better rehabilitation outcomes.

**Implications for future research**: Further studies should involve PTs with diverse educational backgrounds to enhance the transferability of findings and inform targeted interventions.

### Limitations

This study has several limitations. First, all participating PTs were trained under the previous educational system, which awarded a bachelor’s degree, whereas the current French curriculum requires a master’s degree to qualify as PT. Consequently, the findings may not be fully transferable to the entire population of PTs. Further research involving PTs with diverse educational backgrounds is needed to improve the transferability of findings. Nevertheless, the inclusion of 12 PTs spanning different generations and levels of experience, all working in multisite outpatient settings, strengthens the study’s representativeness.

Second, credibility of the findings may be limited due to the absence of triangulation or member checking. However, data collection was conducted by a non-clinician professional with no prior relationship with the participants, which may be considered a strength, as it likely reduced potential biases and ensured that participants responses were neither influenced nor directed during the interviews.

## Conclusion

This study provides insight into the current PT practices in private rehabilitation settings, a sector often overlooked by public health services. PTs in private practice have limited knowledge of exercise intensity guidelines and do not consistently reach the recommended exercise intensity for chronic stroke survivors. The barriers identified were primarily related to individual factors (e.g., motivation, stroke severity, lack of support), the environment (e.g., restricted space), and PTs (e.g., limited knowledge, limited interaction with medical doctors). Raised facilitators for achieving exercise intensity recommendations included continuing education, group-based sessions, high motivation, and peer support. Current findings highlight factors that stakeholders can address to improve the quality of outpatient private rehabilitation care.

## Supporting information

S1 file Interview guide. Interview guide with the formulation of key questions. This guide has been validated by the team and tested before starting data collection.
(PDF)

S2 file Data transcript in their original language.
(PDF)
