# Supplementary material for: Exploring physiotherapists’ knowledge and perception of exercise intensity in outpatient stroke rehabilitation: A qualitative study
Source: PLoS One. 2025 Jun 11;20(6):e0325098. doi: 10.1371/journal.pone.0325098 (PMC12157079; doi:10.1371/journal.pone.0325098)
Supplement: S1 file — Interview guide. Interview guide with the formulation of key questions. This guide has been validated by the team and tested before starting data collection. (PDF) [file pone.0325098.s001.pdf]

Interview guide

1. Introduction

Good morning.

I sincerely thank you for agreeing to participate in this interview and for devoting your time to me. My name is ……………………………….a Master degree Public Health student at the University of Limoges.

I am interested in the intensity of rehabilitation sessions for post-stroke patients in the chronic phase. The interest of my work is to explore the experience of physiotherapists on this intensity of sessions in order to identify the facilitating factors and barriers to its implementation. There is no right or wrong answer. What interests me is your experience.

In order to process the data from the interview, I would need to record it. This recording will only be useful to me for written transcription purposes. All the information extracted will of course be anonymized before transcription and the data will only be used for this end-of-study work. They will then be destroyed. Do you allow me to record?

Presentation of the interviewee

To begin this interview, can I please ask you to introduce yourself quite briefly: your age, the date of your diploma, your professional experience and your additional training?

How long have you been practicing as a freelancer in an outpatient settings? Out of 100 patients, how many post-stroke patients do you have in your patient registry? How many times do you see them per week and how long does the session last? Is it in a group or individually? Is the number of sessions limited in time or do you see these patients continuously?

Part 1: Knowledge about intensity of exercise

Following the preliminary results of the observational study in which your stroke patients participated, we noticed that the sessions' intensity differed from one patient to another. How do you view this observation?

1. How important is exercise intensity to you for these patients and why?
2. Are you aware of any recommendations in terms of exercise intensity for this population or at all?

3. In your opinion, was the session we observed with your patient intense? no way ? slightly intense, moderately intense, vigorously intense? Justify why

Intensity of sessions

4. **Question:** Is there a type of patient that you give strenuous exercise to?
5. **Reminder** : *What criteria do you use to select these patients?*
6. **Question:** do you attach the same importance to the intensity of the session in the chronic phase as in the subacute phase after stroke? For what ?
7. **Question :** Do you need the patient to have completed a stress test or the doctor to have given you permission to increase the intensity of the exercise?
   - ⇒ *Refresher: Do you have them do a submaximal exercise test like the 6-minute walk test or the chair rise test or something else?*
8. **Question :** Do you evaluate the intensity of the sessions in your patients? If yes, how ?
9. **Question** : What equipment do you use to increase the intensity of your sessions (ergometers, treadmill, etc.)?
10. **Question:** Do you adapt the intensity of the exercises according to the patient's physical condition? How do you adjust the sessions (duration, intensity, frequency)?
11. **Question:** What monitoring criteria do you use when you increase the intensity of the sessions (HR, saturation? Perception of effort)?

Part 2 : Barriers and facilitators

Environmental context and resources

1. **Question:** In your opinion, what equipment is necessary to achieve a certain level of intensity during the rehabilitation sessions of these patients?
   - ⇒ **Relaunch** : *What equipment do you need to increase the intensity of your sessions?*
2. **Question:** In your opinion, what are the barriers or obstacles in terms of equipment to its practice?
3. **Question** : Regarding the intensity of your sessions, is lack of space a limitation? ?
   - ⇒ **Reminder:** If so, what space would you need?
4. **Question** : Does the time you have in session seem sufficient to you?
   - ⇒ **RELAUNCH** : *If you find you don't have enough time, how much time do you need?*

<u>Patient and physiotherapists related-barriers</u>

1. **Question** : Do patients have any restrictions on doing more intense exercises? Which ?
2. **Question:** and you, do you have any obstacles to making these patients do more intense exercise?
3. **Relaunch** *: Could the severity of the disability be a barrier? could cognitive disorders be a hindrance? Could motivation be a barrier? Could fatigue be a hindrance? could pain be a hindrance? Could patient anxiety be a hindrance? Could the consequences that this could cause at the cardiac or other level be a hindrance?*

<u>Other questions.</u>

1. **Question:** It is recommended to do 20 minutes of moderate-intensity physical activity during a rehabilitation session for these patients. Do you think that we should integrate it into routine practice, into physiotherapy treatment or do you think that it should be the subject of complementary sessions such as adapted physical activity sessions?
2. **Question:** Do you prescribe exercises for the patient to do at home? If so, how often ? for what duration? and at what intensity?

Conclusion

This is the end of the interview. I thank you for this time spent with you. Would you like to add a final remark regarding the theme we discussed today? Or do you have any questions for me? I will now turn off the audio recording device.

We will come back to you with the results of this new study in which you have just participated. Thank you very much again!
