# Supplementary material for: Exploring physiotherapists’ knowledge and perception of exercise intensity in outpatient stroke rehabilitation: A qualitative study
Source: PLoS One. 2025 Jun 11;20(6):e0325098. doi: 10.1371/journal.pone.0325098 (PMC12157079; doi:10.1371/journal.pone.0325098)
Supplement: S2 file — Data transcript in their original language. (PDF) [file pone.0325098.s002.pdf]

I
Fichier audio
audio1915334272 2.m4a
<u>Transcription</u>
BJM
Donc pour débuter cet entretien, puis s'il vous plaît, vous demandez de vous présenter brièvement votre âge, date de diplôme, expérience professionnelle, formation complémentaire.
K1
Alors moi je suis kinésithérapeute depuis 1994. Euh, je suis en cabinet libéral, neurologie. Depuis janvier 2000. Donc ça fait un peu plus de 20 ans maintenant que le cabinet fonctionne. Et j'ai à peu près 90% de patients atteints de pathologie neurologique, alors qui ont principalement des scléroses en plaques. Ensuite en 2e, je pense que ce sont plutôt des parkinsoniens et ensuite en 3e, les AVC. Voilà, et mes séances mes ces séances durent 1h par patient d'échauffement sous forme de petits ateliers de travail. Et après une 2e partie de travail plutôt en duo avec le… le... le kiné. Sur des objectifs définis Il y a…récupération de l'effort de d'étirement, d'assouplissement ou de massage.
BJM
Les voyez-vous sur une durée ? Limitée des séances ou bien en continu ?
K1
Je les vois, non ? J'ai les patients que. Je suis depuis très longtemps maintenant, donc c'est on est beaucoup dans le chronique, je dis on parce que je ne suis pas tout seul à travailler dans ce. Domaine-là sur Bordeaux, je. Nous, c'est quand même beaucoup dans la chronicité. C'est-à-dire que j'en ai parfois qui viennent pour une demande En particulière
BJM
D'accord donc, à la suite des résultats de l'étude Observationnelle à laquelle vous avez participé, nous avons remarqué que l'intensité des séances différait de patients à l'autre, donc Quel regard portez-vous sur l'intensité des séances de kinésithérapie ?
K1
Le regard que j'ai sur le Ben je… je pense que c'est important. Je pense que c'est important de mettre de l'intensité. Je travaille beaucoup sous forme de de de de RM, la 10 RM. Et également donc je… je me base sur des sur des pour bilanter j'utilise des dynamomètres pour ou… ou bien le… le je mesure par le poids de la personne 1/10 du poids du corps de. La personne et cette personne-là doit faire le soulever un poids par exemple, 10*1/10 de son poids. Par exemple, pour une force de quadriceps ou une force de biceps. Donc, donc je vais utiliser soit des. Ou bien à l'aide d'un dynamomètre Pour les membres supérieurs.
BJM
D'accord, qu'entendez-vous par intensité des séances ?
K1
Ça, si j'ai répondu à la question.
BJM
Oui, oui. Qu'entendez-vous par intensité des séances chez les patients post AVC en phase chronique
K1
Eh bien, l'intensité, c'est de de travailler, par exemple sur un vélo d'appartement à 80% de la charge maximale lors… lors d'une épreuve d'effort par exemple sur le vélo à bras, c'est euh, euh, chez une hémiplégique Euh Sur une durée de 10 Min sur un avec un système de résistance sur Le vélo à bras. Euh donc voilà après c'est la musculation par exemple, je...je, je travaille à peu près à 70% de la force maximale, 60, 70%.

BJM
D'accord, quelle importance accordez-vous à l'intensité, dans la mise en place d'un vos. Séances. Et pourquoi ?
K1
Pourquoi je mets de l'intensité, c'est ça ?
BJM
Quelle est importance accordez-vous à l'intensité, dans la mise en place de vos séances ? Et pourquoi ?
K1
Ben je, c'est quasiment, je dirais. J'ai, je voilà, je. Je pense que je mets à peu près. 60% de. De de résistance de puissance et 40% de travail d'endurance aussi quand même. Et dans les 60% ? Pourquoi ? J'apporte de l'intensité, parce que je pense. Qu'il faut leur donner du. Une, une capacité à se. À à subvenir à un effort un peu plus soutenu. De la vie quotidienne donc j'essaie d'aller toujours un petit peu un un petit peu au-delà des gestes de la vie quotidienne, ce que j'ai. Ils sont tous sédentaires pour la plupart, donc j'essaie de justement de de les travailler, de de les mettre beaucoup au sol. Beaucoup de travail au sol, avec des des balances, des appuis sur des bases. En donc ce sont des gestes assez concrets, du des appuis, des tractions et parce que je pense oui. Pour répondre à la question, parce que je pense. Que ça, ça ? Répond un peu aux aux gestes de la vie quotidienne.
BJM
D'accord, connaissez-vous des recommandations en termes d'intensité d'exercice pour cette population ou pas du tout ?
K1
Euh bon je lis un petit peu mais j'ai pas beaucoup de temps de de lecture scientifique mais je fais une réunion une fois par mois avec des collègues et à cette occasion là je prends des informations, un. Petit peu peu. Par rapport à la HAS. Des références de de ce côté-là, mais j'ai pas beaucoup de temps pour de la lecture scientifique.
BJM
D'accord, à votre avis, la séance que nous avons observée avec votre patient était-elle, intense ou pas du tout ? Légèrement modérément intense et pourquoi ?
K1
Il était modéré, je dirais modéré celle que bon il y a. Il y a déjà un petit bout de temps mais je pense qu'elle a. Été modérée, c'était une patiente. C'est une patiente qui, qui, qui est coopérante hein, mais qui nécessitait quand même d'être qui nécessite d'être relancé, d'être. Donc je pense que c'était plutôt modéré je pense.
BJM
D'accord, pourquoi elle était ? L'intensité était modérée, pourquoi pas intense pour cette patiente ?
K1
Et parce que parce que c'est une une, une patiente, Ben je ne sais pas, on peut-on peut rentrer. Dans le détail. Ou pas ? Parce que bon.
K1
c'est une hémiplégie gauche Hein, donc hémiplégie gauche, c'est quand même. Cognitivement parlant, c'est un peu compliqué quoi donc ? Je pense que c'est +1 un problème cognitif. Je pense, je pense, je veux dire, on a. Un peu cette barrière là .
BJM
Selon vous, quel est l'environnement technique matériel ? Et professionnel.
K1
J'ai pas compris.

BJM
Selon vous, quel est l'environnement technique ? Matériel et professionnel nécessaire à la mise en œuvre d'intensification des séances de rééducation chez les patients post AVC?
K1
Je pense qu'il faut un espace. Je pense qu'il faut un espace adapté. Un espace sécurisant ? Et sécuriser. Sécurisant pour le le patient. C'est-à-dire un espace de mobilité. Composé d'espalier Qui se font face à face. Qui fait qui mesure un espace qui fait à peu près 2 M sur 2 M de large sur 1 M de long. Et dans lequel les patients, puissent se mouvoir en toute sécurité. Et je dis aussi sécurisé pour le pour le kiné, que le kiné. Soit pas toujours à côté de lui, de peur que le patient tombe par exemple. Donc on a essayé de concevoir un espace adapté pour ce type de patient dans lequel les les patients peuvent se rattraper à des à, des prises, des des espaliers. Un peu partout, il y avait dans le cabinet, j'ai des j'ai, des espaliers partout, j'ai des des prises murales à tous les dans tous les coins. Un peu dangereux donc je pense que je pense enfin personnellement qu'il faut un espace adapté pour ce type de patient. Surtout les plus lourds, bien sûr, mais. Parce qu'on travaille, je, je travaille pas, surtout à part les gens obèses, je travaille très. Peu sur un podium de sur une table de Booba par exemple, je travaille beaucoup au sol. Donc pour cela, il faut quand même un espace assez grand, quoi. Mais bon Ceci dit dans un ce que j'explique parce que j'accueille des étudiants, ce que j'explique aux étudiants c'est qu'on peut très bien concevoir un un espace dont dont je vous ai donné les mesures tout à l'heure dans un cabinet classique. Et dans lequel le patient est hémiplégique Pouvoir se se mettre dans cet espace qui est contenant, sécurisant.
BJM
D'accord
K1
Donc oui, il faut du matériel.
BJM
Par exemple quel matériel auriez-vous besoin ? Pour faire ce type de séance.
K1
Mais il faut 2, 4 espaliers qui se font côte-À-Côte. Mais après moi, je je vous invite à peut-être, si vous. Avez un moment. D'aller voir un blog, on a, on a créé un blog neuro kinésithérapie l'espace et la mesure ça s'appelle Neuro kinésithérapie. L'espace et la mesure dans lequel on décrit notre notre espace de travail. Donc c'est 2 espaliers. 4 espaliers Regardez, voilà 2 espaliers qui se font face à face. Comme ça ? Comme dans un rectangle en fait. Dessus, une échelle. Des des poutres. Pour que ça puisse qu'on puisse faire éventuellement des barres parallèles. Un espace ? Créant un. Part parallèle, la mesure s'appelle. D'accord, donc c'est c'est c'est des, c'est des espaliers, c'est des des. Et des espaces d'espalier.
BJM
Selon vous, quels sont les freins ou obstacles environnementaux à sa pratique ? Et quels sont les facteurs facilitants ?
K1
Alors, les obstacles, c'est un, c'est justement de pas avoir un cabinet adapté, donc d'avoir un cabinet qui qui ne puisse pas avoir de de. Sécurisant comme je viens de décrire jusqu'à la question précédente. Également l'obstacle, c'est. Dès qu'ils qui sont peut-être pas trop intéressés par la la chronicité parce que c'est des patients qui. Que l'on suit de longue date, qui ne guérissent pas de de leur hémiplégie, donc auquel il faut accompagner au cours au fur et à mesure de leur vie. Donc ça, ça peut rebuter un petit peu les certains kinés. Ben nous, c'est nous, c'est moi. C'est enfin, c'est moi, c'est, c'est Ce que j'aime justement, la chronicité, c'est Ce que j'aime chez ces patients là, j'aime bien accompagner les gens. Comme ça ? Et ça peut, ça peut être un obstacle pour d'autres kinés,

ouais. Et et quels sont les facteurs facilitants? Le côté Ben justement, c'est d'avoir un espace adapté qui permet de justement de d'être bien dans son espace de travail, de pouvoir accueillir des patients un peu plus lourds que une entorse de cheville, ou voilà.

BJM

D'accord, le temps disponible dans une seule séance de.

K1

Donc tout est dans l'espace en fait.

BJM

Le temps disponible dans une cellule en.

K1

Moi ça, moi, ça me convient à 1h.

BJM

1h.

K1

Moi, ça me convient. À 1h, 1h par ci, 1h par patient c'est très bien, ouais.

BJM

Donc comment sélectionnez-vous les patients à qui vous faites faire de l'exercice plus intense ? Sur quel critère vous vous basez par exemple ?

K1

Bon, il y a le critère de l'âge quand même. Un patient très âgé je vais peut-être moins moins lui faire travailler dans en en en résistance encore que. Si ça peut être le facteur un petit peu. Voilà bah patients très âgés, on. Voilà, ça peut être bon après ce qui peut être aussi bloquant Non, c'est les gens qui qui ont qui n'ont pas de possibilité de se mettre au sol. Là j'en ai une justement une patiente-là qui est très forte. Et je vois donc je la, je la, j'avais quand même travaillé en résistance mais. Je la mets sur le podium sur un plan de Bob Batt donc. À l'athée, hein, bien sûr, mais là, je suis-je suis un peu limité par le manque de motivation aussi de la patiente. Donc voilà les facteurs un peu limitants, quoi. Ça peut être un peu ça, j'en ai quelques-uns qui ne sont pas toujours à demander de l'effort, de l'effort et ceux-là Ben je je je m'adapte et je je mets plus de l'endurance que de que la que la résistance.

BJM

Avez-vous besoin que les patients aient effectué une épreuve d'effort ou bien que le médecin vous ait donné l'autorisation d'intensifier l'effort?

K1

Alors, c'est toujours intéressant d'avoir une épreuve effort, c'est, c'est rarement le cas, on l'a on, on l'a rarement, mais si on peut faire remonter cette étude sur une demande, enfin, moi personnellement je je pense que ce serait intéressant d'avoir parce que sinon on travaille un petit peu à l'aveugle, on fait un petit peu, 220 mois l'âge et puis après on travaille à 60, 70% du 220, moins l'âge. Mais bon moi, moi ayant travaillé longtemps, entraînement à l'effort chez les cardiaques, je sais que cette formule-là elle est, elle n'est pas. Elle n'est pas fiable parce qu'ils ont des médicaments, ils ont des qui ralentissent le rythme du cœur. Donc donc moi je suis très demandeur d'épreuves. Effort, ça, c'est. C'est ça, reste quand même des patients vasculaires, donc. Euh, ça s'est arrêté au niveau du cerveau, mais ça, ça aurait pu s'arrêter au niveau du cœur. Leur caillot ou leur infarctus quoi donc ? Donc oui, moi je suis très demandeur de d'épreuve d'effort, oui.

BJM

Et évaluez-vous l'intensité des séances chez vos patients ? comment paramétrez-vous ces séances ?

K1
Alors j'ai un bilan, un bilan, un bilan exercice. Euh personnalisé ? Qui utilise des des, des échelles validées bon le Barthel, le, le Passe, le Tinetti, le. L'EPA, l'EPD Mais j'ai aussi mon bilan auquel je compare. En fait, ce que je fais, c'est que je compare ces bilans validés. De mes bilans qui sont des bilans un peu empiriques, hein, que j'ai accumulés au fur et à mesure de mon expérience. Et donc je fais des je fais un bilan après sur Excel. Et après ? Sur Word pour. Renvoyer ça aux… aux médecins traitants. Lui, au… au neurologue. D'accord, donc oui, j'ai un bilan personnalisé pour tout, tout, chaque patient.
BJM
Quels outils d'entraînement utilisez-vous ? Comme par exemple un ergomètre, tapis roulant ? pied libre
K1
Alors j'ai un j'ai un vélo d'appartement, un vélo à bras, un vélo de rééducation à bras. Un banc de musculation. Un cyclorameur, tapis de course. Je l'ai dit, non ? Tapis de marche. Un banc de musculation. Je l'ai dit, un cyclo hélicoïde alors ça, je l'utilise, y a j'ai peu d'épique qui m'ont dessus. Mais bon je je le dis quand même que que j'ai ça. Des Steppers les Trampolines.
BJM
Adaptez-vous l'intensité des. Exercices en fonction de l'état physique du patient ou bien est-ce que c'est par exemple ce que c'est ? Le moment ? C'est quand le patient vient ? C'est en fonction de du patient ou. Bien en fonction du.
K1
Et en fait j'ai 2, j'ai 2 types de prise en charge, j'ai la prise en charge de bilan. Donc là où je fais. Mon protocole que je suis à la lettre ? Enfin, au fur et à mesure des séances qui me prend pas mal de temps, c'est un. Bilan qui me qui s'étale sur une vingtaine de séances, hein ? Et. Et à l'issue de ce bilan ? J'en conclus des 1BK, un bilan diagnostic, kinésithérapique explique et et j'adapte en fonction des objectifs des patients, des objectifs kinés. Et puis après, j'ai aussi l'autre un. Voilà, ça c'est les 2, les 2 volants. Et puis après à la demande c'est à dire si j'ai une demande particulière, patient qu'un. Problème de dos ou Bon Ben là je réponds à la demande à ce moment-là, mais j'ai, j'ai toujours ce. Ce protocole de bilan et ensuite de de d'objets de BDK.
BJM
Comment agissez-vous les séances en termes de durée, d'intensité et de fréquence ?
K1
Alors la la ? Je crois que je l'ai, je l'ai dit, en fait la. La séance se compose de 3 parties, une première partie où on fait un échauffement. Sous forme de petits ateliers qui ont pour qui ont pour mission de travailler, soit le la souplesse. La force ou l'équilibre ? Après la 2e partie, c'est le le, le le, le la séance individuelle avec le Kiné, donc en binôme, ce où là on travaille plus le, le bilan, le bilan ou le BDK. Les objectifs à atteindre. Et puis la 3e partie, c'est le temps de d'étirement, d'assouplissement. Ou de massage ?
BJM
Donc leur faîte Vous faire un test d'effort sous-maximal comme le test de marche de 6 Min.
K1
Oui. Alors je fais, je le fais de 2 manières. Je le fais. Pour le tapis de marche. Ou à l'extérieur ? Et à l'extérieur, pardon.
BJM
Donc, quels sont ? Les critères de surveillance utilisez-vous lorsque vous intensifiez les séances avec les patients post sa AVC en phase chronique.
K1

Alors je j'utilise un peu le, le l'échelle de Borg, donc l'échelle d'essoufflement que je mesure un petit peu. Donc y a voilà qui est assez intéressante. Après, quand je fais le. Le le le test de marche. Je suis avec eux. Et je teste pareil l'échelle de Borg, l'essoufflement. Euh qu'est-ce que et quels outils vous me disiez, c'est ça ?
BJM
Oui. Quel ? Quel critère par exemple, est-ce que vous utilisez dans certains cardiaque, la saturation, la perception d'effort ?
K1
Vous demandiez ? Alors là. La perception d'effort. Alors, la fréquence d'une épreuve d'effort sous la main. Sinon je fais, je le fais de manière certains patients, des des cardio fréquencemètre donc oui la. Fréquence cardiaque, je peux ? Je, je l'utilise des fois oui, tout à fait. Et l'échelle de Burg le l'essoufflement. Donc chronomètre bien sûr. Chronomètre, goniomètre.
BJM
Oui, vous pouvez continuer ?
K1
Chronomètre, goniomètre, chronomètre, goniomètre et un 1 M 1 M, ruban. 1 M Banc de ce type-là là. Pour mesurer la souplesse, et cetera donc. C'est les mètres rubans, c'est les. Voilà les maîtres, un ruban comme ça, j'ai toujours sur moi, on mesurait les les amplitudes articulaires. Je fais en mais je les mesures articulaires, je les fais en centimètres en fait. D'un d'un point osseux à un autre point osseux, et je fais rarement un degré. Je trouve qu'il a beaucoup d'erreurs de source d'erreur donc.
BJM
Selon vous, quelles sont les connaissances théoriques, techniques et pratiques dont doit disposer le kinésithérapeute pour intensifier les séances de rééducation ?
K1
J'ai pas compris le début de la phrase.
BJM
Quels sont les connaissances théoriques, techniques et pratiques dont doit disposer le kiné ? Pour faire ce genre d'exercice au patient post AVC?
K1
Ouais ça seulement, je pense que je pense que la formation initiale suffit. On peut faire un peu de de formation complémentaire. Moi, c'était mon cas parce que c'est quelque chose qui me m'intéressait, m'interpellait donc les. Les formations complémentaires mais déjà à la base. De la formation et déjà très bonne je pense, moi je. Ce que j'ai fait en plus, c'est. Oui, peut être les niveaux d'évolution motrice les NEM. Qui sont peut-être importants à revoir quand on sort de l'école, donc les NEM Donc un institut dans des instituts de formation, le le font ça, hein ? Et puis. Et puis, après avoir un intérêt. Une appétence dans cette dans cette dans ce domaine-là. Mais je pense que déjà la formation initiale, elle est elle est, elle est elle est suffisante hein ?
BJM
Donc à votre avis ? Qu'est-ce qui empêche une augmentation de l'intensité des séances de rééducation ?
K1
Les troubles, les troubles cognitifs. Et l'âge ? Alors moi je moi je mettrais, voilà, voilà les 2 barrières. Là je là, je suis patient et des troubles cognitifs.
BJM
Est-ce qu'il y a des freins concernant les patients à faire de l'exercice plus intenses ?
K1

Ah les freins, mais c'est la motivation, le manque de motivation parfois chez certains patients. La douleur ? La douleur pour les hémiplégies gauches ? Parce que une hémiplégie droite est rarement. Enfin, ils ont des douleurs, mais moins. Moins moins perturbante que les hippies gauche ? Je pense que c'est important dans le rapport d'avoir cette dissertation. Je sais pas si d'autres mes autres confrères l'évoquent mais. Il y a quand même cette cette différence de l'hémiplégie gauche, de l'hémiplégie droite, hein, quand même. L'hémiplégie gauche à des troubles des douleurs neurologiques qui sont. Qui sont qui peuvent être invalidants pour le travail de l'intensité.

BJM

Pensez-vous que leurs troubles cognitifs ou bien leur fatigue ou bien leur handicap peuvent constituer un soin ? Pour ces patients-là.

K1

Oui, oui, je pense oui.

BJM

À votre avis, est-ce plus facile ? Vous vous d'intensifier les séances de kinésithérapie à la phase chronique de l'AVC ou bien qu'en phase aigu et pourquoi ?

K1

Ben en phase aiguë en fait en cabinet libéral, c'est rare, on en a rarement, hein, donc. Et donc j'ai peu, j'ai j'ai peu de retours là-dessus, je peux pas trop vous, moi je les, je les vois. Après, il y a les oui. Si les aînés, les enfants, un AVC en phase aiguë, ils sont-ils sont plutôt à l'hôpital ou en centre de rééducation. Nous, on les voit plutôt. À distance, il y a toujours des choses, il y a toujours des choses qui progressent. Et c'est ça qui est intéressant. En fait, il y a toujours une évolution favorable. En phase chronique donc, la la le le frein c'est que je non je j'en vois quasiment pas de en phase aiguë et. Voilà, oui, je crois que j'ai.

BJM

Oui, selon vous, l'intensification des séances est-elle importante dans la rééducation des patients pour service ?

K1

J'ai pas entendu le début de la phrase.

BJM

Pensez-vous que l'intensification des séances est importante pour la rééducation des patients, pour sa vessie ?

K1

Est-ce que l'intensité est bien est bien ?

BJM

Est importante, de la rééducation des patients post AVC.

K1

Oui, oui, oui, tout à fait. Tout à fait.

BJM

Et d'après vous, doit-on l'intégrer à la pratique de routine et aux traitements kinésithérapie ? Ou bien Pensez-vous que cela devait faire l'objet de séances complémentaires comme des séances d'activités physiques adaptées ?

K1

Non, non non. Enfin, je pense qu'il faut on. On est dans la rééducation donc. Après, ça peut être dans dans dans les maisons de retraite peut être être intéressant d'être en complément. Que il y a un complément d'activité physique adapté ? Mais non, je pense que il faut-il faut quand même qu'on qu'on garde, qu'on préserve notre notre acuité professionnelle là-dessus et donc non, non non, c'est important de de préserver cette activité kinésithérapique là.

BJM
Donc ou pensez-vous qu'il est de votre ressort de faire faire des activités intenses à vos patients ?
K1
Oui, tout à fait. Oui, tout à fait.
BJM
Donc en dehors des séances au cabinet prescrivez-vous des exercices intenses que le patient doit faire chez lui ? Lui donnez-vous une dose particulière en termes d'intensité.
K1
Je fais peu de je fais peu d'éducation thérapeutique, chose qui est pas forcément bien, mais j'avoue, hein mais. Si j'ai je, je les invite à marcher régulièrement, à faire du vélo d'appartement Ouais et cetera, donc je les invite un peu à la, j'en ai un justement qui va en acheter un bon. Ben je je l'ai conseillé sur le matériel, on a on a échangé et puis après le fait de le voir quand même régulièrement à chaque fois je leur dis tiens mais il votre vélo appartement vous en avez combien de temps ? Oui c'est bien, allez, il faut continuer. Voilà, il y a le côté un peu mais d'exercice proprement dit. Pardonne pas.
BJM
Avez-vous peur des conséquences qui pourraient qu'il pourrait y avoir chez ces patients si vous intensifiez les exercices ? Lesquels, par exemple ?
K1
Oui, je vois à peu. Près la le le le question la question je. En fait, moi j'en prescris, j'en prescris pas parce que comme je les vois quand même assez souvent. Déjà, au moins qu'ici le fasse correctement. Il y a, il y a quand même le le regard du kiné donc c'est. Voilà, moi je. Je pense que après. C'est pour les kinés, pour les les patients qui arrêteraient. Voilà alors prise en charge, donc là, oui. Là je leur donnerai éventuellement des conseils de mouvement, mais comme on les voit quand même assez régulièrement. Bon, mais ça permet d'avoir une une continuité dans nos dans notre séance, dans dans la prise en charge.
BJM
Donc, c'est la fin de l'entretien, je vous remercie pour ce temps d'échange, souhaitez-vous ajouter une dernière remarque concernant le thème ou avez-vous des questions à me poser
K1
Je pense que la base quand même de je reviens sur le début de notre entretien, c'est l'espace. Voilà et je pense que d'avoir un espace adapté. Permet de libérer le kiné. De d'une crainte de chute et mais. Mais le kiné est plus à l'aise. Et et le patient, le patient aussi, dont je pense que l'espace, il est primordial.

II

Retranscription de l'Entretien 2 (enquête 2, MK2)
Entretien réalisé le 4 avril 2023
Durée 24 minutes 14 secondes
Identification des participants : Bénédicte JEAN MICHEL, Masseur-Kinésithérapeute 2(MK2)

Fichier audio

audio1275462462.m4a

Transcription

BJM

Donc pour débuter cet entretien, puis s'il vous plaît, vous demandez de vous présenter brièvement votre âge, votre parcours professionnel, votre formation et si vous avez eu besoin de formations complémentaires ?

MK2

Oui, alors ? Je suis désolé, ça frise l'image, frise un. Petit peu, mais. Donc j'ai j'ai 45 ans, j'ai eu mon diplôme de kiné et de Masseur kinésithérapeute en 2001. Depuis 2002, je travaille au sein de cabinet de rééducation libérale spécialisée ou qui ont la spécificité d'exercice en neurologie centrale. On accueille donc des patients qui ont des hémiplégies, maladie de Parkinson, sclérose en plaques. Lésion médullaire et traumatisme crânien et et paralysie cérébrale principalement. Au niveau de mon parcours, Ben j'ai fait un diplôme universitaire de communication médicale scientifique en 2007, un diplôme interuniversitaire de qui porte sur les maladies neurodégénératives en 2022 et différente de formation de 2 ou 3 jours dispensés via le. Le financement des PC ou le financement fpl enfin sur différents sujets. Et après là, actuellement, je suis en master 2, aller à la faculté de Grenoble, master 2, ingénierie de la santé, voilà, je suis actuellement étudiant. Tout en continuant mon exercice libéral, voilà.

BJM

D'accord ! combien de temps exercez-vous en libéral ?

MK2

Et ça fait 22 ans.

BJM

22 ans ! Donc, sur 100 patient que vous voyez, combien de patients post AVC avez-vous dans votre patientèle ?

MK2

J'irai 20% à peu près 1/5.

BJM

Combien de fois par semaine les voyez-vous et combien de temps ?

MK2

En moyenne 2 fois, ça peut aller de une à 2, une à 4 pour les les patients qui sortent de centre et la durée, c'est en général les séances. D'une heure et. Demie pour les personnes âgées, post AVC en phase chronique, ça peut être 2 séances d'une heure éventuellement.

BJM

Vous les voyez en groupe ou en individuel ?

MK2
En groupe, c'est des prise en charge de groupe.
BJM
Donc les voyez-vous, c'est une durée ? Limitée de séance ou bien en continu ?
MK2
Ah, je suis désolé, je n'ai pas entendu la fin ou bien ?
BJM
Ou bien continue ?
MK2
Ah, c'est en continue, ce sont des prises en soins continue. Pour la plupart, ouais.
BJM
D'accord donc, à la suite des résultats préliminaires de l'étude Observationnelle. Que vous avez. Participé avant nous avons remarqué que l'intensité des séances différait de patients à un autre. Quel regard portez-vous sur l'intensité des séances de kinésithérapie ?
MK2
Quel regard c'est à dire vis-à-vis de mon travail, si ?
BJM
Oui, quelle importance ? L'intensité des séances a par rapport à votre travail?
MK2
Alors ce Ben d'après les recommandations de la HAS, je dirais que c'est très important que la rééducation post AVC soit intensive, intensive, avec une fréquence déterminée et si possible continue quand il y a des lésions et des des déficits qui sont qui sont chroniques.
BJM
Qu'entendez-vous par intensité des séances chez les patients post AVC en phase chronique?
MK2
Il faut qu'il y ait un bon volume d'exercices et des exercices à la fois, un volume, un volume intéressant et une une dose intéressante. Nous en fait nous, avec mon associé, on fait sur 1h30. En général, on fait une demi-heure de réentraînement cardio-respiratoire en utilisant des différents appareils, ce soit un cyclo ergomètre à bras ou un cyclo ergomètre à. Ou ou alors un vélo elliptique ou un tapis roulant. Après on, on a, on alterne avec des séances de gymnastique de groupe avec musculation un petit peu type Crossfit, on va dire mais adapté. Travail de descente, relevé du sol et ensuite un un exercice de marche sur 1 km. En fait en en extérieur. Chronométrer en fait, donc un millimètre chronométré à viser à la fois un petit peu cardio respiratoire et beaucoup fonctionnel aussi. Voilà donc l'intensité. Il faut que les patients Ben ayant participé à l'étude, j'ai envie de répondre que il faut que en séance de kiné il dépense beaucoup de de METS qui soient au-dessus de leur, de leur métabolisme basal le plus longtemps possible pendant la séance quoi ?
BJM
À part les recommandations de HAS, est-ce que vous connaissez d'autres recommandations en termes d'identité d'exercice pour cette population ?
MK2
D'autres recommandations en termes d'intensité d'exercice. De mémoire comme ça, non mais.
BJM

D'accord, donc, à votre avis, la séance que nous avons observée avec votre patient, était-elle intense ou pas du tout ? Est-ce qu'elle était modérée ou faible ou légèrement ? Pourquoi

MK2

À mon avis, elle était intense.

BJM

Pourquoi ? Elle était-elle intense ?

MK2

Pour les les. Comment dire le à en raison du descriptif que j'ai évoqué ? Un petit peu plus. Tôt avec la demi-heure de cardio, les exercices de gym et. Et la marche au parc.

BJM

D'accord, selon vous, quel est l'environnement technique, matériel et professionnel nécessaires à la mise en œuvre d'intensification des séances de rééducation des patients post AVC ?

MK2

Alors moi je pense qu'il faut un, il faut un plateau adapté, il faut un un plateau technique avec des appareils à la fois accessibles et sécurisés dans un espace. Un espace adapté dans lequel les patients peuvent, si possible, être le plus autonome possible et en même temps travailler de bah de manière intensive et en et de de façon sécurisée, sans risquer de tomber ou de se blesser ou de se faire mal.

BJM

Selon vous, quels sont les points ou obstacles environnementaux à sa pratique et quels sont les facteurs facilitant ?

MK2

Frein environnemental. Alors il y a le, les transports, la question des de la prise en soin, de la prise en charge des transports conventionnés qui est un problème souvent en kiné libéral, c'est le domicile qui est privilégié parce que. Ce pour des questions de de facilité. Sauf que en en déplacement à domicile, un kiné ne peut rester qu'une demi-heure chez le patient et du coup il peut pas pratiquer une grande variété d'exercices. Il y a la question des transports. Après il peut y avoir des freins liés aux patients, des freins psychologiques, la difficulté à sortir de chez soi, la fatigabilité une trop grande fatigabilité ou ou Des problèmes d'accessibilité du cabinet, par exemple. Et après ? Donc ça c'est plutôt les freins liés aux patients, les freins à l'environnement et au contexte sanitaire. Et puis après il doit y avoir des freins. Ouais, je pense que c'est. À peu près tout.

BJM

Alors, par rapport à l'intensité de vos séances, le manque d'espace disponible Et accessible est-il un obstacle pour vous ?

MK2

Mais du coup pas trop pour nous puisqu'on a une grande salle en fait avec ma collègue et qu'on prend les gens en groupe limité justement pour pas avoir trop de monde en même temps pour des questions de sécurité, pour des questions conventionnelles évidentes et puis aussi pour des questions de de de qualité, de travail en fait. Et si on a trop de patients en même temps dans la salle, les appareils ne sont pas disponibles, on peut pas bouger, ils ne peuvent pas bouger, ils peuvent pas se. Déplacer, donc c'est. C'est rédhibitoire, ça, ça limite ça limite notre travail.

BJM

Et par rapport au temps, le temps disponible dans une seule séance de traitement, constitue elle un obstacle?

MK2

Et Ben, par groupes d'une heure et demie pas trop. Ce qui est gênant alors ça, c'est comment dire le kiné libéral qui qui parle, mais du coup comme on fait des soins chroniques avec sur le sur, sur la durée, il arrive que certains patients loupent la séance. Parce qu'ils ont un rendez-vous médical parce qu'ils sont pas bien, et cetera, qu'ils préviennent ou pas, ça, c'est une autre question. Mais du coup, on a un problème de de rentabilité entre guillemets, c'est à dire que. Ben du coup sur sur un créneau d'une heure et demie où normalement on voit, on est censé conventionnellement programmer 3 patients souvent en programme. 4 comme ça, s'il y en a un qui fait défaut, ce qui arrive régulièrement, il n'y a quasiment jamais de journées qui passent sans qu'il y ait au moins un ou 2 absents dans la journée. Du coup, on en programme 4 sur les créneaux d'une heure et demie comme ça, on est quasiment sûr d'avoir tout le monde en fait.

BJM

Donc, idéalement, combien de temps vous faudrait-il ?

MK2

Idéalement 1h30 déjà c'est pas mal, on peut-on peut faire pas mal de choses, 2h ça serait bien mais 2h, ça impliquerait qu'on ait qu'on ait encore plus de monde dans la salle en même temps pendant un temps encore plus long, c'est. Un peu compliqué, c'est ce qu'on faisait avant la avant le confinement avant la crise COVID, on faisait des créneaux de 2h en même temps, mais. Du coup, il y avait un petit peu de monde dans la salle et c'était pas très confortable. Et depuis qu'on est passé en fait, on est passé à 1h30 1h. C'est à dire que quand quand un de nous 2 reçoit des gens pendant 1h30, l'autre en face les reçoit pendant 1h et du coup on alterne comme ça, donc on essaie de varier un peu. Les groupes de. Mixer un peu les groupes et à l'exception notable du groupe du matin, le groupe de 8h30 à 10h qui là est composé plutôt de personnes très, très actives et par définition les gens qui peuvent se lever tôt seuls, qui sont suffisamment autonomes pour venir en kiné par leurs propres moyens. Voilà mais sinon le reste de la journée en fait ma collègue et moi on alterne pour se retrouver avec maximum 7 personnes en même temps dans la pièce. Donc, soit l'un 4 et l'autre 3 ou inversement. Comme ça, ça n'a ça ne. Ça ne pénalise pas trop les séances des patients voilà.

BJM

D'accord ? Comment sélectionnez-vous les patients à ? Qui vous faites faire de l'exercice plus intense.

MK2

Ah, en fonction de leur capacité. Globale, voir s'ils arrivent à s'ils peuvent rentrer dans le groupe de 8h30 10h, c'est-à-dire avoir une marche extérieure autonome et sécurisée. Je ça et puis. Après, je dirais que autant que possible, on essaie de faire de l'intensif. Au plus grand nombre de gens possibles, c'est-à-dire même quelqu'un qui a des problèmes cardiaques, des problèmes respiratoires, et cetera. On va quand même lui faire faire de l'actif, on va faire alors après ce qu'on peut qualifier ça d'intensif Ben par rapport au on va dire aux capacités basales de la personne on peut être mais. On va, on fait que de l'actif en fait, mon associé et moi, on essaye au maximum que les gens. Utilisent leur utilisent leur. Enfin dépense des calories au cabinet, quoi faut vraiment qu'ils puissent faire ici ce qu'ils peuvent pas faire à l'extérieur, faire du du, la tous nos patients ? Ils font du réentraîne cardio, respire quasiment tous, voilà.

BJM
Donc pour ces patients là, ce que avez-vous besoin qu'ils aient effectué une épreuve d'effort ou bien que le médecin vous ait donné l'autorisation dans cette épreuve.
MK2
Alors, lors du bilan lors de l'anamnèse, on demande systématiquement aux gens s'ils ont des problèmes cardio-respiratoires et on leur demande aussi d'amener leurs examens complémentaires. Et puis mais à partir du moment où le le médecin prescrit, de la kinésithérapie, sans nous imposer, c'est-à-dire sans faire une prescription qui soit à la fois qualitative où quantitative, à ce moment-là on a le champ libre pour pour faire les. Exercices qu'on souhaite. Voilà, je crois que.
BJM
Évaluez-vous l'intensité des séances chez vos patients si oui, comment paramétrez-vous ces séances ?
MK2
Alors souvent, on utilise le test 2000 M en extérieur pour voir si le patient progresse ou si si ces capacités ont atteint un plateau et régulièrement on refait aussi les bilans qu'on fait en début de rééducation, alors ce sont des bilans de force, de d'équilibre, de changement de position. Voilà donc on essaie de les refaire, on les refait à la demande parfois quand les les patients en éprouvent le besoin, en en signifie la nécessité. Et sinon, on essayait de les faire au moins une fois par an pour pour évaluer un petit peu sauf quand ce sont des gens qui sortent juste de centres de rééducation qui viennent juste d'avoir un AVC, qui sont pas encore en phase chronique à ce moment-là, on les fait plus régulièrement, les les évaluations. Mais quand les gens sont passés en face chronique, c'est à dire plutôt au-delà d'un an. Post AVC on refait les tests que une fois l'an, une fois tous. Les 6 mois, ouais.
BJM
Donc quels sont les outils d'entraînement utilisés vous habituellement ?
MK2
Alors entraînement, bah tapis roulant, vélo elliptique, vélo, vélo d'appartement, cyclo ergomètre, cyclo ergomètre à bras, on a un banc de musculation et voilà.
BJM
Adaptez vous l'intensité des exercices en fonction de l'état physique du patient. Comment ajustez vous les séances en matière ? De durée, d'intensité, de fréquence
MK2
En fonction de la fatigue du patient et voir si en fonction de sa plainte si l'exercice devient trop pénible, le bon indicateur, c'est que ils aient, ils aient transpiré pendant la séance et qu'ils ils aient un peu qu'ils étaient soufflés pendant le pendant l'effort, quoi, voilà.
BJM
Donc leur faîtes vous faire un test d'effort maximum sous maximal comme le test. De la marche de 6. Minutes par exemple
MK2
Alors ça, c'est vrai qu'on le fait pas. Régulièrement, on le fait en début de rééducation souvent, mais après, on utilise le millimètre comme indicateur.
BJM

quels critères de surveillance, utilisez vous lorsque vous êtes densifier les séances, est-ce que c'est la fréquence cardiaque, la saturation ou la perception d'effort Ou un autre ?
MK2
Juste la, juste la perception du patient. Comprend, ouais.
BJM
Donc, selon vous, quelles sont les connaissances théoriques, techniques et pratiques dont doit disposer le kinésithérapeute pour intensifier la séance de rééducation ?
MK2
Pour intensifier. Ah Ben après. Le la, la boîte à outils de base du kinésithérapeute, renforcement musculaire et entraînement à l'effort. Ce qu'on voit à l'école, ça peut s'appliquer tout aussi bien aux personnes post AVC. Il faut juste être un peu vigilant. Enfin d'éventuels aux traitements et aux complications associées ou ou aux crises d'épilepsie, s'il y en a. Mais sinon, c'est on fait exactement les mêmes exercices qu'avec des patients valides, des personnes qui font de la rééducation en traumatologie, des choses comme ça, sauf qu'on l'adapte, on adapte les mouvements aux capacités motrices de la personne et par exemple, si je si je dois illustrer ça avec un exemple, si la personne ne peut n'a que une abduction du bras à 4. Au lieu de 5. Ben, on l'a fait travailler dans dans toute l'amplitude mobilisable avec un peu de poids pour quand même renforcer le parti le. Le membre physique, voilà.
BJM
Donc l'information initiale, vous pensez que l'information initiale suffit pour le kinésithérapeute ?
MK2
C'est une bonne question...je... comme mes études datent. Un peu, je. Je saurais pas trop répondre j'ai un double biais face à cette question, c'est que je suis formateur avec mes associés. Du coup j'ai la prétention de croire que dans les écoles. On intervient-il ils ? Ils savent ce que c'est que l'intensif en kinésithérapie neurologique et après ouais, comme je vous disais mes études. Datent un peu, donc je suis. Dans les autres écoles, je saurai, je pense quand même oui que que que c'est uniformisé au niveau au niveau des écoles, de la métropole et que. Le message maintenant est bien diffusé. Ouais, quand j'étais étudiant, on nous disait que il fallait rééduquer la, la gestuelle qu'il fallait améliorer, le schéma de marche et cetera. Et ça, je crois que c'est, c'est vraiment tombé en désuétude. Et à juste titre ?
BJM
Donc, pour vous, l'information initiale était était ? C'est suffisant ?
MK2
Je pense qu'elle est adaptée. Ouais, elle est adaptée.
BJM
Donc avez-vous besoin d'une formation complémentaire ou bien la formation initiale était ?
MK2
Alors peut-être pas pour les questions d'intensité. Pour cette question là précisément, je pense que le message passe bien dans les. Écoles de kiné. Mais c'est très subjectif hein, je je suis vraiment pas sûre de ma réponse. Par contre il y a plein de il y a plein d'autres aspects qui nécessitent un approfondissement je pense. Après les études, les études de kiné. Comme le trouble associé les les fonctions supérieures, les chirurgies, l'appareillage, tous les à côté en fait de de la rééducation, des personnes post AVC.
BJM

D'accord. Est-ce qu'il y a des freins concernant les patients à faire de l'exercice plus intense ?
MK2
Oui, je pense plein. Plein parce que parfois Ben en post AVC on. Ce sont des gens qui ont des troubles associés comme de l'hypertension, du diabète, des choses comme ça qui ont déjà pas pas ou peu d'appétence à à l'activité physique ou qui en prennent pas ou qui y prennent pas beaucoup de plaisir. Du coup, il faut essayer de les y amener un petit peu. Je pense, ouais, je pense que ça dépend un petit peu de la. Population qui est plus sujette à faire des AVC sauf dans le cas des des personnes qui ont des anévrismes. On a quelques patients, alors y a quelques patients anciens sportifs en fait, qui ont fait une rupture d'anévrisme et cela au cabinet. Ils adhèrent bien un programme intensif. Après, il y a des gens aussi, on a eu quelques patients qui ont eu des hémiplégies gauches avec un tableau d'apathie, d'apathie neurologique, ancien sportif et qui du coup, ont eu du mal à, ont eu du mal à mais à quand même. À fournir un. Un travail intensif pendant la pendant les séances de Kiné Ouais.
BJM
Et Ben pour les patients post AVC, est-ce qu'ils répondent à l'exercice ? Plus intenses ?
MK2
Un. Est-ce qu'ils y répondent dans le sens, est ce que c'est efficace selon ? Oui, moi je pense de. Toute façon, je pense que c'est efficace dans quasiment toutes. Les pathologies. Mais oui, aïe. Enfin moi, je j'ai l'impression qu'ils y répondent oui. En tout cas.
BJM
Est-ce que la sévérité de leur. Handicap est-elle un frein ou bien ? leurs troubles cognitifs ? ou alors leur motivation ? ou leur fatigue. Peuvent constituer un frein.
MK2
Oui, oui, tout à fait. Si l'hémiplégie est massive, avec une placidité de tout le, de tous les hémicorps. Là, c'est plus compliqué de de de proposer de l'actif s'il y a des troubles associés comme une aphasie sévère mixte ou une une grosse apraxie ou des choses comme ça. Effectivement, un peu plus de. On a beaucoup plus de difficultés à faire travailler les gens.
BJM
À votre avis, est-ce plus facile pour vous d'intensifier Les séances de. Kiné à la phase chronique de l'AVC ou en phase Aiguë et pourquoi ?
MK2
Ah non, pour moi il n’y a pas de il y a pas de question de de de phase que il faut essayer d'être intensif le plus tôt possible et le plus longtemps possible. Donc moi ce n’est pas une difficulté, non.
BJM
Selon vous, l'intensification des séances était l'importante de la rééducation des patients post AVC ?
MK2
Oui, très selon moi, très ouais.
BJM
Et doit-on l'intégrer Dans la pratique de routine ?
MK2
Ah oui, si autant que possible, parfois en face chronique, quand les patients ont une activité physique à côté, style marchandises ou des choses comme ça. C'est l'exemple qui me. Vient à

l'esprit mais. Eh Ben on supprime une séance de kiné sur les 2 restantes en phase d'entretien et on fait plus qu'une séance de kiné par semaine. Si ils ont une activité physique à côté. Ouais.

BJM

Pensez-vous qu'elle est de votre ressort de faire faire des activités intenses à vos patients ou habituellement ? Qui prescrit les exercices intenses aux patients ?

MK2

Je pense que c'est notre de notre ressort. Je alors je, je crois pas qu'on ait le droit de prescrire de l'activité physique adaptée. Il me semble pas.

BJM

Est-ce que selon vous c'est le kiné qui devrait. Qui devrait prescrire ce genre d'exercice ? selon vous?

MK2

Ah oui, ça pourrait pas évidemment le le le. Comment dire. L'indication médicale vient tout de suite à l'esprit, mais je pense que le Kiné pourrait si au bout de une quinzaine ou une vingtaine de séances, on voit que le patient répond, répond bien à l'intensité et aux exercices intensifs. Nous, on les, on les incite constamment à marcher en dehors des séances de kiné, à faire de l'activité. Mais oui, oui, oui, je pense que kiné il pourrait.

BJM

En dehors des séances au cabinet prescrivez ou des exercices intenses, le patient doit. Faire chez lui ? Lui donnez-vous une dose particulière?

MK2

Comme ça ? Non pas des exercices comme je vous disais, plutôt des activités style marche natation s'ils aiment ça ou toute autre activité, et alors le le critère de choix aussi, c'est le côté ludique. Il faut que la personne aime ça, faut pas que ce soit une contrainte parce que sinon c'est c'est rapidement, c'est souvent rapidement abandonné en fait. Mais oui, on a alors on on, c'est assez rare les ce qu'on peut proposer éventuellement, c'est des des auto-postures, des choses comme ça, sinon des exercices type musculation, poids du corps ou ou entraînement cardio à la maison. C'est plutôt rare, souvent moi, alors c'est par par par goût personnel, parce que j'aime bien la marche, j'ai plutôt. Tendance à proposer la marche ? Aux gens en fait, voilà.

BJM

D'accord ? Avez-vous peur des conséquences qu'il pourrait y avoir chez ces patients si vous intensifiez les les séances ?

MK2

Enfin, clairement non.

BJM

On, c'est la fin de l'entretien, je vous remercie pour le temps accordé, donc souhaitez-vous ajouter une dernière remarque ? Ou une question à me poser ?

MK2

Non, j'ai pas de j'ai pas de questions qui ? Viennent à l'esprit mais.

BJM

Bon, pas de remarque non plus?

MK2

Pas de remarque, non aucune, si je peut être juste une, pas une question, mais une demande plutôt Ben c'est avoir si possible un un exemplaire à la à la fin de votre étude pour pouvoir le le consulter.
BJM
Eh oui oui, alors vous aurez des résultats de cette nouvelle étude.
MK2
Ça m'intéresse.

III

Retranscription de l'Entretien 3 (Enquête 3, MK3)
Entretien réalisé le 4 avril 2023
Durée 29 minutes 30 secondes
Identification des participants : Bénédicte JEAN MICHEL(BJM), Masseur-Kinésithérapeute 3 (MK3)
Fichier audio
audio1151286257 1.m4a
Transcription

BJM
Pour débuter cet entretien, puis-je s'il vous plaît, vous demandez de vous présenter brièvement, votre âge, votre formation, votre expérience professionnelle et votre date de diplôme. Et si vous avez suivi des formations complémentaires.
MK3
Alors moi je suis. Donc, kinésithérapeute depuis 97. J'ai 48 ans donc je m'appelle X, je travaille à Périgueux dans un cabinet de groupe où on est 4 kinés, 2 podologues et 2 infirmières. Moi, je me suis spécialisée. Bon toute façon, on est obligé de faire des formations, donc on a suivi des formations régulières un petit peu surtout, et en dernier moi, j'ai fait beaucoup de formation sur du Maxillo-faciale donc pas obligatoirement en rééducation neurologique. Mais la formation fait qu'on a quand même des patients neurologiques.
BJM
D'accord, depuis combien de temps exercez-vous en libéral ?
MK3
En libéral depuis 99 depuis 1999.
BJM
Donc, sur 100 patients, combien de patients post AVC Avez-vous dans votre patientèle ?
MK3
Donc j'en ai très peu là en ce moment puisque comme je vous dis, je me suis spécialisée en maxillo-facial donc tout ce qui est rééducation de la face, je dois en avoir on va dire que je dois bien avoir 100 patients, j'ai 2 patients, donc je ne sais pas exactement si on dans 100 ou 200, mais je dirais on va dire 2%.
BJM
D'accord donc, sur ces 2 % de patients que vous avez, combien de fois par semaine les voyez-vous et. Combien de temps ? Est-ce que c'est en groupe ou un individuel ?
MK3
Alors il y en a une des 2 qui est qui a été vue par Stéphanie, que je vois 2 fois par semaine et l'autre qui est plus jeune et qui n'a pas voulu faire le test, je le vois une fois par semaine mais il a 19 ans. En ce moment donc, il fait quand même du sport à côté. Il a ses études et tout. Donc c'est plus un choix de sa part de de se voir une fois par semaine que plus voilà.
BJM
Donc vous les voyez plus en individuel qu'en groupe ?
MK3
Alors on commence par de l'individuel, ils sont quand même dans une salle où il y a du monde, mais leur traitement est uniquement individuel, ce n'est pas... Voilà, on ne fait pas travailler tout le monde en même temps. Ils peuvent être avec d'autres patients qui sont juste à côté dans la grande

salle pour faire un peu. De groupes, effets, groupes, puisqu'ils peuvent parler aux autres, mais leur traitement est totalement individuel.
BJM
Donc voyez-vous sur une durée limitée de séances ou bien en continue ?
MK3
En continue.
BJM
Donc, à la suite des résultats préliminaires de l'étude observationnelle à laquelle vous avez participé, nous avons remarqué que l'intensité des séances différait d'un patient l'autre. Quel regard portez-vous sur l'intensité des séances de kinésithérapie ?
MK3
Alors, le souci, c'est toujours le patient en fait hein, le kiné, peut-être hein, mais surtout le patient, moi, entre les 2 par exemple, je sais que... J'ai un patient donc qui est jeune, plutôt motivé. Donc on peut faire des séances assez sportives donc assez dynamiques hein au contraire ou on peut faire un peu du basket ? Enfin des choses comme ça, donc très dynamique quand même, plus avant d'ailleurs. Et l'autre dame qui avait été suivie, par exemple, qui est plus dans le refus facilement, dans le refus et capable de le faire, beaucoup mieux, mais qui ne veut pas faire grand-chose, donc là, automatiquement l'intensité, ben on s'adapte un petit peu. Et puis j'aurais tendance à dire que si on réussit à lui faire faire quelque chose, des fois c'est déjà bien. Et parfois, elle est, elle est pas motivée du tout. Donc automatiquement ça dépend franchement de la motivation et de de ce que le patient veut vraiment quoi.
BJM
D'accord, qu'entendez-vous par intensité des séances chez des patients post AVC en phase chronique ?
MK3
Alors bah l'intensité je vous dis c'est pareil, toujours sur la même chose. C'est en fait en fonction du patient, en fonction de ce qu'on va pouvoir lui faire faire donc. De son âge, de son bah, du handicap, de la récupération qu'il a eue et après ben on va au maximum de ce qu'il peut faire. Enfin, je pense. Je ne sais pas si j'ai bien répondu.
BJM
Non, il n'y a pas de bonne ou de mauvaise réponse, c'est votre expérience qui parle. Quelle importance accordez-vous à l'intensité dans la mise en place de vos séances et pourquoi ?
MK3
Alors je vais tendance à dire que d'abord, on commence par un échauffement comme n'importe quel entraînement, entre guillemets, hein, pour réchauffer, pour prendre un petit peu l'attitude du patient ce jour-là, s'il a des douleurs, si voilà. Et puis on monte en intensité quand même au maximum de ce qu'on va pouvoir faire. Moi, ce sont des 2 patients qui sont... Il y en a un an donc, à 19 ans, mais c'était de naissance, donc ça fait longtemps et l'autre elle a 42 ans je crois et ça fait plus de 15 ans elle aussi qu'elle a eu son AVC. Donc on peut se permettre de travailler en intensité. J'aurais tendance à dire maximale. Tout est relatif, mais on peut faire énormément de choses. Contrairement aux phases aiguës ou il faut faire attention à pas mal de. Choses, mais nous, on peut faire vraiment tout ce qu'on veut. Puisqu'il n'y aura pas d'effets secondaires entre guillemets sur la récupération.
BJM
Donc, connaissez-vous des recommandations en termes d'intensité des séances pour cette population ou pas ?
MK3

Alors au début, oui. Et on a pas mal, hein ? Pas mettre de poids, des choses comme. Ça, on. Secondaire là où moi j'en suis rendue, j'aurais tendance à dire que non.
BJM
D'accord, à votre avis, la séance que nous avons observée avec votre patient a été intense ou pas du tout ou légèrement ?
MK3
Ouais, elle était intense. Euh non, elle peut faire beaucoup mieux. Mais on l'a vu après dans les résultats, quand on voyait. Stéphanie malgré tout. Pour elle c'était une intensité importante puisque si j'ai lu hein, j'ai lu entre les lignes, mais moi elle ne fait rien du tout. Elle est plutôt inactive sur les 3/4 du temps en dehors donc c'est malgré tout très important qu'elle vienne. Au cabinet pour ben pour faire quelque chose, sinon je crains qu'elle ne finisse limite dans son fauteuil puisque nous elle refuse de marcher à la maison. Donc l'intensité pour moi n'est pas du tout suffisante, mais pour elle, c'est peut-être déjà énorme.
BJM
D'accord, selon vous, quel est l'environnement technique, matériel et professionnel nécessaire à la mise en œuvre d'intensification des séances de rééducation des patients post AVC en phase chronique ?
MK3
Du matériel, il en faut. Quand même parce que si on veut, cela dépendra de quelle phase bien sûr, mais il faut du matériel, ça, c'est certain. Après, en fonction de la récupération, marcher, ça peut être important. Parfois, ça peut être des aides mécaniques. Je pense qu'on peut faire sans rien, mais que le vélo, des choses comme ça. Tout ce qui est au niveau cardio, qui va aider aussi. Après je ne suis pas spécialisée dans le dans la Neuro donc il doit bien y avoir des choses très très techniques, mais je pense qu'on peut faire de la neuro quand même, bien avec peu de choses. Le Kiné, tout seul avec ses mains ces choses comme ça, on peut faire pas mal de choses, pas tout, hein, bien sûr, mais je pense qu'on peut faire mal de choses.
BJM
Selon vous, quels sont les freins ou obstacles environnementaux à sa pratique et quels sont les facteurs facilitants ?
MK3
Alors environnementaux, Ben ça dépend de plein de choses, même si c'est pas l'environnement. Ben la personne, hein. En fonction de sa motivation, son âge et ainsi de suite, sa récupération. Après son habitat enfin, si c'est adapté ou si ce n'est pas adapté quand il est chez lui, hein ! Après, chez nous pareil hein, mais normalement on est censé être adapté à ça, ça serait mieux. En facilitant, moi, je trouve que le fait que les patients ne soient pas tout seuls, ça leur fait du bien d'avoir du monde à côté pour parler pour échanger et puis pour voir aussi que les autres travaillent, que donc eux peuvent travailler Et se comparer, voir qu'ils sont parfois meilleurs. C'est important aussi de savoir qu'on est bon dans quelque chose que d'autres sont moins bons, même s'ils ont des handicaps ou des pathologies qui sont différentes. Donc en facilitant, je dirais le groupe et la motivation. Et pour plutôt dans l'aspect négatif Bah déjà avoir oui en effet, les bonnes chaussures. Enfin tout ça sert aussi et surtout bah l'environnement familial et sa motivation là aussi, mais dans le mauvais sens.
BJM
D'accord par rapport à l'intensité de vos séances, le manque d'espace disponible et accessible, est-il une limite ?
MK3

Non, parce que nous, on a un cabinet qui est suffisamment grand, on a un grand couloir donc on peut faire des choses et on a un grand parking et donc on va dehors régulièrement. Donc non moi je n’ai pas de problème mais ça peut l'être hein. Si on a un tout petit endroit, ce sont des patients qui restent longtemps. Moi elles restent au moins 1h30, même si ce n’est pas grand-chose. Elle reste 1h30 au cabinet. Donc elle a le temps de faire énormément de choses et il me faut de la place parce qu'il faut bien voir d'autres patients. En attendant, on ne peut pas, on ne peut pas vivre entre guillemets avec un patient par 1h par 1h30, donc il faut de la place mais moi je n’ai pas ce problème là puisque on a l'avantage d'être à Périgueux et d'avoir des locaux qui sont suffisamment grands.

BJM

Quand vous dites un espace grand, d'après vous, combien il faudrait pour le kiné ?

MK3

Alors ça dépend du kiné, nous on est, on est 4 donc. Nous, on a 200 m², plus le parking, plus après on peut partir sur la route puisqu’il n’y a pas grand monde, ça la route, c'est sur le trottoir. Pour éduquer, il faut quand même de la place, surtout si on fait ça en en activité principale. Et ce qu'il faut quand même un pouvoir, les mettre sur la table Bobatt. J'aurais tendance à dire que... Il faut je n’en sais rien, c'est assez dur à dire. Je ne me rends pas bien compte en plus, je n’ai pas trop trop la notion de l'espace, mais une pièce de moins de 50 m² ça me semble compliqué. Non, on peut tout faire, hein ! On peut faire mieux, mais je pense que moins de 50 m², si on veut les faire bouger et puis... Ouais, je dirais ça.

BJM

Le temps disponible dans une seule séance de traitement de kiné constitue-t-elle, un obstacle ?

MK3

Non plus, si le patient est motivé, s'il travaille tout seul il y a beaucoup de choses qu'on fait Sous la direction du Kiné mais où on n'a pas besoin du Kiné, donc les séances de groupe, entre guillemets, ça peut être ça aussi où on peut être à plusieurs. Mais le kiné s'occupe de l'un, de l'autre, ça permet d'avoir l'œil du Kiné qui reste dessus. Mais pas obligatoirement, qui s'occupe que de la patiente, du patient. C'est pareil, ça dépend vraiment de à quel moment de tout plein, plein de choses en fait, mais je pense que ce n’est pas limitant, ne faut pas en prendre 50 en même temps, mais ce n’est pas obligatoirement limite. Ça peut ne pas être émis.

BJM

Comment sélectionnez-vous les patients à qui vous faites faire de l'exercice plus intense ?

MK3

Ben comme je vous disais, en fait, au début de la séance, on prend un petit peu la température du patient, s'il est bien, s’il n’est pas bien. En fonction comme je vous dis de l'âge, de la pathologie, de la récupération. Après c'est, c'est le bilan en fait hein ! C'est le bilan qu'on a déjà fait au début et le bilan qu'on fait en fait à chaque séance pour savoir ce qu'on va pouvoir lui faire faire. Et puis c'est dire puisqu'on va quand même l'écouter s’il dit que c'est trop trop et qu'on voit que c'est trop, hein. Ce que c'est trop, mais ce n’est pas trop. Ouais voilà, mais j'aurais tendance à dire le bilan qu'on refait un directement à chaque séance puisque dès qu'on reçoit quelqu'un, on remet tout à zéro et on voit ce qui nous dit ce qu'il est capable de faire. Plus le premier bilan qu'on fait bien sûr beaucoup plus important au début des séances.

BJM

Avez-vous besoin que le patient ait effectué une épreuve d'effort ou que le médecin vous ait donné l'autorisation d'intensifier l'effort ?

MK3

J'aurais tendance à dire non, alors ça peut c'est toujours utile, hein ! En théorie, j'aurais tendance à dire qu'on est à peu près capable de le faire tout seul maintenant, sauf s'il y a une contre-indication

qu'on ne connaît pas, des choses comme ça au début, c'est toujours bien d'en savoir le plus possible puisque bien sûr, au bout d'un moment, on commence à connaître les patients et je vous dis, les problèmes sont plus connus, on a plus jalonné. Donc, au début ça peut être bien sûr très très intéressant parce que Ben il ne faut pas qu'il se repasse quoi que ce soit au cabinet ou pas, qu'on aille trop fort hein. Mais avec le temps et donc la connaissance du patient et de sa pathologie et de de sa manière de faire. J'aurais tendance à dire que non, mais au début, je pense que c'est un... voilà, c'est intéressant de ne pas faire de bêtises. Bien sûr, on est toujours sous l'aval du médecin qui c'est lui qui nous dit ce qu'on doit faire ou ce qu'on doit pas faire surtout, hein !

BJM

Évaluez-vous l'intensité des séances chez vos patients ? comment paramétrez-vous ces séances ?

MK3

Ben Ouais, toujours pareil en fonction d'eux, s'ils vont bien. Si au début de la séance ça va bien et puis pour une raison X ou y soit on est allé trop fort, soit eux sont allés trop fort, hein, ça peut arriver. Ou une douleur qui se réveille ou un essoufflement ou quelque chose comme ça. On adapte bien sûr la séance en fonction de ce qui se passe en face de nous. Donc, en le regardant alors parfois on met aussi sur tout ce qui est cardio, on a le pouls, donc la fréquence cardiaque. On peut prendre aussi l'oxymètre pour voir au niveau... Enfin, en fonction de voilà, on a quand même du matériel à côté de de bilan médical, entre guillemets, qui nous permettent de voir la saturation, les choses comme ça et qui peuvent être intéressants, surtout au début. Comme je vous dis.

BJM

Donc, quels outils d'entraînement utilisez-vous par comme l'ergomètre, le tapis roulant ou bien autre ?

MK3

Alors moi j'utilise l'ergomètre le tapis roulant puisqu'elle n'est pas capable de marcher, mais on marche dehors. Nous, on utilise aussi le vélo assis, puisqu'elle a une meilleure assise que sur le vélo tout court et donc l'ergomètre. Ouais, le vélo, le vélo à bras. Et puis après la marche à l'extérieur, puisque ça, c'est plutôt intéressant de la voir. Puis après des exercices avec le poids du corps de musculation, entre guillemets ou d'équilibre. Ou autre chose comme ça.

BJM

Adaptez-vous à l'intensité des exercices en fonction de l'État physique du patient ? Comment jugez-vous les séances en termes de fréquence, de durée et de capacité ?

MK3

Alors la fréquence, on la change très peu parce que c'est dans l'angle du temps, c'est plus facile, sauf s'il est malade. Si ainsi de suite hein, bien sûr, mais on change très peu le nombre de séances, sauf quand y a eu hospitalisation pour des raisons XY, et qu'il faut intensifier un petit peu, donc pendant quelques temps on augmente les séances. On ne les diminue pas parce que moi je n'en fais déjà pas énormément donc on essaie de ne pas trop diminuer. Et après on adapte Ben en fonction de sa fatigabilité, de son humeur. Et tout aussi, et de son envie aussi, ça un petit peu. Parfois, on peut être gentil et parfois un peu plus méchant sur certaines séances. On ne peut pas tout le temps faire travailler à 300%. Et il faut accepter, voilà parce que faut pas les mettre en échec aussi hein. C'est important que parfois ils réussissent des exercices. C'est important même dans la séance, de faire des exercices plus faciles pour récupérer ça mais aussi pour remotiver un petit peu parce que si tous les exercices sont difficiles, leur demandent énormément de concentration ou de choses comme ça. Ce n'est pas évident de recommencer après donc il vaut mieux des exercices là aussi. Dans l'intensité, on ne peut pas être tout le temps au maximum de l'intensité.

BJM

Leur faites-vous faire un test d'effort maximal comme le test de la marche de 6 Min ?

MK3
Ma patiente, Ben on fait tous les... à chaque fois on le fait mais ce n’est pas un test officiel mais oui c'est le test, voilà de on regarde à peu près en on a des habitudes aussi mais ça ce sont des tests faciles à reproduire donc oui.
BJM
Quels critères de surveillance utilisez-vous lorsque vous intensifiez les séances et est-ce que vous utilisez la fréquence cardiaque, la perception d’effort ou la saturation ?
MK3
Ah ! Tout. On a l’oxymètre, on a donc qui donne un petit peu tout. Nous, on a aussi un tensiomètre. Euh donc là on a comme ça la saturation, la fréquence cardiaque et la pression artérielle. En plus, Ben notre regard sur le patient qui peut transpirer, qui peut être plus blanc, plus rouge. Donc on a tous ces ouais, tous ces outils.
BJM
Donc, selon vous, quelles sont les connaissances théoriques, techniques et pratiques dont doit disposer le kiné pour intensifier les séances de rééducation des patients ?
MK3
Voilà déjà bien le regarder bien l'écouter. Hein, c'est important ! Et puis après Ben connaître un minimum sur les choses à pas dépasser la fréquence cardiaque en fonction de son âge. Enfin, il y a des petits critères quand même à dépasser et que le médecin peut nous donner, hein d'ailleurs au début.
BJM
Pensez-vous que vous avez été bien formé en formation initiale ? Est-ce qu’avez-vous eu besoin de formation complémentaire pour prendre, pour faire des exercices intenses chez les patients post AVC chronique ?
MK3
Je pense qu'en formation initiale, on est relativement bien formé, tout est relatif, c'est, c'est assez court hein ! Donc maintenant ça augmenté un petit peu avant. Donc je pense qu'on a quand même un bon bagage. Par contre, il n’est pas suffisant. Si on veut faire que ça, on est des généralistes hein. Donc si pour prendre des patients sans je ne vais pas dire sans gravité mais on a des capacités. Maintenant, je pense qu’il faut absolument approfondir tout. L’expérience sert à ça aussi hein, avec les patients, c'est un peu dommage, mais on grandit, on apprend, on fait peut-être des bêtises et on apprend des bêtises. On apprend aussi des chirurgiens ou des médecins qui vont nous dire des voilà, on apprend du coup. Il faut de toute manière se former, donc il faut essayer de former dans un maximum de choses. C'est vrai que la Neuro c'est compliqué donc il y a beaucoup, beaucoup de choses à voir, à savoir donc j'aurais tendance à vous dire oui, on est bien formé et j'aurais tendance à dire aussi qu’on n’est jamais assez bien formé donc. Il faut continuer à se former de toute manière, en fonction de ce qu'on a envie et des problèmes qu'on rencontre par rapport à chaque patient qu'on voit. Et Internet, il y a beaucoup de choses quand même pour apprendre, donc on peut approfondir les choses. Mais la formation initiale est quand même assez complète.
BJM
D'accord. Est-ce qu'il y a des freins concernant les patients à faire de l'exercice plus intense ?
MK3
Ça dépend, ça dépend des jours. Euh, moi, dans ce que j'ai dans le jeune aucun frein. Sur la dame un peu plus âgée, Euh… En la poussant un peu on arrive à faire beaucoup de choses, mais… Donc j’aurai tendance à dire que non. Non, si on leur explique bien, non.
BJM

Donc, ni la sévérité de leur handicap ou leur trouble cognitif ou leur fatigue ou leur motivation ne peut constituer un frein ?
MK3
Alors ça dépend ce qu'on appelle l'intensité, mais c'est l'intensité maximale pour eux. Enfin maximale sub-maximale pour eux. Bien sûr, on ne peut pas faire la même chose entre mon patient qui à 19 ans. Eh oui, je le fais courir, sauter partout, parce qu'il joue au foot, lui d'ailleurs hein ! Par exemple, c'est pas du tout la même chose que ma patiente qui pourtant pourrait mais je le referai jamais faire la même chose. Donc on adapte, on adapte à chaque patient, à chaque pathologie puisque même au même âge ils peuvent avoir des capacités différentes donc on adapte. Par contre, je pense qu'on ne peut-être pas à 100% de leur capacité d'intensité. On n'y sera jamais, mais en tout cas, on essaie de s'en rapprocher par les 100%, hein bien sûr, mais on va essayer d'être au maximum de ce qu'on peut, hein. Si, si je faisais les mêmes exercices à mon premier patient, enfin au jeune homme qu'à la dame, lui, on a une intensité qui est complètement différente. Par contre la dame, c'est sûr que je lui fais faire 3 fois le tour du quartier, je la vois plus pendant 15 jours parce qu'elle va être trop fatiguée donc. Mais par contre ça ne m'empêche pas de lui faire une fois le tour du cabinet où elle dit qu'elle est fatiguée, qu'elle ne peut plus rien faire, mais elle continue à faire quand même. Si vous voulez donc tout ça, c'est subjectif aussi. Par contre, faut adapter à chaque patient, ça c'est certain.
BJM
A votre avis, est-ce plus facile pour vous d'intensifier les séances de Kinésithérapie à la phase chronique de l'AVC ou en phase aiguë ? Et pourquoi ?
MK3
Alors vas-y faut toujours faire attention, il faut récupérer la confiance de la personne puisqu'elle a quand même eu un traumatisme important. Par contre, c'est la phase de récupération aussi, donc il ne faut pas le laisser trop trop rien faire hein. C'est là où on récupère au maximum. Donc ce ne sont pas les mêmes phases, ce n'est pas les mêmes choses. Par contre j'aurais tendance à dire qu'on essaye au maximum d'aller le plus possible pour chaque patient. En faisant attention bien sûr aux contre-indications à ce qu'il soit motivé et conscient de son ressenti. C'est vraiment lui qui va nous dire aussi s'il a une douleur dans la poitrine, s'il a une douleur quelque part, il faut qu'il nous dise et être à mon avis plus présent au début bien sûr, hein, sur les premiers temps sur la phase aiguë pour que le kiné soit vraiment présent quand il va faire un effort ou quelque chose comme ça, bien surveillé. Et la phase chronique, il est capable en théorie de se gérer bah tout seul, parce qu'on ne servirait à rien. Mais il est quand même capable de sentir les signes, de les gérer, de les comprendre et de dire si l'intensité ce jour-là est trop importante pour lui. Ça, c'est des choses très importantes à mon avis, à ce qu'ils connaissent parce que Ben on ne fait pas de l'activité que chez le Kiné, on en fait en dehors et c'est important que lui connaisse les signes d'alarme entre guillemets en dehors, puisque bah on ne sera pas tout le temps-là mais en phase aigüe, il faut lui apprendre, lui montrer et c'est important d'être présent.
BJM
Selon vous, l'intensification des séances est-elle importante dans la rééducation des patients ? Portables. Ici, on va se coller.
MK3
Oui, c'est important d'aller toujours de plus en plus. Il ne faut surtout pas perdre. Donc la rééducation, elle sert surtout à garder ce qu'on a. Mais je pense que on peut toujours aller au-dessus comme n'importe quelle autre personne, hein, avec une pathologie ou un problème de santé. Le but est à mon avis de se surpasser entre guillemets, de réussir à faire. Plus que ce qu'on pensait, entre guillemets, en faisant bien sûr attention à toutes les contre-indications, hein, toujours bien sûr, hein,

on ne va pas… mais on peut toujours trouver des ressources et améliorer les choses. Une personne qui n'était pas du tout sportive entre guillemets avant un AVC peut très bien le devenir beaucoup plus et même surpasser ce qu'il faisait avant. Donc on a parfois des récupérations qui sont des déclics et qui vous font travailler plus. Donc oui, oui, non, moi je pense qu'en phase chronique, on sait déjà ce qu'on a récupéré, mais c'est important.

BJM

D'après vous, doit-on l'intégrer à la pratique de routine et au traitement kinésithérapique ? Ou bien Pensez-vous que cela devrait faire l'objet de séances complémentaires ?

MK3

Non, je pense qu'on peut l'adapter pareil en fonction de chaque patient en fait. Voir ce qu'il en est. Mais c'est important de oui, je pense qu'il faut l'adapter pour qu'eux ça devienne vraiment, justement eux, une routine de le refaire à la maison, d'intensifier. Mais ça, ça dépend aussi de chaque patient qui ne sera pas obligatoirement motivé ou quand ma patiente elle n'est pas motivée. Je crois qu'on pourrait… Je ne sais pas encore ce qu'il faut trouver pour la faire courir, mais on ne serait pas capable de courir, mais… Mais ouais, il faut vraiment chercher ce qui va aider les gens à faire plus. Et c'est important.

BJM

Pensez-vous qu'il est de votre ressort de faire faire des activités intenses à vos patients ? Et habituellement, qui prescrit les exercices intenses ?

MK3

Alors oui, je pense que c'est notre sort de le faire, alors ça dépend ce qu'on appelle intensité hein. Moi je fais pas non plus faire d'épreuves d'efforts hein, on n'est pas sur cette intensité-là qui est beaucoup, beaucoup plus importante. Nous, on est vraiment pour améliorer les choses, mais pas obligatoirement de l'intensité comme une épreuve d'effort ou là ils sont contrôlés et ainsi de suite. Nous, on est bien en deçà de l'épreuve d'efforts, hein. Par exemple, si ça l'est, c'est l'intensité maximale, nous, on est vraiment bien en dessous. On est sur… Donc tout dépend d'où, d'où on place, l'intensité maximale. Et donc après, si on veut être sur une intensité maximale à la suite d'un AVC, je pense qu'il faut vraiment que là il y a un médecin qui soit tout prêt et une équipe médicale puisque hélas ça peut-il peut y avoir des problèmes là. Dans un cabinet libéral, sans médecin à côté, sans cardio à côté, une épreuve d'effort, elle n'est pas, à mon avis, elle est pas obligatoirement recommandée. En tout cas moi je ne le ferai pas. Je n'ai pas assez de compétences à mon avis.

BJM

D'accord et en dehors des séances au cabinet prescrivez-vous des exercices intenses que le patient doit faire chez lui ?

MK3

Pas obligatoirement intense plutôt sûr du Euh…Tout est bon, hein, mais plutôt des choses répétitives, enfin plus pour travailler sur le, le cardio pour travailler cette… L'intensité qu'on va pouvoir mettre après, donc pas obligatoirement intensif, non pas en dehors.

BJM

Avez-vous peur des conséquences qu'il pourrait y avoir chez ces patients si vous intensifiez les séances ? Lesquelles ?

MK3

Alors moi, personnellement non, puisque c'est je…Vous dis c'est très loin. Je suis pas du tout en phase aiguë donc moi non. Par contre, en phase aiguë, il faut Ben connaître le mieux possible le patient pour éviter tout ça, dans le bilan, dans tout ce qu'on va pouvoir récupérer du médecin, tout ce qui va pouvoir nous dire. Mais autrement, est ce que j'ai peur ? Non ! Après, pas spécialement peur, mais il faut vraiment qu'eux se connaissent et que, nous on les connaisse.

BJM
D'accord, donc, c'est la fin de l'entretien, je vous remercie pour ce temps passé avec vous, souhaitez-vous ajouter une dernière remarque concernant le thème évoqué aujourd'hui ?
MK3
Non, je pense que. J'ai dit plein de choses déjà.
BJM
Avez-vous des questions à me poser ?
MK3
Je pense qu'on m'a bien expliqué. Voilà, c'était dans le cadre des études, donc c'est intéressant de s'occuper de la Kinésithérapie donc non je trouve ça très bien.
BJM
D'accord, bon, je vais éteindre l'appareil d'enregistrement.

Retranscription de l'Entretien 4 (Enquête 4, MK4)
Entretien réalisé le 6 avril 2023
Durée 26 minutes 54 secondes
Présentateurs : Bénédicte JEAN MICHEL(BJM), Masseur Kinésithérapeute (MK4)
audio1727168693.m4a
Transcription
BJM
Donc pour débuter cet entretien puis-je s'il vous plait vous demander de vous présenter brièvement, votre âge, votre formation. Si vous avez suivi des formations complémentaires, votre expérience professionnelle.
MK4
Alors moi c'est, je m'appelle X, j'ai 30 ans, je suis diplômé depuis Attendez, faut que je réfléchisse. Depuis 2016, depuis 2016 je suis diplômé donc ça fait 7 ans maintenant. Comme formation complémentaire ? Alors, je suis diplômé d'un institut parisien qui s'appelle l'institut Danhier. J'ai comme j'ai passé comme formation complémentaire. J'ai un diplôme universitaire dans… de la SFMKS donc, un certificat d'études aux pratiques sportives. J'ai passé comme formation complémentaire. Après plein de formations à droite à gauche. Des formations en communication. Des formations en distanciel sur la douleur, sur la formation d'épaules. Chez Ben, ce fut une formation sur la ligne du coureur, l'Institut McKenzie. Qu'est-ce que j'ai fait d'autre, j'en ai fait, j'en ai fait d'autres, mais j'ai plus forcément trop en tête. Voilà.
BJM
Depuis combien de temps exercez-vous en libéral ?
MK4
Depuis, depuis mon diplôme. Alors donc ça va faire 7 ans, bientôt 8.
BJM
Donc, sur 100 patients, combien de patients post AVC Avec vous dans votre patientèle ?
MK4
En phase chronique ou phase aiguë ?
BJM
Phase chronique.

MK4
Sur 100 patients.... Ouais peut-être, mais je dirais... Je dirais 1/5, 1/10, peut-être même pas 1/5 je dirais 1/10 ouais.
BJM
D'accord combien de fois par semaine et combien de temps ?
MK4
Je les vois généralement 2 à 3 fois par semaine sur des séances d'une heure.
BJM
En groupe ou bien en individuel ?
MK4
En groupe.
BJM
Les voyez-vous sur une durée limitée de séances ou bien en continue ?
MK4
Je les vois en continue, ouais, je les vois en continue.
BJM
Donc, à la suite des résultats préliminaires de l'étude observationnelle que vous avez participé, nous avons manqué que l'intensité des séances différait de patients à l'autre. Quel regard portez-vous sur l'intensité des séances de kinésithérapie ? Et quelle importance avez-vous dans la mise en place de l'intensité des séances ?
MK4
Quelle importance, moi j'accorde à mettre de l'intensité dans mes séances ?
MK4
Et Ben, j'accorde de l'importance à mettre de l'intensité en fait. Je n'apporte pas tant d'importance à mettre de l'intensité et j'apporte de l'importance à les mettre en difficulté. A les mettre en difficulté dans des activités qu'ils ont à faire au quotidien. Et effectivement l'importance que je mets, c'est que ça les fatigue, ça les fatigue parce qu'ils ne sont pas toute la journée au fauteuil, tout le temps. Ils ont besoin de faire des transferts, ils ont besoin de faire des tâches qui vont être plus coûteuses que d'autres. Donc j'essaie de de me dire que… j'essaie de faire en sorte de les mettre dans des situations de fatigabilité, pour que ce soit le plus reproductible de ce qu'ils ont besoin de faire à la maison.
BJM
Donc, connaissez-vous des recommandations en termes d'intensité d'exercice pour cette population ?
MK4
Pour la population AVC il me semble que c'est 180 Min d'activité modérée. 180 Min d'activité modérée par semaine.
BJM
À votre avis, la séance que nous avons observée avec votre patient était-elle, intense ou pas du tout ?
MK4
Elle est entre...elle est intense. Elle l'est généralement. Ils ressortent assez fatigués. Enfin, après, ça dépend desquelles, parce que ce n'est pas tous les cas. Il y en a qui ont des freins, qui font que l'on ne peut pas monter en intensité. Enfin, on n'arrive pas à monter aussi haut en intensité avec certains qu'avec d'autres, mais généralement on est sur moyennement intense à intense.
BJM
Moi donc pourquoi elle était moyennement intense ?

MK4
Ben parce qu’il y a des fois… Vous parlez des séances en général ou le jour où Stéphanie a fait l'étude.
BJM
Le jour.
MK4
Le jour où Stéphanie a fait l'étude ? Non, on était sur… on était sur intense.
BJM
Et d'accord avec vous, pourquoi ?
MK4
De mémoire, hein ? Parce que.
MK4
Je me rappelle plus exactement, non ? Plus c'est il y. A quand même un an et demi, je crois maintenant 2 ans donc je me rappelle plus exactement, mais je crois que c'était intense.
BJM
D'accord, vous avez parlé des freins par rapport aux patients lorsque vous mettiez en place des exercices intenses. Quels sont les points que vous avez observés ?
MK4
Des restrictions de mobilités, restriction de mobilité des personnes qui n'arrivent pas à se verticaliser. Récemment, j'avais un patient qui avait été au fauteuil pendant 3 ans sans kiné. Donc bah moi quand il est arrivé en kiné tout simplement, il avait des flessums de genoux tellement importants qu’il n’arrivait pas à se verticaliser correctement. Des flessums et des équins au niveau de cheville qui fait que on n'arrivait pas à se verticaliser, ça c'est un frein. Parce qu’on arrive quand même à avoir de l’intensité. Et on arrive quand même à avoir un peu d'intensité, mais on va juste lui faire monter le cardio. Il va se verticaliser mais moi j'ai remarqué qu’au niveau cardiaque on ne le fait pas monter extrêmement fort en intensité. Ça, ça va être un frein. Les limitations articulaires ou musculaires, les limitations cognitives aussi, hein ! Limitation cognitive, ça peut être un frein à la rééducation, s’il n’y a pas une bonne compréhension de de la consigne ou que le patient va être en permanence en danger. Si on le verticalise ou qu'on le fait marcher. Ça va être un frein également. Puis, qu'est-ce que je peux avoir d'autre en tête ? C'est tout. Ouais c'est. Tout, enfin moi, de ce que je vois. Je, je n’en ai pas d'autres en tête là pour le moment. Mais c'est ce que je vois à peu près.
BJM
Pensez-vous que l'intensité des séances a une réelle importance dans la rééducation des patients ?
MK4
Ah Ben absolument ça, oui, j'en suis… J'en suis convaincu. Et puis les résultats de Stéphanie nous montre aussi un petit peu qu’il y a une réelle importance parce que des fois il y a une inadéquation entre ce qu'on trouve à la maison et en séance. Et oui, c'est sûr, c'est sûr qu'il y a une importance parce qu’Il y avait les bénéfices de l'activité physique ont plus de preuve à faire. On sait que c'est important. On sait que…
MK4
Ça stimule énormément de choses. Et plus on va monter en intensité, plus on va activer tout un tas de systèmes. Et pour ces personnes-là en plus qui ont des restrictions de mobilité, en fait, ils vont avoir des efforts courts et intenses à faire chez eux. Quand ils ont besoin de faire un transfert, c'est un effort court mais intense pour eux et donc plus on va être reproductible en séance, plus ça va être, plus ça va leur être bénéfique à la maison.
BJM

Dans votre pratique, en dehors des séances au cabinet, vous prescrivez des exercices intenses à domicile à vos patients ?
MK4
Non non, je ne prescris pas des exercices intenses à la maison parce que généralement. Enfin ça dépend pour qui ça dépend pour quel patient mais là les deux que j'ai enfin. Mais les patients que j'ai en tête ils ne peuvent pas faire des exercices intenses à la maison parce qu'ils n'ont pas le matériel, ou alors parce qu'ils vont se mettre en danger. Là, j'ai une patiente qui se verticalise bien au cabinet tout seul. À la maison, si elle tombe, elle est toute seule chez elle. Si elle tombe, c'est compliqué.
BJM
Donc, selon vous, quel est l'environnement technique matériel et professionnel nécessaire à la mise en œuvre d'intensification des séances de rééducation des patients post AVC en phase chronique ?
MK4
Redites-moi la question.
BJM
Quel est l'environnement technique, matériel et professionnel nécessaire à la mise en place des intensifications des chances de rééducations ces patients ?
MK4
A la maison ou au cabinet ?
BJM
Oui au cabinet.
MK4
Au cabinet le matériel, le matériel nécessaire, je dirais des barres parallèles, des barres parallèles, un cyclo ergomètre que ce soit à bras ou à jambes.

MK4
Un plan de Bobatt. Et puis après je n'ai pas l'impression qu'il faille beaucoup. Enfin, qui ait besoin d'énormément de matériel. Peut-être que je me trompe, mais je n'ai pas l'impression.
BJM
D'après vous, quels sont les freins ou obstacles environnementaux à sa pratique et quels sont les facteurs facilitants ?
MK4
Quels sont les freins et les facteurs facilitants ? À la pratique, à sa pratique ?
BJM
Oui, d'un point de vue environnementale, au niveau du cadre de l'espace.
MK4
A la maison ou au cabinet ?
BJM
Au cabinet.
MK4
Les freins, les freins, ça va être un manque de matériel, un manque d'espace. Euh, manque de temps parce que nous on fait des séances de groupe, alors c'est plus facile de les prendre 1h et de et de. Les faire travailler. Mais un kiné qui prendrait un patient que 30 Min, ça va peut-être être plus compliqué de faire des séances où l'intensité va monter. Le manque d'espace, hein ? Et facteur facilitant Ben, c'est. Les obstacles aussi. Je dirais que c'est peut-être une peur, la peur d'aller chercher, d'aller faire monter aussi l'intensité cardiaque chez ces personnes-là. Mes connaissances peut-être de la pathologie. Puis voilà.

BJM
Donc, par rapport au temps. Combien de temps pensez-vous qu'il faudrait pour mettre en place ce genre d'exercice ?
MK4
Pour les professionnels ? Pour tous les professionnels ?
BJM
Oui
MK4
Parce que nous, on le. Fait enfin, j'ai l'impression qu'on le fait déjà au cabinet. Après, dans la pratique professionnelle de tous les autres kinés, je dirais qu'il faudrait 1h. Il faudrait bien 1h, peut-être même plus après 1h00, c'est bien généralement 1h00. Les patients ont-ils sortent bien cramés donc 1h ça reste bien 30 Min je. Pense que c'est un peu court. Honnêtement, c'est trop court.

BJM
Donc 1h c'est trop.
MK4
Non, 1h c'est. Bien 30 Min ce n'est pas assez. Et 1h30, ça serait peut-être trop.

MK4
Mais bon. Il y a tellement de choses à faire. Aussi parce que là on parle d'activité physique mais dans des séances il va y avoir aussi de la mobilisation passive. Il va y avoir, il va y avoir plein d'autres choses à faire. Aussi, donc 1h30 en fait ça ne serait même pas forcément trop, puisque on pourrait toujours travailler sur d'autres choses. On peut travailler de la, des exercices de prévention, d'autres exercices qui ne sont pas de l'activité physique, mais qui vont être bénéfiques pour eux. 1h et demie c'est carrément faisable. 1h30 aussi.
BJM
Et d'après vous, doit-on l'intégrer à la pratique de routine et aux traitements kinésithérapique ?
MK4
Intégrer quoi ?
BJM
L'intensité des séances
MKA
Ouais, complètement
BJM
Par rapport aux intensités. Comment sélectionnez-vous les patients à qui vous faites faire de l'exercice plus intense ?
MK4
Ce qui ont encore ceux qui arrivent à se verticaliser seuls et ce qui ont encore la capacité de marche. Et encore même pas parce qu'avec un vélo à bras on arrive à quelqu'un qui… Je dirais que…. C'est, ce n'est même pas ça, c'est que… Ça va être le cognitif au final. Ça va être le cognitif quand le cognitif alors… Il faudrait les faire passer des tests, il faut les faire passer des tests cognitifs comme un mini mental State, éventuellement pour déterminer la faculté cognitive qui vont voir si les patients ont encore des bonnes capacités cognitives. Quand ils ont de bonnes capacités cognitives, c'est facile de leur faire comprendre qu'il faut envoyer sur un exercice même s'ils arrivent plus trop à se verticaliser. On prend un vélo à bras, on prend un élastique, on prend des poids, on arrive à leur faire faire des choses, si tenté qu'ils comprennent bien qu'il faut y aller sur l'exercice.
BJM

Avez-vous besoin que le patient ait effectué une épreuve d'effort ou que le médecin vous ait donné l'autorisation d'intensifier l'effort ?
MK4
Non, alors je n'attends pas que le médecin me donne l'autorisation. Du moment où il m'envoie un patient kiné, c'est que c'est que j'imagine que toutes les précautions ont été prises avant.
BJM
Non, vous pensez que c'est du ressort du kiné de prescrire une intensification des séances ?
MK4
Non, ce n'est pas à moi de le prescrire. Le médecin quand il m'envoie un patient, quand il envoie un patient, j'imagine qu'il a pris toutes les précautions avant pour être sûr qu'Il n'y a pas de risque et non, ce n'est pas forcément au Kiné de prendre cette initiative-là. Bien qu'on pourrait largement le faire. Mais il faudrait-il faudrait. Il faudrait mettre des barrières ça.
BJM
Quand vous dites des barrières, vous entendez par quoi ?
MK4
Des barrières, s'est s'assurer qu'il n'y a pas de risque pour le patient, voilà. D'être vigilant sur l'attention, sur l'intensité cardiaque. Voire réaliser des petits tests d'effort. Voilà sur que ces patients-là n'ont pas de risque à pratiquer ça.
BJM
Donc, habituellement, quels critères de Surveillance utilisez-vous lorsque vous intensifier les séances ?
MK4
Critères ? je n'en utilise pas beaucoup, malheureusement, je n'en utilise pas beaucoup. Je vais vous dire que mon marqueur, ça va être une échelle de Borg, ça va être une dyspnée d'effort hein !
BJM
Donc évaluez-vous l'intensité des séances chez vos patients ?
MK4
Je ne l'évalue pas, je n'ai pas le temps. Honnêtement, je n'ai pas le temps, il faudrait… c'est ça ? C'est de l'étude, Hein, c'est de l'étude, mais y a qu'à voir combien de temps ça prend à faire une étude pour un patient. Ça prend une heure et demie. Faut faire passer des tests, faut après faut analyser les résultats non. Honnêtement, ça fait partie du domaine de la recherche et en cabinet, malheureusement, on n'a pas le temps. Après, il y a peut-être un cardiofréquencemètre, ça ne prend pas beaucoup de temps à installer ou quoi que ce soit, mais non, on a plein, on a d'autres patients en même temps et malheureusement on n'a pas le temps de consacrer.
BJM
Adaptez-vous l'intensité des exercices en fonction de l'état du patient ?
MK4
Bien sûr. Ouais bien sûr. Ce n'est pas les, ce ne sont pas les mêmes séances pour tous les patients, donc on adapte pour chaque patient.
BJM
Comment ajustez-vous les séances en matière de durée, d'intensité et de fréquence ?
MK4
Comment moi je gère ça ?
BJM
Comment ajustez-vous les séances en matière de durée, d'intensité et de fréquence ?
MK4

Bah ce que je vous disais 3 fois par semaine une heure par séance. Entre 2 et 3 fois par semaine,1h par séance.

BJM

Donc au cours de ces séances, est-ce que vous les faites faire un test d'effort maximal comme le test de la marche de 6 minutes ?

MK4

Non, non. Non parce qu'un test de marche déjà, c'est, c'est enfin… Test de la marche… La plupart de mes patients n'arrivent pas à faire un test de marche de 6 minutes ça hein ! Ils ne tiennent pas sur un tapis, hein ! Donc non, je ne peux pas. Après y a d'autres tests qui existent, on peut bien faire un test de 10 M de marche, ce genre de choses-là, mais ça ne va pas être un test d'effort. Enfin, pour moi ça ne va pas l'être. Peut-être que je me trompe, mais…

BJM

Donc, selon vous, quels sont les connaissances théoriques, techniques et pratiques dont doit disposer le kinésithérapeute pour intensifier les séances et les patients post AVC en phase chronique ?

MK4

Pour intensifier les séances. Je dirais que on travaille un peu, on travaille un peu au feeling et c'est sans doute une erreur de notre part. Faudrait arriver à avoir un test d'effort du patient. Il faudrait qu'on sache juste jusqu'où il est prêt à aller lui, enfin, jusqu'où il arrive à aller et que nous, on puisse ensuite ajuster ça par rapport. Si le patient est arrivé avec un test d'effort en disant au bout de telle intensité c'est trop dur pour lui. Déjà, on a des curseurs, on a des curseurs. Où on sait où on peut aller, où on peut aller. Donc ce serait un bon prérequis.

BJM

Pensez-vous que vous avez été bien formé en formation initiale ou bien avez-vous besoin de formations complémentaires ?

MK4

Alors j'ai, j'avais une super prof de Neuro, qui était la cadre de santé de l'hôpital de Garches, en région parisienne, qui s'occupe exclusivement des cérébrolésés Donc c'était une super prof, une super prof qui nous a extrêmement bien formée. Je pense en neuro après on ne nous a pas assez former sur les dépenses énergétiques de ces populations-là. On nous a extrêmement bien formés sur comment les faire travailler sur ce qu'il fallait travailler, sur les bilans, sur les évaluations qu'il fallait faire avec ce genre-là. Mais pas sur les bilans de l'activité physique.

BJM

Donc, il vous manquait une formation supplémentaire ?

MK4

Oui, oui, oui, il faut enfin. Il me manque, oui, enfin. Il faudrait l'intégrer. Un peu plus à la formation initiale. Après je pense qu'on arrive très bien à se débrouiller sans aller chercher. Notre formation en globale fait qu'on peut arriver à intégrer d'autres spécialités, de la neuro aussi.

BJM

À votre avis, c'est plus facile pour vous d'intensifier les séances de kinésithérapie à la phase chronique de l'AVC qu'en phase aiguë ?

MK4

C'est une très bonne question, c'est une très bonne question. Je dirais que c'est plus facile en face chronique puisqu'en phase aiguë, il va y avoir tout un tas de choses à travailler. Il va y avoir tout un tas de choses à travailler. Alors ça dépend où est-ce qu'ils sont. S'ils sont en centre, s'ils sont en centre, ça va être facile d'intensifier les séances, d'aller augmenter l'intensité physique. S'ils sont en libéral, ça va être compliqué. Parce qu'en en phase aiguë, il va y avoir tout un tas de choses à

travailler. Il va y avoir de l'équilibre, de la mobilisation, c'est la phase vraiment où il faut y aller. Et si on ne fait que de l'activité physique, je pense qu'on va être aux dépens d'autres systèmes que ce soit particulièrement musculaire, que ce soit du membre suivi que ce soit cognitif. Et je pense que si on fait que de l'activité physique et qu'on n'intensifie enfin pas faire que l'activité physique, mais si on intensifie à cette phase-là, j'ai peur que ce soit au dépend d'autres facteurs. Donc je dirais que c'est peut-être plus facile en chronique. Mais encore une fois, ça dépend si on est en libéral ou si on est en plutôt en en structure ou en centre de rééducation.
MK4
Je ne vous aide pas du tout dans ma réponse, mais…
BJM
Donc, et si on est en phase chronique et en cabinet libéral, est-ce que c'est plus facile ?
MK4
Je dirais que c'est plus facile, ouais.
BJM
Avez-vous peur des conséquences qu'il pourrait y avoir chez ces patients si vous intensifiez les séances ?
MK4
Non, absolument pas.
BJM
Donc, ni les troubles cognitifs et troubles cardiaques ne sont pas un obstacle ?
MK4
Bah les troubles, les troubles cardiaques, j'imagine que de toute façon on s'en s'est, on est censé avoir des barrières par rapport à ça, hein ! Du moment où on a une prescription médicale, c'est que le médecin a mis ces barrières-là. Donc le patient est capable de les supporter. Bien sûr, on ne va pas les faire faire un marathon à notre patient non plus.
MK4
Il est capable de supporter ça et puis enfin, ce n'est pas de l'activité physique modérée ou intense j'imagine qu'ils vont lui déclencher. Ou alors, c'est qu'il y a un problème qui n'est pas bien géré. Si, si mon activité physique lui déclenche des soucis cardiaques, c'est qu'il y a un souci cardiaque sous-jacent dont le médecin ne s'est pas préoccupé. Donc moi, je pars du principe que non je n'ai pas de risque a augmenté ça. Après, si je sens que ça ne va pas bien de façon je prendrai la tension. Je prends le pouls voilà, je. On ne va pas faire n'importe quoi, non plus. Si on sent que le patient n'est pas bien, mais je n'ai pas de de crainte ni de peur par rapport à ça.
BJM
D'accord, c'est la fin de l'entretien, je vous remercie d'avoir participé.
MK4
Bah c'est avec plaisir.
BJM
Souhaitez-vous ajouter une dernière remarque concernant le thème ?
MK4
Non, c'est tout bon pour moi.
BJM
Avez-vous des questions à me poser ?
MK4
Non, non, j'espère vous avoir été utile.

Retranscription Entretien 5 (enquête 5, MK5)
Entretien réalisé le 06 AVRIL 2023
Durée 24 minutes 26 secondes
Présentateurs : Bénédicte JEAN MICHEL(BJM), Masseur Kinésithérapeute (MK5)

Fichier audio
audio1168150210.m4a
Transcription

BJM
Donc pour des.
Présentateur 1
J'ai compris.

BJM
Pour débuter cet entretien, puis-je s'il vous plaît, vous demandez de vous présenter brièvement, votre âge, votre expérience professionnelle, votre formation initiale et si vous avez eu des formations complémentaires ?
MK5
Alors ? Eh Ben je m'appelle X je suis kiné depuis... diplômée en 2004, j'ai fait mes études à Paris. Après j'ai fait un petit peu de remplacement, un petit peu d'assistanat et puis après je me suis installé avec mon épouse pour créer un cabinet en Dordogne. Voilà, aujourd'hui on est plusieurs associés au sein de ce même cabinet. Voilà en formation : j'ai donc mon diplôme d'État de Kinésithérapeute, j'ai un diplôme d'ostéopathe, j'ai un DIU de réhabilitation cardio-respiratoire et puis après en termes de ça, c'est pour les diplômes. Et en termes de formation, et Ben j'ai des formations diverses et variées sur les TMS, les... tout ce qui est réhabilitation respiratoire aussi. Kinés, kinésithérapie apparemment respiratoire. Voilà, voilà, il y a pas mal de choses. Ben voilà, je. Pense que j'ai fait le tour là actuellement bah. J'interviens aussi dans des structures type SFR ou EHPAD. En l'occurrence la SFR et puis bah ma foi, voilà. Je pense que j'ai fait le tour. À peu près.

BJM : Oui, depuis combien de temps exercez-vous en libéral ?
MK5
Depuis 2004 donc ça fait 18, 19 ans. Ça ne nous rajeunit pas tout ça.

BJM : Sur 100 patients, combien de patients post AVC avez-vous dans votre patientèle ?
MK5
Bonne question. Donc le pourcentage que j'ai de patients alors AVC ? Dire que je réfléchis par rapport, ce n'est pas facile. Oui, là j'ai peut-être, euh…10 à 15%, on va dire.

BJM : Donc, combien de combien de fois par semaine les voyez-vous ?
MK5
Ça dépend, mais en règle générale, 2 fois.

BJM : En groupe ou en individuel ?
MK5
En groupe.

BJM : Les voyez-vous sur une durée limitée ou en continu ?
MK5
Comment ça ?

BJM : Est-ce que les séances sont limitées ou en continues ?
MK5
Est-ce qu'il y a des temps ?

BJM : Est-ce que ça se suit dans le temps, les séances ? est-ce qu'il y a un nombre limité de séance ?
MK5
Non, non, non, ils sont. Ah oui. D'accord, non, il n'y a pas de nombre limité de séance, non. Donc c'est continue. Ouais c'est ce n'est pas… ce sont des phases, c'est oui effectivement phase chronique, ce sont des séances qui sont continues oui.

BJM : D'accord à la suite des résultats préliminaires de l'étude observationnelle que vous avez participé, nous avons remarqué que les séances différaient d'un patient à l'autre. Quel regard portez-vous sur l'intensité des séances de rééducation chez les patients post AVC en phase chronique ?
MK5
Bah le regard que je porte là-dessus, c'est. Je pense que ce n'est pas forcément facile de le faire chez tous les patients pour diverses raisons, c'est parfois ça peut être des doutes sur la… Comment dire ? La capacité du patient à le faire après. Sinon, qu'est-ce que c'est mon regard sur les ? Je ne sais pas ce qu'il faut. Je peux répondre à ça. Et c'est quoi ? Mon regard sur l'activité physique au sein d'une séance ?

BJM : Par exemple, quelle importance accordez-vous à l'intensité, dans la mise en place de ces séances ?
MK5
Ah bah ouais, d'accord ! Ouais Bah si, c'est très important ! Ouais, il y en a dans chaque mois avec chaque patient, il y a forcément l'activité physique d'intensité, on va dire modérée à forte. En tout cas, c'est ce que j'essaie de mettre en place. Voilà, et ça fait partie du corps de la séance. Il n'y a pas que ça, mais ça en fait partie. Dans chaque séance.

BJM : D'accord qu'entendez-vous par intensité des séances chez les patients post AVC en phase chronique ?
MK5
En phase chronique, intensité des séances et Ben je ne sais pas. Bon ouais je ne sais pas. Ce que j'entends par intensité ? Bah l'intensité de la séance, je ne sais pas. Moi le je ne vois pas trop. Enfin, le mot veut tout dire.

BJM : Comment le définiriez-vous par exemple intensité des séances ?
MK5
Eh Ben. Bah par de l'activité. Enfin, par des par des comment… Par d'éboulement actifs, voilà donc. Par des mouvements actifs comme les gens demandent aux patients d'être actifs, de faire je ne sais pas moi. Vous voulez des exemples ? Du tapis de marche, du vélo, ce genre de choses. Voilà après, ça peut être de la marque rapide de ce genre de choses. Ouais.

BJM : Connaissez-vous des recommandations en termes d'intensité d'exercice pour cette population ?
MK5
Oui, un petit peu, ouais.

BJM : Donc, à votre avis, la séance que nous avons observée avec votre patient était-elle, intense ou pas du tout ?
MK5
Avec tout ? Bah ça dépend du patient.

BJM : Par exemple, le patient que Stéphanie avait vu avec vous.
MK5
Elle en a vu plusieurs. Moi, j'en ai eu plus. Elle, elle a pris plusieurs patients, Stéphanie donc du coup. J'en choisis un. Donc ça dépend des patients, mais il y en a certains qui avaient effectivement une activité d'intensité modérée. Au cours de la séance de Kiné il y en a d'autres qui avait moins au cours de la séance parce que douleur, parce que blême. Parce que voilà, donc ça dépend des, ça dépend des patients. Ça dépend surtout de leur tempérament et de ce qu'ils peuvent faire aussi. C'est surtout leur motivation.

BJM : Vous adaptez les séances en fonction du patient ?
MK5
Oui bah oui. Ouais, je ne fais pas tous les mêmes exercices à tous les patients, on adapte selon ce qu'ils peuvent faire, c'est selon les restrictions de mobilité qu'ils ont leur importance. Enfin, il y a des traitements médicamenteux aussi qui font que bah ils vont être plus dyspnéiques ou des choses comme ça. Donc du coup Ben on adapte la séance aux patients et sa capacité.

BJM : Selon vous, quel est l'environnement technique et matériel à la mise en place d'intensification des sciences de rééducation des patients post AVC en phase chronique ?
MK5
En terme donc de matériel, on a tapis de marche, vélo, vélo type motomètre, donc, des vélos semi assistés ou assistés, des vélos semi assis, des barres parallèles. Voilà essentiellement, pour ce qui est de l'intensité de l'activité physique. Ouais, je pense que c'est pas mal comme matériel ça. Les escaliers aussi.

BJM : Donc, selon vous, quels sont les freins ou obstacles environnementaux à sa pratique et quels sont les facteurs facilitants ?
MK5
Les freins, ça va être. Bah justement, l'équipement, le manque d'équipement pour certains. Les freins, ça va être aussi le manque de... comme la peur de faire. La peur de faire mal aux patients. S'il a des problèmes cardiaques ou des choses comme ça. Voilà un petit peu, la peur de l'intensité de l'exercice. Et après, c'était quoi le l'autre parti ?

BJM : Les facteurs facilitants ?
MK5
Les facteurs facilitant Ben c'est la motivation du patient, c'est le fait d'être en groupe. C'est l'environnement, c'est-à-dire le matériel. Voilà essentiellement, ouais.

BJM
Par rapport à l'espace, est-ce que le manque d'espace disponible et accessible est-il une limite ?
MK5
Oui, je pense oui. Dans les cabinets, vous voulez dire pas chez moi en tout cas, mais dans les cabinets, la plupart du temps, je pense que oui, ça peut être un frein.

BJM
Qu'est-ce qu'il faudrait à un kiné en termes d'espace pour pouvoir mettre en place ce genre d'exercices ?
MK5
Bah au moins un… Bah euh en termes d'espace on va dire. Je ne sais pas, un plateau technique, voilà. Et après ça, si on considère qu'on travaille en groupe, environ 50 m² par exemple, ça me paraît pas mal. Il n'y a peut-être pas besoin d'autant, mais tout dépend si on travaille en groupe ou en individuel. Et sinon, après un espace suffisant pour pouvoir mettre comme je vous disais un tapis de marche, un vélo ou 2 vélos. Un vélo assis par exemple, un cyclo ergomètre. Voilà qu'on puisse avoir suffisamment d'espace. Donc ouais en groupe on va dire 50 mètres carrés. Et si c'est individuel, 20 mètres carré, ça peut suffire quoi ! Mais bon ça peut faire. Enfin, je pense qu'en groupe, c'est mieux pour les raisons que j'ai dit tout à l'heure et donc 50 m², ça me paraît bien ouais comme plateau technique.

BJM : Donc, en termes, le temps disponible dans une seule séance de traitement de kinésithérapie constitue elle un obstacle ?
MK5
Alors ! C'est pour ce que j'en pense globalement ?

BJM : Oui, oui.
MK5
Bah, si on s'en réfère au texte, on va dire que bah oui c'est juste. Parce que c'est 30 Min, mais la plupart des cabinets, quand on fait des séances en groupe, c'est plutôt de l'ordre d'une heure donc. 1h, ça me paraît ça me paraît le minimum en tout cas ouais pour pouvoir faire... En sachant qu'il y a pas mal de temps de repos pour le patient, etc. etc. Donc je pense qu'un... comment on va dire qu'un créneau d'une heure et demie, ce serait, je pense peut-être l'idéal, ouais.

BJM : Comment sélectionnez-vous les patients à qui vous faites faire de l'exercice plus intense ?
MK5
Eh Ben je regarde leur capacité à pouvoir le faire. Je m'attache essentiellement à l'échelle de Borg. Puis après au ressenti du patient. La plupart du temps, c'est l'échelle de Borg qui va m'indiquer, si je suis au niveau d'intensité que je souhaite pour le patient, sur l'exercice.

BJM : Donc avez-vous besoin que le patient ait effectué une épreuve d'effort ?
MK5
Non.

BJM : Ou que le médecin vous ait donné l'autorisation d'intensifier l'effort ?
MK5
Non.

BJM : Evaluez-vous l'intensité des séances chez vos patients ?
MK5
Comment ça ?

BJM : Par exemple, est-ce que vous paramétrez ces séances en leur faisant des bilans personnalisés ?
MK5
Oui, si je donne un exemple sur un tapis de marche ou je vais faire faire par exemple 2 Min à un patient qui ne peut pas faire plus, et c'est déjà compliqué pour lui. J'ai toujours la même vitesse de marche et je vois la distance qu'il va effectuer. Voilà par exemple. Donc ça ce sont des critères que je retiens donc ce sont des bilans, Oui ! Ou des évaluations comme je viens de dire, individuelles et j'ai des données. Comme ça, par exemple sur le tapis de marche.

BJM : Habituellement, quels outils d'entraînement utilisez-vous (des ergomètres, tapis roulant ou pieds libres ou autre chose) ?
MK5
Ah oui. Bah oui, du coup, tout ce que j'ai dit. Tapis de marche, vélo assis, Moto Med, cyclo ergomètre voilà. Ouais, tout ça, j'utilise.

BJM : Quels critères de surveillance utilisez-vous lorsque vous intensifiez les séances, est-ce que vous utilisez la fréquence cardiaque, tensiomètre, saturation, perception d'effort ?
MK5
C'est ça. Perception d'effort essentiellement, c'est vraiment ce que j'utilise le plus. Et après, la perception d'effort vu par le patient et ce que moi je vois aussi. C'est à dire que je vois aussi, il y a des signes. Voilà à son visage par exemple. Encore une fois, la dyspnée voilà ce genre de choses. Et tout donc, on va dire c'est le ressenti du patient et mes connaissances à moi en termes de signes cliniques, voilà qui vont faire…Que je vais pouvoir évaluer le niveau d'intensité.

BJM : Donc, selon vous, quelles sont les connaissances théoriques dont doit disposer le kinésithérapeute pour intensifier les séances ?
MK5
L'importance du traitement médicamenteux qui prenne savoir si tel ou tel médicament favorise ou pas. Par exemple, au niveau de la fréquence cardiaque, ensuite les échelles d'évaluation qui bornent, comme je vous ai dit, de la dyspnée. Après ça, c'est près tout, je n'utilise pas la saturation, pas pour ce type de patient en tout cas. Donc, c'est essentiellement, les échelles. Le ressenti ou l'évaluation de la dyspnée, voilà.

BJM : A votre avis, qu'est-ce qui peut empêcher une augmentation de l'intensité des séances de rééducation ?

MK5
Eh Ben la peur de trop... de faire trop forcer son patient et que derrière il est à soit un malaise cardiaque ou autre par exemple, soit ça lui intensifie les douleurs. Voilà, c'est essentiellement ça. Et aussi on peut rajouter que ça peut être aussi le fait que le patient n'ait pas dégoûté des séances, quoi ! Voilà l'effort ne le rebute pas pour continuer, par exemple, ces séances.

BJM : Est-ce qu'il y a des freins concernant les patients à faire de l'exercice plus intenses ?
MK5
Des freins ? Pour le patient ?

BJM : Pour le patient.
MK5
Ouais, ça va être la douleur et la douleur, ou pendant ou après. Et après, c'est la motivation du patient.

BJM : Est-ce que vous pensez que la sévérité de leur handicap ou bien leur trouble cognitif peut constituer aussi un frein ?
MK5
Pas franchement, non.

BJM : À votre avis, est-ce plus facile pour vous d'intensifier les séances de kinésithérapie à la phase chronique de l'AVC qu'en phase aigüe ? Et pourquoi ?
MK5
Est-ce plus facile pour moi de les intensifier en phase chronique qu'en phase aiguë ? Non, je n'ai pas ce sentiment. Moi je n'ai pas l'impression que ce soit plus facile en phase chronique qu'en phase aiguë. Je n'ai pas l'impression. Parfois même, c'est plus facile en phase aiguë. Pour des patients qui vont être cortiqués et qui sont en fait parfois au tout début, donc bah ils sont forcément en phase aiguë au début de leur maladie, dans l'acceptation ce n'est pas forcément encore ça. Donc, ce sont des gens qui vont être très motivés en espérant récupérer un maximum et le plus vite possible, donc ça c'est en phase aiguë. Pour certains donc à ce moment-là, il n'y a pas de problème de leur faire faire de l'activité physique intense puisqu'ils demandent.

BJM : Selon vous, l'intensification des séances est-elle importante dans la rééducation des patients pour sa rééducation ?
MK5
Si l'intensité de la séance, elle a une importance, c'est ça ?

BJM : Oui, pour les patients dans leur rééducation.
MK5
Ah Ben oui.
MK5
Ah bah oui, oui bien sûr, oui, oui, elle a une importance, oui.

BJM : Et d'après vous, doit-on l'intégrer à la pratique de routine et au traitement kinésithérapique ?
MK5
Ah bah si Ah ouais. Si s'il faut bien sûr. Ouais bien sûr, il faut l'intégrer dans les séances. Ouais, c'est fondamental.

BJM : Donc, pensez-vous qu'elle est de votre ressort de faire des activités intenses à vos patients ?
MK5
Oui, bien sûr.
MK5

Oui, oui, ça fait partie de nos ressorts Oui.

BJM : Et habituellement, qui prescrit ce genre d'exercice, c'est vous ou bien le médecin traitant ?
MK5
Non, c'est nous. Le médecin traitant, il prescrit des séances de rééducation et nous, après on adapte la séance comme on veut quoi !

BJM : En dehors des séances au cabinet prescrivez-vous des exercices intenses que le patient doit faire chez lui ?
MK5
Non parce que les patients que j'ai, j'ai pu le faire avec certains quand ils étaient autonomes et aujourd'hui ils sont plus là et après ils ont pu le faire. Mais pour ceux que j'ai qui sont, qui sont assez lourds ? Non.

BJM : Donc Pensez-vous que vous avez été bien formé en formation initiale, avez-vous besoin de formations complémentaires ?
MK5
Alors, est-ce que j'ai bien été formé en formation initiale ? Bah c'était il y a plus de 20 ans. Donc enfin il y a 20 ans on va dire donc un peu moins même du coup donc. Oh, pas forcément. Non, non. Enfin, en tout cas, c'est plus forcément d'actualité et après je pense que les formations derrière qui sont importantes effectivement ouais pour pouvoir mener... Mais moi enfin voilà, aujourd'hui la pratique que j'ai c'est parce qu'effectivement j'ai fait des formations puis y a mon expertise aussi qui fait que je me suis rendu compte que c'était un facteur hyper important dans la récupération. Mais avec encore une fois avec les données aussi de la science et cetera. Voilà, donc, c'est pour ça que j'ai adapté les séances et je pense que c'est important. Moi de de mon époque, la formation initiale était juste en termes d'activité physique, d'intensité d'activité physique au cours d'une séance en tout cas.

BJM : Donc Pensez-vous qu'un kiné doit avoir une formation en neuro pour pouvoir faire des activités intenses à des patients post AVC en phase chronique?
MK5
Non, je ne pense pas, mais ça je sais pas moi quel est le programme aujourd'hui dans les écoles de kiné ? Mais s'il est évoqué, bah je pense qu'il n'a pas besoin derrière de formation. Mais si on parle des kinés qui sont aujourd'hui sont dans la pratique et sont pas forcément des jeunes kinés on va dire, ouais, je pense que ça peut être pas mal une formation là-dessus, ouais.

BJM : D'accord bon, c'est la fin. De l'entretien donc, je vous remercie d'avoir participé à cet entretien. Donc, souhaitez-vous ajouter une dernière remarque par rapport au thème évoqué aujourd'hui ?
MK5
Bah non, n'y a rien qui me vient, euh non. Voilà, c'est parfait.

BJM : Et avez-vous des questions à me poser ?
MK5
Non, je n'en vois pas Bénédicte du coup

BJM : D'accord, je vais éteindre l'enregistrement.

MK5
Voilà, j'espère que les réponses à ça a été.

Retranscription entretien 6 (enquête 6, MK6)
Entretien réalisé le 06 avril 2023
Durée 28 minutes 33 secondes
Identifications des participants : Bénédicte JEAN MICHEL (BJM), Masseur Kinésithérapeute (MK6)
Fichier audio
audio1817871263.m4a
<u>Transcription</u>
BJM
Pour débuter cet entretien, puis-je s'il vous plaît, vous demandez de vous présenter brièvement, donc votre âge, votre expérience professionnelle, votre formation et si vous avez suivi des formations complémentaires.
MK6
Alors du coup, j'ai 28 ans, je suis kiné depuis 2016. J'ai été étudiant à l'Institut de formation de la Croix-Rouge à Limoges. C'est que des formations par rapport à l'AVC ou complémentaire un peu dans tout je veux dire.
BJM
Par rapport à l'AVC ou autre aussi.
MK6
D'accord, j'ai fait des formations sur les lombalgies, cervicalgie sur l'épaule. J'ai fait une formation, il n'y a pas longtemps sur le pied du sportif. Et après, j'ai fait un congrès sur le membre inférieur aussi, puis voilà. Enfin, j'en ai fait d'autres mais voilà. Pas spécialement sur l'AVC-lib.
BJM
Depuis combien de temps exercez-vous en libéral ?
MK6
Depuis combien de temps en libéral ? depuis 2016. J'ai commencé directement par des remplacements 6 mois et après je me suis installé. Donc début 2017 je me suis installé.
BJM
Sur 100 patients, combien de patients post AVC avez-vous dans votre patientèle ?
MK6
Bonne question. Je dirais entre 5 Et 10% à peu près. 5, ouais, 5, 10. Parce que j'en ai pas mal en maison de retraite.
BJM
Combien de fois par semaine les voyez-vous et combien de temps ?
MK6
Non, en général les AVC, je les vois soit 2 soit 3 fois par semaine. Et je les vois au moins 1h.
BJM
En groupe ou bien en individuel ?
MK6
Souvent en groupe. Souvent en groupe.

BJM
Le voyez-vous sur une durée limitée de séance ou en continu ?
MK6
Ben non, on fait en continu, on va dire toute l'année.
BJM
À la suite des résultats préliminaires de l'étude Observationnelle à laquelle vous avez participé, donc nous avons remarqué que l'intensité des séances différait d'un patient à l'autre. Donc quel regard, portez-vous sur l'intensité des séances de rééducation des patients post AVC ?
MK6
Moi j'essaie de monter un petit peu en intensité en général par rapport à leurs capacités bien sûr, mais de consacrer quand même une partie de la séance à ce qui est plus ou moins enfin assez d'intensité. Ce n'est pas toute la séance mais une bonne partie, ouais.
BJM
Qu'entendez-vous par intensité des séances chez les patients post AVC ?
MK6
Alors, souvent c'est de l'intensité modérée je pense. J'ai déjà eu des patients qui étaient en phase chronique, mais qui étaient plutôt bien, qui étaient assez autonomes, qui pouvaient bouger où là on montait dans l'élevé. Je pense y a quand même un petit peu d'élevé dans la séance pour tous mes patients AVC mais ce n'est pas la majorité du temps, c'est principalement du modéré je pense.
BJM
Quelle importance accordez-vous à l'intensité de la mise en place de vos séances et pourquoi ?
MK6
Je dirais assez importante pour 2 raisons. La première, c'est que j'ai vu l'étude de Stéphanie, donc c'est vrai que maintenant j'apporte un peu plus d'importance à l'époque. J'essayais déjà d'en mettre, mais peut-être moins avant d'avoir vu l'étude de Stéphanie. J'en étais sûrement mais je faisais peut-être plus d'articulaires, mobilisation et cetera. Et c'est vrai que depuis, j'en mets quand même plus. Voilà, mais c'est, je trouve ça assez important.
BJM
D'accord, pourquoi vous vous trouvez que c'est assez important de mettre un peu d'intensité dans les séances ?
MK6
Pour le patient déjà, pour leur autonomie aussi, pour leur regain musculaire, leur regain cardiorespi et cetera. Ça va leur permettre aussi de gagner de l'autonomie pour la maison. Et puis parce que comme tout le monde, ils en ont besoin.
BJM
Connaissez-vous des recommandations en termes d'intensité d'exercice pour cette population ou pas tout ?
MK6
Un petit peu bah ce que j'ai lu dans l'étude de Steph, ce qui m'en reste.
BJM
À votre avis, la séance que nous avons observé avec votre patient était-elle intense ou pas du tout ? légèrement modérée ? Et pourquoi ?
MK6
Normalement, il y avait un peu tout si je dis, pas de bêtises, il y avait du léger, du modéré, de l'intense. Si je me rappelle bien, parce que ça commence à dater, j'avais fait un petit peu d'exercice justement un peu général avec des blaze pods, je ne sais pas si vous connaissez. Mais ce sont des ronds de lumière qui s'allument suivant un programme sur le portable et du coup bah soit contre le

mur, aller les toucher, se baisser, et cetera, soit aussi par terre, sur le mur où je sais que ça le faisait pas mal monter en intensité donc ça j'utilisais pas mal. On avait fait un peu de presse assez lourde, ça ne montait pas. Je ne pense pas que c'était intense, mais au niveau modéré, c'était pas mal. Et on faisait toujours un peu de tapis, mais ça, il le maîtrisait bien, donc je ne suis pas sûr que ça monte plus haut que modéré. Ouais mais encore, c'était quelqu'un qui était déjà très actif à côté, donc c'était plus facile à mettre en place.

BJM

Alors, selon vous, quel est l'environnement technique, matériel et professionnel nécessaire à la mise en œuvre d'intensification des séances chez ces patients ?

MK6

Si j'ai bien tout compris, c'est le matériel et le l'environnement, le matériel qui a besoin pour mettre des séances assez intenses, c'est ça ? Je pense que matériellement je ne sais pas si ça se dit, il n'y a pas forcément besoin d'énormément de choses. Si on veut juste se baser sur l'intensité, il y a moyen de faire monter l'intensité assez vite sans forcément avoir beaucoup de matériel. Ce qui va être important au niveau matériel, je pense, c'est sur la variété des exercices qui va nous permettre de faire plus de choses et que ce soit moins redondant pour le patient, surtout quand quelqu'un est en phase chronique, je pense.

BJM

Par exemple, quel matériel ou auriez-vous besoin pour ce genre de séances.

MK6

Pour ce genre de séance qu'est-ce que j'aurais besoin ? Des blaze pod, j'aime bien. Non, sinon après Bah, un tapis, un vélo un ballon, des haltères, des poids, kettlebell et cetera. Peut-être un gilet lesté pour certains, s'ils ont de la capacité. Après, on est plus limité par notre imagination que par le matériel, je pense sur ce type de rééducation.

MK6

Au cabinet, on a la réalité virtuelle. Je pense que pour ce type de rééducation, c'est pas mal. Ça leur permet un petit peu de sortir de la pathologie et de rentrer plus dans un jeu. Ils vont plus se dépenser sans s'en rendre compte. C'est des plus on va dire.

BJM

Donc, selon vous, quels sont les freins ou obstacles environnementaux à sa pratique et quels sont les facteurs facilitants ?

MK6

Au cabinet ou aussi à la maison ?

BJM

Au cabinet.

MK6

Au cabinet. Il va y avoir la participation du patient, même si c'est nous qui allons devoir aller chercher ça. Le niveau du handicap aussi. Je pense que plus la personne va avoir une hémiplégie importante, plus ça risque d'être compliqué d'aller chercher la haute intensité. Ouais, je pense que c'est principalement ça. La participation du patient et si on peut toujours faire avec le côté sain, monter un peu en intensité mais c'est plus compliqué que si on pouvait utiliser tout le corps entier, quoi !

BJM

Donc quels sont les facteurs facilitants ?

MK6

Facilitant un peu l'inverse. Je dirais-je un patient qui est motivé, qui a envie. C'est vrai qu'on va pouvoir le pousser sur plus de choses plus intenses. Et c'est vrai que comme je dis ce n'est pas un

frein non plus mais c'est plus facile à mettre en place. C'est quelqu'un qui a une autonomie plus importante même si on sait qu'aujourd'hui s'ils viennent nous voir, c'est qu'ils n'en ont pas trop.
BJM
Donc par rapport à l'intensité de vos séances, le manque d'espace disponible et accessible est-il une limite ?
MK6
Oui, je pense, pour la mise en place de certains exercices, surtout si on veut monter un peu en intensité. Il faut tout de suite un petit peu de place. Ouais.
BJM
D'après vous, qu'est-ce qu'il vous faudrait par exemple, en termes d'espace ?
MK6
Pour un seul patient sur des…Alors il faut un peu de place hein ! L'idéal, je pense, ce serait… Je ne sais pas, j'essaie de me pencher un peu sur ce qu'on a nous au cabinet. Mais nous, au cabinet, on a un gymnase qui fait 100 m² et on a plusieurs patients quand on fait des séances de groupe mais pour un patient, combien me faudrait ? En comptant le matériel. Si je comptais le matériel, il me faut au moins 50 m² quoi. Mais si je ne compte pas le matériel, il me faudrait au moins 15, 20 m². Mais après, ça dépend du taux de handicap quoi. C'est ça aussi.
BJM
Donc, par rapport au temps disponible dans une seule séance de traitement, est-ce que le temps constitue-t-elle un obstacle ?
MK6
Certaines fois oui, nous on les garde 1h. Il y en a ça va être largement assez. Il y en a, il y aurait une demi-heure de plus. Je pense que ce serait pas mal, ce serait pas mal.
BJM
Donc, si vous n'avez pas assez de temps, combien vous en faudrait-il ?
MK6
1h30, ça pourrait être pas mal pour certains parce que c'est vrai qu'en 1h on est vite limité. Hein, ça passe très vite. Mais 1h30 pour certains, ce serait bien.
BJM
Comment sélectionnez-vous les patients à qui vous faites faire de l'exercice plus intense ?
MK6
En haute intensité, vous voulez le dire ? Oui ouais, pas forcément modéré, vraiment haute intensité. Comment je sélectionne ? Bah déjà, je vois s'ils arrivent à arriver en intensité modérée, s'ils tiennent bien. Mais dans la mesure du possible, ce serait bien pour tout le monde.
BJM
Oui, c'est quel critère vous vous basez par exemple ?
MK6
Non donc bah à la rigueur qu'ils n'aient pas de problèmes cardio respi. Quels critères ? Bah déjà leur motivation. Mais non, parce que de toute façon je vais essayer d'aller chercher ça chez tout le monde. Et après, c'est vraiment si leur capacité ne leur permet pas de monter une haute intensité, on va commencer par de la modérée.
BJM
D'accord ? Avez-vous besoin que le patient ait effectué une épreuve d'effort ou que le médecin vous ait donné l'autorisation d'intensifier l'effort ?
MK6
Et qui nous est donné l'autorisation ou je dirais non parce qu'en fait, en général, enfin, Oui et non. Parce qu'on va avoir fait notre bilan, de toute façon, initiale, et on va aller chercher ces questions

aussi, savoir s'ils ont des problèmes cardio ou des choses comme ça. Si vraiment ils ont des problèmes cardio, oui, on va quand même demander l'avis du médecin pour au moins savoir les fréquences cibles ou les choses comme ça. Mais s'il n'y a pas de problème sous-jacent je ne vais pas forcément aller embêter le médecin pour lui demander ça.
BJM
Évaluez-vous l'intensité des séances chez vos patients ?
MK6
Non, non, non. J'essaye de chercher l'intensité mais c'est vrai que je ne vais pas évaluer. Pas assez souvent, ouais.
BJM
Dans quels outils d'entraînement utilisez-vous, lors de ces séances ?
MK6
C'est, c'est varié, mais en fait ça, ça dépend vraiment de la personne et ce que j'ai envie de faire. Parce que suivant les personnes en fait, n'y a pas besoin de vraiment grand-chose. On arrive directement dans une séance assez intense. Mais des fois, j'utilise de la charge, des fois j'utilise plutôt des mouvements. Souvent, je charge. Mais et après, je demande de mettre du rythme un petit peu dans l'exercice. Mais ça dépend, ça dépend vraiment des personnes. Ouais, il n'y en a tout simplement rien que la marche, dès qu'on va monter un peu le rythme, ça va suffire. Il y en a, va falloir charger, il y en a, Il va falloir faire des mouvements qui n'ont pas l'habitude de faire. Je me base surtout à l'essoufflement.
BJM
Est-ce que vous utilisez vous du cyclo ergomètre, du tapis roulant ce genre de choses ?
MK6
Ouais bien sûr bien sûr. On a, on a un vélo, on a un cyclo. On a un... comment on appelle ça ? C'est la marque. Mais oui, un cyclo ergomètre, mais là où on peut mettre les fauteuils devant avec les bras, avec les jambes. Un tapis. Ouais, une pu de tout ça, ouais.
BJM
Adaptez-vous l'intensité des exercices en fonction de l'état physique du patient ?
MK6
Tout à fait. Tout à fait. Comme je disais tout à l'heure, ça va dépendre vraiment de la personne. Mais si je peux, j'essaie de quand même monter un peu en intensité, mais ce qui va être intense pour un va être très léger pour un autre. Donc on adapte en fonction de la personne, oui.
BJM
Comment ajustez-vous les séances en matière de durée, d'éternité et de fréquence ?
MK6
Bah ça va être en fonction de mon bilan et de l'évolution du patient sur quoi je me base précisément. Je pense que je regarde un petit peu le ressenti de mon patient, enfin un petit peu. Je regarde le ressenti de mon patient lors des séances, ce que je lui propose. Et toute façon, j'adapte, j'ai toujours adapté les exercices en fonction de la difficulté et je propose quelque chose, si c'est trop dur, un peu plus simple. Si c'est trop facile, je monte un petit peu. Et pour la durée, c'est un peu pareil. Si je vois que la personne, dans tous les cas je les garde 1h, mais si je vois que c'est trop dur pour la personne, bah je fais des pauses plus longues. Et au lieu de faire 6,7 exercices, ils vont faire 3 sur l'heure mais j'adapte en fonction aussi de l'échange qu'on a pendant la séance.
BJM
D'accord leur faites-vous faire un test d'effort sous maximal comme le test de Manche de 6 Min ?
MK6
Non, non, non, non.

BJM
Donc, quels critères de surveillance utilisez-vous lorsque vous êtes intensifiez les séances (fréquence cardiaque, la saturation ou la perception d'effort) ?
MK6
Plutôt la perception d'effort. La perception d'efforts que la personne a et la perception d'efforts que, moi je ressens en voyant la personne, on va dire. Je ne sais pas si je suis clair, c'est souvent ça. Après des fois, je peux utiliser un saturomètre, mais c'est vraiment aussi si je sens la personne vraiment à bout de souffle ou si elle a des problèmes respirants ou cardio. Là je vais utiliser un saturomètre, mais dans la plupart du temps… donc c'est vraiment en fonction de leur essoufflement, leur ressenti, ce qu'ils vont me verbaliser. Et ce que je vois par rapport à eux, parce que ce sont des patients que j'ai quand même régulièrement et des fois depuis un moment. Donc on apprend à les connaître aussi et connaître leur limite.
BJM
Selon vous, quelles sont les connaissances théoriques, techniques et pratiques dont doit disposer le kinésithérapeute pour mettre en place ce genre de séance ?
MK6
Bien connaître son patient, bien effectuer son bilan au début. Poser des questions à son patient sur ce qu'il attend, ce qu'il a envie aussi. L'échange permanent sur tout ça. Après la connaissance de la pathologie, c'est primordial de toute façon et techniquement, comme je vous dis, je pense qu'on peut même sans trop de matériel, on peut déjà faire quelque chose. C'est mieux de diversifier. Comme ce sont des pathologies qu'on a avec des personnes qu'on va voir au long terme, donc c'est bien de pouvoir un peu diversifier ces exercices et proposer des choses différentes. Puis voilà. Technique, connaissance. Ouais, c'est ça.
BJM
À votre avis, qu'est-ce qui empêche une augmentation de l'intensité des séances de rééducation ?
MK6
Des fois, un peu la routine parce qu'on essaie de varier, mais un petit peu. Un confort qui s'installe de Ben je vais faire mes exercices, j'ai progressé mais bah maintenant je suis bien. Et pas vouloir d'un point de vue kiné… ce n'est pas ne pas vouloir mais faire progresser ces exercices des fois. Vous pouvez me rappeler la question, je crois que je m'en éloigne.
BJM
Ce qui empêche une augmentation de l'intensité des séances de rééducation.
MK6
Ça peut être ça. Peut-être un découragement aussi du patient. Des fois, ouais un découragement du patient, un peu trop la routine. Je pense que c'est les 2 facteurs, moi qui m'empêche le plus.
BJM
Pensez-vous que vous avez été bien formé en formation initiale ou avez-vous eu besoin de formations complémentaires ?
MK6
Formation complémentaire.
BJM
Par rapport aux patients, est-ce qu'il y a des freins qui peuvent les empêcher de faire de l'exercice plus intense ?
MK6
Je pense qu'on sur une hémiplégie, on pourra toujours chercher de l'intensité, même si c'est quelqu'un qui est, qui est une grosse et hémiplégie, parce qu'on a toujours le côté simple pour aller chercher quelque chose d'assez intense. Non, je pense qu'on peut toujours aller chercher, ça va

vraiment être la perception de la personne et du kiné après. Mais je pense qu'on on peut toujours aller chercher de l'intensité. Ouais. On trouvera toujours un moyen.

BJM

Est-ce que vous Pensez-vous que la sévérité de leur handicap ou encore leurs troubles cognitifs, leur motivation ou bien leur fatigabilité peuvent constituer un frein ?

MK6

Cognitivement je pense oui. Je pense, parce que si on veut vraiment aller chercher de l'intensité, il faut quand même faire les choses bien et si soit il y a un trouble cognitif ou un trop gros déficit, peut-être qu'on n'arriverait pas à monter assez haut. Mais sinon, après au niveau physique à moins qu'il y a un problème du côté sain, je pense qu'on pourra toujours monter un peu en intensité. Ouais !

BJM

À votre avis, est-ce plus facile pour vous d'intensifier les séances de kinésithérapie à la phase chronique de l'AVC qu'en phase aigüe ? Et pourquoi ?

MK6

Est-ce que c'est plus facile ? Peut-être parce qu'en phase aiguë, on va voir beaucoup d'autres choses à faire, mais ça va être aussi important en phase aigüe, je pense de mettre un petit peu d'intensité. Mais il va y avoir tout, le gain d'amplitude, le gain de commandes, et cetera qui va être important aussi en phase aiguë. Même si c'est toujours important en phase chronique hein ! Mais je pense qu'on peut se concentrer beaucoup plus sur l'intensité après. Même si c'est important avant.

BJM

Selon vous, l'intensification des séances est-elle importante dans la rééducation des patients post AVC ? Et d'après vous, doit-on l'intégrer à la pratique de routine et au traitement kinésithérapique ou bien Pensez-vous que cela devrait être l'objet de séances complémentaires ?

MK6

Oui, c'est important. Oui à la routine, il faut l'intégrer à la routine et faut essayer d'autonomiser le patient pour qu'il fasse chez lui aussi.

BJM

Donc, Pensez-vous qu'il est de votre ressort de faire des activités intenses à vos patients ? Et habituellement qui prescrivent les exercices intenses ?

MK6

Je pense que ce sera plutôt nous. Le médecin, il va faire sa prescription de rééducation post AVC en face chronique et je pense que c'est notre domaine de regarder ce que le patient est capable de faire. Voir avec lui, ce qu'il aime faire aussi et qu'il ne sera pas trop contraignant à faire la maison. Dans le but qu'il fasse le plus souvent possible et c'est nous qui allons faire cette prescription de l'exercice conjointement avec le patient.

BJM

Est-ce que à la maison, vous prescrivez des exercices intenses ?

MK6

Ça dépend des personnes. Je réfléchis. Mais oui, ça m'arrive, ça m'arrive de prescrire l'intense aussi à la maison. C'est rare.

BJM

Leur donnez-vous une dose particulière, en termes de temps, d'intensité ?

MK6

Et je dirais oui, oui. Mais ça va être variable en fonction du patient, OUAIS. Ce qu'il est capable de faire enfin, ça va vraiment être en fonction de ce qu'on arrive à faire au cabinet, hein. Et ce que le patient dit qu'il peut faire à la maison.

BJM
 Avez-vous peur des conséquences qu'il pourrait y avoir chez ces patients, si vous intensifiez les exercices ? Si oui, lesquels ?
MK6
Non, si on fait bien le bilan au début normalement, il n'y a pas de raison.
BJM
Donc, c'est la fin de l'entretien, je vous remercie pour ce temps d'échange. Donc, souhaitez-vous ajouter une dernière remarque par rapport au thème qu'on a évoqué aujourd'hui ?
MK6
Non, non, non, c'est bon.
BJM
Avez-vous des questions par exemple ?
MK6
Non plus, non, non, c'est bon.
BJM
D'accord, je vais éteindre l'appareil.

Retranscription Entretien 7 (Enquête 7, MK7)
Entretien réalisé le 11 avril 2023
Durée 22 minutes 51 secondes
Présentation des interlocuteurs : Bénédicte JEAN MICHEL(BJM), Masseur-Kinésithérapeute (MK7)
audio1953691140.m4a
Transcription

BJM : Pour débuter cet entretien, puis-je s'il vous plaît, vous demandez de façon brève de vous présenter, donc, votre âge, votre date de diplôme, votre expérience professionnelle et si vous avez suivi des formations complémentaires.
MK7
Alors je m'appelle X, j'ai 44 ans, j'ai été diplômée en 2003 donc il y A 20 ans. Et je travaille en en neurologie centrale, je fais de la rééducation en neurologie centrale, en libéral depuis mon diplôme. J'ai fait plein de formations, mais aussi j'ai fait un DIU en communication médicale scientifique en 2007 et un DIU sur les maladies neurodégénératives en 2022. Voilà, et après je donne des cours dans les écoles de kiné. La neurologie, toujours neurologie centrale.

BJM : Depuis combien de temps exercez-vous en libéral ?
MK7
Depuis 20 ans.

BJM : Donc sur 100 patients, combien avez-vous de patients post AVC dans votre patientèle ?
MK7
En post AVC ? Ça doit être 30%.

BJM : Combien de fois par semaine les voyez-vous et combien de temps durent la séance ?
MK7
Alors on les voit entre 2 et 3 fois par semaine et les séances durent 1h30.
BJM
En groupe ou en individuel ?
MK7
En groupe.

BJM : Donc le nombre de séances est limité dans le temps ? Ou voyez-vous ces patients en continu ?
MK7
Ouais oui, on les a en continu.

BJM : Donc, à la suite des résultats préliminaires de l'étude Observationnelle, à laquelle vous avez participé, nous avons remarqué que l'intensité des séances différait d'un patient à l'autre. Quel regard portez-vous sur ce constat ?
MK7
Elle est que donc les résultats, ils diffèrent même au sein de notre cabinet, entre les praticiens, c'est ça. Ou en avec les restes de….

BJM : Par exemple, l'intensité des séances différait d'un patient à l'autre.
MK7
D'un patient à l'autre, Ah oui, d'accord, oui, selon les patients. Enfin, selon les praticiens, oui et oui, selon les patients. Ben, en effet, la problématique du cognitif, elle est, elle est prégnante, hein !
En cette histoire, c'est qu'en gros alors il y a le côté cognitif, il y a le côté motivationnel aussi hein ! Je ne dis pas voilà, mais c'est plus simple en fait d'obtenir des choses au niveau de la motivation, avec des patients qui ont peu de troubles cognitifs, pas ou peu, que des patients qui ont des gros troubles cognitifs. Parce qu'on a beau leur expliquer, essayer de rationaliser, pourquoi ? quelle est l'efficacité ? démontrer de l'entraînement intensif et tout ça, des fois ils n'adhèrent pas, ils comprennent pas. Et enfin, c'est plus compliqué d'obtenir l'adhésion des patients. Quand ils ont des gros troubles cognitifs

BJM : Quel est importance accordez-vous à l'intensité des exercices pour ces patients et pourquoi ?
MK7
Ah Ben pour moi c'est très important parce que c'est ce qui fonctionne donc là pour le coup, je m'appuie un peu sur ce qu'on sait déjà et puis sur aussi un peu de Enfin, je ne sais pas sur mon expérience aussi hein, ça veut dire que les patients qui font des séances intensives, ils progressent plus vite que les patients qui font de la mobilisation quoi, voilà c'est...

BJM : Connaissez-vous des recommandations en termes d'intensité d'exercice pour ces populations ou pas du tout ?
MK7
Au niveau cardio en fait, on s'est beaucoup appuyé sur les recommandations qui ont commencé dans les années 2000, à dire que leur entraînement allait fort intensif, cardio respiratoire et tout ça, ça permettait de faire de la prévention secondaire par rapport aux récidives d'AVC. Et ça, on

s'est beaucoup, nous dans le cabinet… On s'est beaucoup appuyé là-dessus en se disant Bon, Ben on le faisait déjà, mais ça nous a conforté un peu dans notre idée que bah proposer un entraînement cardio, respiratoire, intensif, régulier aux patients cérébrolésés, c'est faire de la prévention secondaire, notamment par rapport aux risques de récidive de l'AVC, quoi !

BJM : À votre avis, la séance que nous avons observée avec votre patient était-elle, intense ou pas du tout ? Légèrement intense, modérément intense.

MK7 : La séance que vous avez observée avec mes patients ?

BJM : Oui, lors de l'étude précédente.
MK7
Dans l'étude précédente. Oui, Ben c'était Ben globalement intense. Ouais quand même.

BJM : Pourquoi, selon vous ?
MK7
Je pense. Si on se base sur les patients lambda, c'est des séances intenses, c'est rare que on a on atteint les séances très intenses. Vous voyez, ce n'est pas non plus très courant.

BJM : Selon vous, quel est le matériel nécessaire à l'atteinte d'un certain niveau d'intensité pendant les séances de rééducation de ses patients ?
MK7
Bon matériel de kiné classique, enfin un chronomètre. Je ne sais pas un minuteur. Voilà pour faire des séries, de fractionner. Des choses comme ça peut être.

BJM : Mais de quel matériel avez-vous besoin pour augmenter l'intensité ?
MK7
Pour augmenter l'intensité ? Moi, il y a un matériel que je n'ai pas et que j'aimerais bien avoir, mais je ne sais pas comment ça s'appelle, c'est des minuteurs qu'on peut programmer pour justement faire des séries de fractionnées. Et tout ça j'en ai. Sur mon téléphone mais. Je sais que ça existe en. Qui fait que ça ? Des sortes d'espèces de chronomètre qu'on peut programmer il y a. Il y a j'ai vu le message le grand truc là en affichage pour la salle de sport pour compter les séries compter enfin voilà. Bah je n'ai pas.

BJM : Donc, vous n'utilisez pas de matériel pour augmenter l'intensité ?
MK7
De matériel, comment vous dites ?

BJM : Est-ce que vous avez d'autres matériels que vous utilisez ?
MK7
Que j'utilise ? Ouais bah les tapis roulants, les cyclo ergomètre membres supérieurs. Enfin, des vélos, du rameur, de l'Elliptique. Les choses comme ça, oui. C'est ça que vous ? Disiez et puis des poids.

BJM : Oui, selon vous, quels sont les freins ou obstacles en termes d'équipement à sa pratique ?
MK7

Qui pourrait manquer chez d'autres kinés pour faire ça ? Rien ! Non, ce n'est pas une question de matérielle hein. S'ils ne le font pas les. Kiné, ce n'est pas pour ça, hein ! C'est une question de temps et de et d'engagement, mais ce n'est pas…

BJM : Donc, par rapport au temps, le temps que vous disposez en séance vous semble-t-il suffisant ?
MK7
Avant, on faisait des séances de 2h jusqu'au COVID. Et quand on a repris après le COVID, on a, on s'est posé, on a réfléchi et 2h c'était long. Les patients passaient beaucoup de temps à se reposer entre les exercices. Enfin voilà, il n'y avait pas, on était moins en fait, on faisait plus longtemps, mais c'était moins intensif et justement, le fait de raccourcir la. Séance. On a intensifié la séance.

BJM : Donc, selon vous, quel est le temps... ?
MK7
1h30, c'est bien.

BJM : Par rapport à l'espace, est-ce que le manque d'espace est une limite ?
MK7
Oui, pour avoir des groupes, oui.

BJM : Donc quel espace ?
MK7
Mais nous, on ne l'a pas, cette limite, nous, on la ressent pas cette limite parce qu'on a beaucoup d'espace, donc ça j'entends bien que d'autres kinés qui ont pas beaucoup d'espace, c'est compliqué de prendre des patients pendant 1h30, plus qu'on peut pas. Voilà, ça je ça OK. L'espace, oui.
BJM
C'est quel espace faudrait-il aux autres kinés pour qu'ils puissent faire ce genre d'exercice ?
MK7
Je pense que l'idéal, c'est 40, 40 m². Par kiné quoi.
audio1781778904.m4a
Transcription

BJM : Y a-t-il un type de patient auquel vous faites faire de l'exercice intense ?
MK7
Non, parce que j'essaie de le faire à tout le monde, mais c'est plus simple quand ce sont des hémiplégiques légers quoi émis par hémi-parétique.

BJM : Donc sur quel critère vous vous basez pour sélectionner ces patients ?
MK7
Non mais en fait non, pardon, en vrai je le fais faire à tout le monde selon le niveau, mais je le fais faire à tout le monde de façon intensive. Non, désolé, je fais vraiment à tout le monde.

BJM : Accordez-vous la même importance à l'intensité de la séance en phase chronique qu'en phase subaigüe de l'AVC ? pourquoi ?
MK7

Non, ce n’est pas la même importance. Parce que pour moi, c'est plus important en phase chronique, parce qu'il y a le côté prévention secondaire, alors qu'en phase aiguë ou subaiguë, on est quand même plus sûr de l'adaptation. Enfin je ne sais pas comment on dit…. On fait une rééducation, peut-être un peu plus qualitative ou voilà. Ouais, plus qualitatif.

BJM : Avez-vous besoin que le patient ait effectué une épreuve d'effort ou que le médecin vous ait donné l'autorisation pour augmenter l'intensité de l'effort ?
MK7
Moi, je préfère. Quand il y a des feux verts de la part des cardiologues et des neurologues. En effet je préfère, mais non, ça ne m’empêche pas, s'il y en a pas, je ne m’empêche pas de le faire quand même.

BJM : Leur faites-vous faire un test d'effort maximal comme le test de la mort de 6 Min ou le test du lever. De chaise ou autre ?
MK7
Oui, on fait ça 6 Min. Le nombre de levées de chaises en 30 secondes ou en une minute ? Je sais plus, mais ça, ça je ne l’ai pas fait souvent celui-là. C'est nouveau, mes collègues, elles font ça. Moi, je fais le 6 Min.

BJM : Évaluez-vous l'intensité des séances chez vos patients ? De quelle manière ?
MK7
Non, je vous pose une question sur le ressenti juste après et après la séance d'après, est-ce qu’ils ont été fatigués de manière excessive ou pas ! Voilà ! Je pose la question 2 fois, une fois, juste après et après à distance.
BJM : Bah quel matériel utilisez-vous pour augmenter l'intensité de vos séances ?
MK7
Des lests enfin les poires et chronomètre.

BJM : Adaptez-vous l'intensité des exercices en fonction de l'État physique du patient ?
MK7
Ah, en fonction de l'état physique. Ah oui, oui, je les adapte. Oui bah oui, oui bien sûr. Oui, je suis obligé, si je veux qu'ils arrivent.

BJM : Comment agissez-vous les séances en termes de durée, d'intensité et de fréquence ?
MK7
Un peu au. Pif, je parce que je ne fais jamais la même chose. Non, je n’ai pas d'échelle de critères pour déceler la fréquence, la durée. Je fais une suite à l'entretien et en négociation avec le patient, ce qui est possible dans sa vie de tous les jours. Quelle place il va y mettre par rapport. On est aussi dépendant des transports, on est dépendant de plein de choses en fait. Donc ce sont plutôt les circonstances qui vont décider la plupart du temps, on n’a pas trop le choix, on est contraint par plein de choses. Ce sont des contraintes variables mais sociétales, sociales, ça quoi ?

BJM : Quels critères de surveillance utilisez-vous lorsque vous augmentez l'intensité des séances ?
MK7
L'essoufflement après exercice et la transpiration. S’il devient tout rouge, je leur dis d'arrêter.

BJM : Bon, c’est par rapport à la perception d’effort ?
MK7
Oui, voilà, c'est ça. Ouais, la perception de l'effort du patient. J'ai un tensiomètre et un saturomètre, mais c'est vraiment si le patient se sent mal que je vais l'utiliser quoi. Mais sinon, je n’ai pas d'instrument de mesure quoi, c'est juste voilà la perception du patient et moi aussi.

BJM : Au cours de votre formation initiale, le niveau d'intensité des séances des patients post AVC a-t-il été abordé ?
MK7
Non, moi, dans ma formation initiale, on disait qu’il ne fallait surtout pas augmenter la spasticité des patients, que c'était très dangereux et qu'il fallait surtout qu’ils ne se fatiguent pas, voilà bon.

BJM : Donc, avez-vous fait des formations complémentaires à ce sujet ?
MK7
Sur l'intensité des séances... Et ce que j'ai fait des formations complémentaires à ce sujet ? Pour le Parkinson, Ouais. Après, j'ai beaucoup lu, j'ai lu des articles, j'ai voilà... Non je n’ai pas fait de formation spécifique sur l'intensité des séances.

BJM : Est-ce que les patients des freins à faire des exercices plus intenses ?
MK7
Ah, ont des freins ! Ah oui. Alors oui, oui, il y en a, oui. Ils ont des freins, ouais. Bah oui, un peu comme chez les valides en fait hein, il y en a qui aiment bien le sport et y en a qui n’aiment pas hein. Donc on a des patients qui jamais ne rentrent dans ce côté intensité. Mais c'est vrai que c'est des patients qu'on a du mal à garder aussi, qui ne viennent pas très longtemps en séance de kiné quoi. Moi je pense y a une question d'appétence aussi avec l'exercice physique, quoi.

BJM : Par exemple, quels sont les freins ?
MK7
Des fois, On a beau savoir. Les freins, Ben. Bon, je pourrais vous parler des côtés cognitifs, mais je ne suis même pas sûre en fait hein ! Bon, il y a le cognitif, c'est vrai que des fois quand on ne comprend pas ce qu'on nous demande voilà... Mais en même temps selon les caractères il y a des patients, ils ne comprennent pas ce qu'on leur demande, mais bon, ils y vont quand même et y en a, ils ne comprennent pas ce qu'on leur demande et arrêtent, ils n’iront pas. C'est une question de caractère. Je pense d’humeur et d'appétence à l'exercice physique. Ceux qui faisaient du sport avant l’AVC, on n'a aucun problème. Ils adhèrent très bien à la rééducation. Ceux qui avaient des métiers sédentaires, c'est plus compliqué. Mais des fois, ils acceptent quand même, hein, mais c'est de la négociation quoi. C'est voilà.

BJM : Et vous, avez-vous des soins à faire faire de l'exercice plus intense ?
MK7
Moi ! Oh, parfois oui. Justement, c'est les patients ne sont pas très motivés et tout ça, Ben ma motivation à moi aussi. Elle peut être un peu bon, c'est rare. Je suis plutôt quand même, plutôt dynamique et plutôt à pousser les gens à se bouger, mais bon, c'est vrai que des fois, devant l'inertie, l'apathie des gens, on a. Du mal à… Ça peut m’arriver.

BJM

Par exemple, est-ce que la sévérité de leur handicap ou leurs troubles cognitifs ou leur fatigabilité, ou la douleur peuvent constituer un frein pour vous ?
MK7
On parle toujours des AVC, hein ? Oui, on n'est pas… Voilà parce que j'ai des patients qui ont des maladies évolutives, mais c'est une autre problématique.
Non, ce n'est pas pour moi.

BJM
Donc il est recommandé de faire 20 Min d'activités physique d'intensité modérée en séance de rééducation pour ses patients. Pensez-vous que l'on doive l'intégrer à la pratique de routine ou au traitement kinésithérapique, ou bien Pensez-vous que cela devait faire l'objet de séances complémentaires ?
MK7
Je ne sais pas. Honnêtement les routines l'auto-rééducation, on s'est un peu formé là-dessus, on nous a beaucoup bassiné là-dessus et franchement, ça marche très peu quoi. J'ai très peu de patients qui acceptent tout ça, ce n'est pas… C'est vraiment. Un très faible pourcentage donc oui, plutôt des séances complémentaires ou de l'APA, quoi. Voilà de l'activité physique adaptée, encadrée par des…Voilà que les patients aient accès, en plus de la rééducation classique de l'APA. Ouais, activité physique adaptée en auto-rééducation, enfin tout ça. Moi, de mon expérience, je n'y crois pas trop quoi, je. Ils ne le font pas ou pas longtemps. Après il se re sédentarise et ils ont les mêmes problèmes quand ils reviennent en fait. Et Ben c'est pour ça que nous on les voit en continu d'ailleurs hein.
BJM
Prescrivez-vous des exercices que le patient doit faire chez lui ?
MK7
Bah non.
BJM
Bon, c'est la fin de l'entretien, donc je vous remercie d'avoir participé. Souhaitez-vous ajouter une dernière remarque concernant le thème évoqué aujourd'hui ou avez-vous des questions à me poser ? ?
MK7
Non, c'est bon. Je vous remercie. Ok très bien et Ben bonne continuation. Bon courage. Merci de vous intéresser à ça. Bon moi je j'adore, c'est bien, ça nous donne du grain à moudre après pour nous les cliniciens donc. C'est super. Merci de ce que vous faites.

Retranscription Entretien 8 (enquête 8, MK8)
Entretien réalisé le 11 avril 2023
Durée 21 minutes 41 secondes
Présentation des participants : Bénédicte JEAN MICHEL (BJM), Masseur Kinésithérapeute 8 (MK8)
BJM

Bon pour débuter cet entretien, puis s'il vous plaît, vous demandez de vous présenter brièvement, donc, votre âge, votre date de diplôme, votre formation professionnelle et si vous avez suivi des formations complémentaires ?
MK8
Je m’appelle X, Kinésithérapeute depuis 20 ans, j’ai eu mon diplôme en 1983, j’ai fait des formations surtout en ergonomie puisque j'ai été consultant et formateur en prévention des risques pour le compte de ma société **** pour le compte de *****pendant une dizaine d’années. Pendant 5 ans pour **** pendant 20 ans pour ma boîte de formation.
BJM
Depuis combien de temps exercez-vous en libéral ?
MK8
Combien ? Bon maintenant je fais que du libéral en ce moment. Donc depuis combien de temps ? Je me suis installé, mon cabinet est installé après Laissac depuis 1985.
BJM
Donc 100 patients, combien de patients post AVC avez-vous dans votre patientèle ?
MK8
Actuellement, ou depuis que je suis installé ?
BJM
Euh depuis que vous êtes installé.
MK8
Aucune idée. J'en ai 20, 15, 20, 30, je n’en sais rien.
BJM
Et actuellement ?
MK8
Comment j'en ai ? j'en ai 3 en ce moment.
BJM
Pour les 3 que vous avez, combien de fois par semaine les voyez-vous ? Et combien de temps dure la séance ?

MK8
La séance dure au cabinet 3/4 d'heure, 1h. Une fois par semaine. 3 fois par semaine, 25 Min.
BJM
Le nombre de séances est-il limité dans le temps ? Les voyez-vous en continu ?
MK8
Patients en continu, depuis des années, oui.
BJM
Hé donc, à la suite des résultats préliminaires de l'étude observationnelle à laquelle vous avez participé, nous avons remarqué que l'intensité des séances différait d’un patient à l'autre. Quel regard portez-vous sur ce constat ?
MK8
Quel regard je porte ? Oui. Je m'adapte en fonction des besoins et du handicap. Après j'ai un Monsieur qui a 50 ans, il marche tout seul donc je lui fais faire des exercices, au cabinet, d'étirement et de renforcement des cuisses, de la cuisse saine. Et le Monsieur qui est plus âgé qui quatre-vingts ans, c'est à domicile. Et maintenant, il veut plus marcher, il a du mal à marcher, il a trop peur de tomber. Et donc je fais la posture en extension du genou droit parce qu'à force d'être assis toute la journée maintenant, il a un flexum du côté sain. Et je le mets debout avec le guidon de transfert. C'est à dire que, il se met debout uniquement avec le guidon de transfert. Et puis je vais lui faire un

étirement du membre supérieur gauche. Ce qui n'est pas le même travail sur les 2. Enfin, le plus jeune, j'ai fait des étirements du membre supérieur, des étirements des muscles du membre inférieur gauche qui est hémiplégique, bien sûr. Mais tout ça, elle l'avait vu, elle l'avait noté.
BJM
Quelle importance accordez-vous à l'intensité des exercices pour ces patients et pourquoi ?
MK8
L'intensité ? Je vais effectuer des exercices de flexion extension. Parce que je considère que les patients doivent continuer de garder la force au niveau du membre inférieur, simplement pour se lever d'abord, pour marcher. L'intensité, c'est du travail à l'espalier. Et après, c'est des étirements, n'y a pas tellement d'intensité, c'est moi qui fait l'effort.
BJM
Connaissez-vous des recommandations en termes d'intensité d'exercice pour ces populations ou pas du tout ?
MK8
Je leur demande de marquer des pauses, ils font des mouvements. Le plus jeune, qui a 50 ans, il fait 10 mouvements à l'espalier, il marque une, il marque une pause. Mais pour tout vous dire, il marque plus souvent de pause qu'il ne fait d'exercice. Donc il ne va pas faire une crise cardiaque devant l'espalier, ça c'est sûr. Mais le problème, le problème, c'est bien la motivation. Très motivé et le plus jeune est beaucoup moins motivé.

BJM
À votre avis ? La séance que nous avons observé avec votre patient était-elle, intense ou pas du tout ?
MK8
Non pas intense. Plutôt moyennement intense.
BJM
Pourquoi elle était moyennement intense ?
MK8
Et quatre-vingts ans, elle était moyennement intense, je le fais, je l'ai fait marcher et elle l'a noté. Et quand elle est venue, il marchait. Maintenant, il veut plus marcher parce qu'il a peur. Donc on ne peut pas parler d'intensité pour la personne, pour le Monsieur qui a quatre-vingts ans.
BJM
Selon vous, quel est le matériel nécessaire à l'atteinte d'un certain niveau d'intensité pendant les séances de rééducation de ses patients ?
MK8
Le matériel nécessaire ? Un tabouret, un fauteuil, un espalier.
BJM
De quel matériel avez-vous besoin pour augmenter l'intensité de vos séances ?
MK8
Je n'ai pas besoin plus de ce que je viens de dire.
BJM
Selon vous, quels sont les freins obstacles en termes d'équipement à sa pratique ? Les freins en termes d'équipement pour la mise en place d'intensité d'exercice.
MK8
Quels sont les freins ?
BJM
Oui

MK8

Je ne vais pas leur mettre du poids sur les épaules pour renforcer la cuisse saine, ça c'est sûr hein ? Parce qu'à côté de ça, Le Monsieur qui 80 ans, il a des troubles, il est diabétique, je ne vais pas lui faire perdre du renforcement autre que le poids du corps.

BJM

En ce qui concerne l'intensité de vos séances, le manque d'espace, est-il une limite ?

MK8

Le manque d'espace si on est sur des sujets jeunes, le manque d'espace est une limite. Certainement je n'ai pas de barre parallèle. Je ne peux pas les faire marcher entre les barres parallèles par exemple. Le cabinet fait 8 M à peu près.

BJM

Dans quel espace vous faudrait-il ?

MK8

Ben pas plus parce que de toute façon le sujet le plus jeune, il marche tout seul. Il marche de lui-même, il va faire ses courses avec son fauteuil roulant, il se lève et il marche chez lui, dehors, dans mon cabinet, il marche, il vient avec sa canne en marchant. Donc je n'ai pas, je n'ai pas lui faire faire des kilomètres hein ! Il est autonome, il vit seul. Je n'ai pas, je n'ai pas allongé la zone de marche du patient.

BJM

Le temps dont vous disposez en séance est-il suffisant ?

MK8

Ben moi, je m'organise en conséquence. Pour le patient qui a 50 ans, il le garde 1h. Le patient, qui est plus âgé, ne vient plus au cabinet, donc avant, je le gardais 1h, maintenant je fais 25 Min à son domicile. Mais je dis bien, je ne le fais plus marcher parce qu'il veut plus, il refuse de marcher maintenant.

BJM

Y a-t-il un type de patient auquel vous faites faire de l'exercice intense ?

MK8

Non ! Jamais d'exercice intense. Toujours des exercices à 220, moins l'âge, moins 20% pour les personnes âgées. Et je les fais reposer au moins une minute pour un retour du rythme cardiaque au rythme de repos.

BJM

Accordez-vous la même importance dans l'intensité de la séance en phase chronique en phase aiguë ? Et pourquoi ?

MK8

Alors en phase chronique et en phase aiguë. Je prévaux à l'intensité et à l'effort, la motivation du patient. Moi, j'ai toujours eu un patient, celui qui a 50 ans, il n'a pas de volonté. Voilà donc il n'y a pas que l'intensité. Ils ne demandent pas à faire de l'intensité, il n'en a rien à faire.

BJM

Avez-vous besoin que le patient ait effectué une épreuve d'efforts, que le médecin vous ait donné l'autorisation pour augmenter l'intensité de l'effort ?

MK8

Non ? Non, ce n'est pas le cas. Dans les 2 cas, il y a un effort qui reste subliminal. Le patient le plus âgé fait des séries de 10. Là je n'ai pas, j'ai pas pris son rythme cardiaque parce que je connaissais pas vos questions. Je pourrais prendre son rythme cardiaque et après je le fais reposer

pendant une minute à chaque fois. Mettre mes patients en danger alors faire faire des exercices assis, debout, assis, debout.

BJM

Leur faites-vous faire un test d'effort sous maximale comme le test de la marche de 6 Min ou le test du lever de chaise ou autre ?

MK8

Je n'ai pas fait faire de test d'effort. Au début, il y a longtemps. Tu fais asseoir, levé, asseoir, levé. Je suis plus tributaire de l'équilibre de la personne.

BJM

Évaluez-vous l'intensité des séances chez vos patients.

MK8

Non, je leur fais faire globalement toujours la même chose.

BJM

Euh, quel matériel utilisé pour augmenter l'intensité de vos séances ? (Ergomètre, tapis roulant ou autres) ?

MK8

Oui, de temps en temps. De temps en temps, s'il y a une sensation de fatigue ou de malaise que je prends la, la tension artérielle. Mais c'est très rare. Car je ne vais pas augmenter l'intensité jusqu'à ce que le patient se sente mal. Je ne prends pas ce risque.

BJM

Adaptez-vous l'intensité des exercices en fonction de l'état physique du patient ?

MK8

Absolument. En fonction de son état physique, son état de santé et en fonction de sa motivation. Parce qu'il n'a pas y a pas que les critères physiologiques qui compte, il y a des critères psychologiques et de motivation qui comptent aussi. Ça, il ne faut pas l'oublier, je ne sais pas si on vous en a parlé mais en kiné on ne nous a pas assez formés au problème de la motivation et du psychisme du patient. Et ça, c'est en ergonomie que Je l'ai appris.

BJM

Oui, par rapport à votre avec par rapport à votre formation, le niveau d'intensité des patients post AVC a-t-il été abordé ?

MK8

Non, vous savez, j'ai été formé en 83 hein, alors ça remonte hein ! Quand je vois, comment elle s'appelle votre collègue qui était venu, Stéphanie, tous les tests dont on parle, c'est vrai que la thérapie a changé. Moi, j'ai beaucoup travaillé sur l'ergonomie et la prévention du risque, l'économie de l'effort, l'adaptation du poste de travail. Et j'ai fait aucune formation complémentaire sur les régimes. Donc, mes connaissances datent de 1983. On ne nous a pas parlé d'intensité.

BJM

Est-ce que les patients ont des freins à faire de l'exercice plus intense ?

MK8

La motivation.

BJM

Est-ce que la sévérité de leur handicap ou la fatigue ou la douleur leur anxiété pourraient constituer un frein ?

MK8

Ah Ben oui, bien sûr. L'état général du patient n'est pas le même. Par exemple le patient le plus jeune. Euh il y a 50 ans, il regarde la télévision jusqu'à 1h du matin. Et puis il y a des jours où il me dit que depuis 4h00 il ne dort pas et par contre l'après-midi il dort 2 ou 3h00. J'ai quelqu'un en

fait qui est complètement, qui a perdu le rythme veille-sommeil. Il est complètement déphasé. Donc c'est sûr que quelqu'un comme ça. Il est addict au tabac. À vos résultats, addict à l'alcool ? Donc c'est quelqu'un qui n'est de toute façon pas en bonne, en très grande santé de toute façon. Et qu'il n'a absolument pas motivé. Alors que le Monsieur qui est à domicile et qui a 80 ans. Ça fait 35 ans que je le vois et tu lui fais faire ses exercices et de la marche. Et donc il peut toujours se lever. Avec le… je l'ai dit, le nom De l'appareil. Le guidon de transfert et parce qu'il a gardé de la force dans les cuisses et dans la cuisse droite et dans le bras droit. Il aide largement sa femme et ça aide beaucoup sa femme pour les transferts. Ce Monsieur a toujours été motivé et Ben il a gardé un petit peu de force suffisamment pour aider sa femme à faire les transferts.
BJM
Les conséquences que cela pourrait engendrer au niveau cardiaque ou autres pourraient-elles être un frein ?
MK8
Il faut respecter, le rythme cardiaque. Il ne faut pas essouffler les gens d'ailleurs. Il faut faire attention à ce qu'il ne soit pas trop essoufflé donc je m'arrête en principe un critère du foie, assis debout, ça suffit, on le laisse reposer une minute derrière. Mais là, je ne connais pas son rythme cardiaque au bout de 10 fois.
BJM
Il est recommandé de faire 20 Min d'activité physique d'intensité modérée en séance de rééducation pour ses patients. Pensez-vous que l'on doit l'intégrer à la partie de routine, au traitement kinésithérapique ou bien Pensez-vous que cela devrait faire l'objet de séances complémentaires, comme des séances d'activités physiques adaptées ?
MK8
Il est recommandé de faire faire des exercices modérés pendant 2 Min au patient, c'est ça ?
BJM
Pendant 20 Min.

MK8
Pendant 20 Min et là on est dans l'éducation, à la santé, là, Oui.

BJM
Pensez-vous que l'on devrait l'intégrer à la pratique de routine ?
MK8
Ne peut pas l'intégrer à la pratique de routine parce que le cabinet est organisé pour faire de la kinésithérapie. Il n'est pas organisé pour faire du coaching d'entretien physique ou modéré ! On s'adresse à un travail utilitaire rappeuse qui va faire un suivi en salle de sport. On ne peut plus le faire dans un cabinet de kiné. Moi je ne pourrais pas le faire. Je ne suis pas organisé pour ça. J'ai trop de pathologie dans une journée pour organiser à faire ça. Cependant, j'ai des personnes qui viennent pour l'aide à la marche, la prévention des chutes, des troubles, de l'équilibre. J'ai des personnes âgées qui viennent. Une petite frange de ma population, ma patientèle, on ne peut pas généraliser, c'est impossible. Vous savez, je suis un cabinet où j'ai 9 postes de travail, j'ai 7 tables et je suis tout seul. Et je m'organise. J'ai toujours plusieurs personnes en même temps.
Effectuer des exercices sous contrôle et sous organisation de ma part. Ce que vous dites, ça allait. 20 Min d'activité modérée. Je le fais faire à certain effectivement. Et ce n'est pas que ça. J'ai des gens qui ont des problèmes d'épaule. J'ai bien ça à l'esprit, on est d'accord et j'ai des personnes qui viennent en gros, c'est ce que je leur fais faire. 45 Min d'activité physique modérée avec des patients post AVC, ouais !

BJM
 Prescrivez-vous des exercices que le patient doit faire chez lui ?
MK8
 Est-ce que je prescris des exercices que le patient doit faire chez lui ? Ah Ben bien sûr, Ben c'est évident. Tous les patients qui viennent chez moi repartent avec des exercices. Ils sont capables de faire en ma présence et qu'ils sont capables de faire chez eux, et c'est vivement recommandé. Ça s'appelle l'éducation à la santé et aux exercices d'entraînement et adaptés à leur pathologie ou à leurs problèmes. Tous mes patients.
BJM
 À quelle fréquence, quelle durée et à quelle intensité ?
MK8
 Je leur dis de faire ça 2 fois par jour. Je ne parle pas d'intensité. Je travaille chez eux au même rythme, qui travaillent chez moi et je n'ai pas un carnet, je leur ne demande pas de noter le temps qu'ils ont passé à effectuer des exercices. Par contre, je m'assure qu'ils ont bien fait les exercices. Franchement, certains disent je n'ai rien fait. Et puis ceux qui disent oui, oui je le fais régulièrement et ceux qui disent le faire régulièrement, on se rend compte qu'ils savent. Ils savent très bien, ils ont très bien compris l'exercice qu'on demande. Je pense notamment. Abaissement de l'épaule pour décoincer les tendons de la coiffe des rotateurs. Au niveau de l'épaule, c'est quelque chose que je fais faire régulièrement et que les gens font chez eux également. Le pendulaire aussi. Les 3/4 des gens qui viennent nous voir au cabinet sont motivés pour pas dire les 9/10.
BJM
Bon, c'est la fin de l'entretien, je vous remercie d'avoir participé, donc souhaitez-vous ajouter une dernière remarque concernant le thème que nous avons évoqué ou avez-vous des questions ?
MK8
 Le 220. Bon cela ils ne vont jamais se faire de violence. Il n'a pas beaucoup après, dans les motivé. et dans les motivé faut être prudent parce que quand il fait monter quelqu'un sur le vélo par exemple. Hé. Au cyclo, vélo justement, qui permet de calmer un rythme.
BJM
Je vous remercie, je vais éteindre l'appareil d'enregistrement.

Retranscription Entretien 9 (Enquête 9, MK9)
Entretien réalisé le 13 avril 2023
Durée 17 minutes 10 secondes
Présentation des interlocuteurs : Bénédicte JEAN MICHEL (BJM), Masseur-Kinésithérapeute (MK9)

Fichier audio
audio1379270002.m4a
Transcription

BJM : Pour débuter cet entretien, puis s'il vous plaît, vous demandez de vous présenter de façon à ces brèves votre âge, la date de votre diplôme, votre expérience professionnelle et vos formations complémentaires.
MK9
D'accord, mais je suis X, j'ai 41 ans, j'ai mon diplôme en 2005 à Limoges. Après j'ai fait une formation d'ostéopathe o j'ai fait des formations de thérapie manuelle vestibulaires. Me voilà à peu près.

BJM : Depuis combien de temps exercez-vous en libéral ?
MK9
Mais depuis 2005 donc ça fait 18 ans maintenant.

BJM : Combien de patients post AVC suivez-vous actuellement.
MK9
Je vais en avoir 6, 7, pardon.

BJM : En proportion, combien cela représente dans votre patientèle ?
MK9
Ouf, une question, on ouais, je ne sais pas 4 à 5%.

BJM : Combien de fois les voyez-vous par semaine et combien de temps durent la séance ?
MK9
Une demi-heure et généralement c'est 2 ou 3 fois par semaine.

BJM : Est-ce un groupe ou en individuel ?
MK9
En individuel

BJM : Sur combien de temps les voyez-vous habituellement ?
MK9
Je fais 2, 3 séances par semaine chacun.

BJM : Est-ce des séances limitées dans le temps ou voyez-vous ses patients en continu ?
MK9
En continue.
BJM : Donc les voyez-vous sur plusieurs mois ou sur plusieurs années ?
MK9
Plusieurs années généralement.

BJM : Connaissez-vous des recommandations en termes d'intensité d'exercice pour cette population ou pas du tout ?
MK9
Ben pas forcément non. C'est leur visage qui vous me dire s'ils sont fatigués.

BJM : Accordez-vous à l'intensité des exercices pour ces patients et pourquoi ?
MK9
Après, je regarde aussi, ils sont fatigués ou pas parce qu'après, s'ils sont trop fatigués, on ne peut plus rien faire donc ce n'est pas l'utilité de venir si c'est pour rester assis à rien faire donc c'est important de voir leur niveau de fatigue et s'adapter en fonction.

BJM : À la suite des résultats préliminaires de l'étude Observationnelle à laquelle vous avez participé, nous avons remarqué l'intensité de ces séances différait d'un patient à l'autre. Quel regard, portez-vous sur ce constat ?

MK9 : Ben c'est qu'ils n'ont pas forcément tout le même âge, ils ont pas tous la même activité, ils n'ont pas forcément les mêmes séquelles de l'AVC donc ça s'explique assez facilement comme ça. Quoi ! Des personnes les plus touchées, qui sont plus grabataires mais s'active plus vite que les patients qui ont un AVC avec moins de séquelles, les plus jeunes, je pense.

BJM : Nous avons récemment envoyé le bilan de la séance que nous avons observée avec votre patient. À votre avis, la séance que nous avons observée est-elle intense ? Ou pas du tout ?
MK9

BJM : Qu'est-ce qu'une séance intense selon vous ?
MK9
Ah Ben une séance intense. Et quand le patient ressort fatiguer et trop fatigué, quoi peut être pour, après avoir du mal à bah à faire des activités justes après quoi !

BJM : Selon vous, quels sont les freins ou obstacles en termes d'équipement à la mise en œuvre d'une séance avec un certain niveau d'intensité.

MK9 : La condition physique du patient, ce sont les activités quotidiennes. Aussi savoir bah si les avantages ou pas, enfin plus sa motivation aussi c'est. Important parce que. S'il n'est pas forcément motivé, c'est plus gros frein ça.

BJM : Et avez-vous besoin de matériel spécifique pour atteindre un certain niveau d'intensité ?
MK9
Non, pas spécialement.

BJM : Selon vous, quel est le matériel nécessaire à l'atteinte de certains niveaux d'intensité pendant les séances de rééducation de ces patients ?
MK9
Ben non. Et puis le patient après.

BJM : Après vous n'avez pas besoin de matériel pour faire ce genre d'exercice ?
MK9
Si j'en ai. Mais après c'est plus accessoire quoi. Vous voyez, j'ai un Uber 360. J'ai la réalité virtuelle aussi. Ce qui est pas mal pour faire des exercices et tout ça, c'est ludique et. Ça en tient, un peu plus, on va dire concentré sur l'exercice et moins sur leur niveau de fatigue, mais après sans ça, on peut te faire travailler aussi avant. Je n'avais pas ça et ça allait bien quoi, c'est un plus en fait. En faisant les exercices simples et en restant avec le patient. Je pense qu'on peut y arriver aussi quoi ! C'est sûr que c'est mieux d'avoir ça, mais ce n'est pas forcément indispensable.

BJM : En ce qui, concerne l'intensité de vos séances, le manque d'espace est-il une limite ?
MK7
Non

BJM : Le temps dont vous disposez en séance vous semble-il suffisant ?
MK9
Oui, parce que je reste une demi-heure, donc je pense que ça va après, les gens se démotivent assez rapidement, surtout quand ça l'est fatigue un peu.

BJM : Y a-t-il un type de patient auquel vous faites faire de l'exercice intense ?
MK9
Oui, mais quand les patients ont peu de séquelles en fait post AVC et qu'ils ont plus des troubles un peu d'équilibre ou un peu de perte. Et puis, en fonction de l'âge aussi, là j'avais un patient, je ne sais pas si qu'il y avait à 50 ans, donc lui je lisais faire pas mal de renforcement. Et de choses comme ça, quoi ! Déjà chez lui, il travaillait beaucoup, donc. C'est, c'était en fonction de ces activités aussi à la maison quoi.

BJM : Outre le critère de l'âge avez-vous, d'autres critères sur lequel sur lesquels vous vous basez pour sélectionner ses patients ?
MK9
Oui, je vous dis, l'activité quotidienne. Enfin leur activité quotidienne.

BJM : Accordez-vous la même importance à l'intensité de la séance en phase chronique qu'en phase subaigüe après AVC ?
MK9
Ben non parce qu'enfin en en phase chronique, c'est plus de l'entretien, donc il faut voir ça sur du long terme. Et un post AVC, juste après les séances, il y a une récupération à voir donc ce n'est pas forcément le même type de séance. Bien, et ce n'est pas le même mode d'exercice, il y a moins d'entretien à faire, c'est plus de la récupération avant.

BJM : Avez-vous besoin que le patient ait effectué une épreuve d'effort, que le médecin vous ait donné l'autorisation pour augmenter l'intensité de l'effort ?
MK9
Bah ça serait pas mal, mais après, on n'en a jamais donc on fait sans.

BJM : Donc vous ne faites pas de test d'effort sur maximal comme le test de la marche de 6 Min ou le test de levée de chaise ?
MK9
Non, mais j'ai un saturomètre avec une prise de pouls. Donc je vois avec la fréquence cardiaque et je vois s'il se fatigue un peu trop ou plus en fonction des signes cliniques aussi. Quand il respire, plus j'arrête.

BJM : Évaluez-vous l'intensité des séances chez vos patients ?
MK7
Oui

BJM : De quelle manière ?
MK9
Mais du coup, je vous dis avec la fréquence cardiaque ou après, après bah avec les signes cliniques, l'essoufflement et la rougeur et la fatigue quoi ! La motivation aussi, parce que quand ils sont fatigués et ils ne sont pas motivés pour continuer, ils s'arrêtent souvent.

BJM : Utilisez-vous des matériels pour augmenter l'intensité de vos séances ?
MK9
Bah du coup oui, le saturomètre quoi !

BJM : Est ce que vous utilisez le Cyclo Ergomètre ou le tapis roulant ?
MK9
Ah oui, oui, pardon, oui, oui. Oui, oui, oui.

BJM : Donc, lorsque vous prévoyez un exercice intense ou un patient, sur quel critère adapté vous l'intensité ?
MK9
Ben ça, la fréquence cardiaque.
BJM : Adaptez-vous l'intensité des exercices en fonction de l'état physique du patient ?
MK9
Oui

BJM : Et comment les ajustez-vous ?
MK9
Ben en fait, en fonction de son état physique, parce que des fois ils sont assez malins. Ils simulent un peu pour pas travailler, donc c'est pour ça que la fréquence cardiaque est assez importante parce qu'on peut vérifier si Ben il joue un petit peu ou pas quoi ! Donc voilà. Pour pas travaille.

BJM : Quelques critères de surveillance utilisés lorsque vous augmentez l'intensité des séances ?
MK9
Ouais, la fréquence cardiaque, ouais. Et mais après l'état physique aussi bon.
BJM : Vous sentez-vous compétent en matière d'augmentation de l'intensité en séance de ces patients ?
MK9
On oui, je pense. Je n'ai tué personne encore, donc ça va.

BJM : Au cours de votre formation initiale, le niveau d'intensité des séances des patients post AVC a-t-il été abordé ?
MK9
Non, pas spécialement. Après c'était en général pour tous les patients. Ouais, on n'a pas eu trop de…Non, je ne pense pas.

BJM : Donc, avez-vous fait des formations complémentaires à ce sujet ? Ou bien êtes-vous allés à des congrès sur l'intensité des séances ?
MK9
Bon, j'ai participé à une étude récemment sur ce sujet.

BJM : Est-ce que les patients ont des points à freins es exercices plus intenses ?
MK9
Oui. Bah après, c'est surtout en fonction de leurs besoins mais là j'ai un patient en tête qui est qui, bah bougeait plus de son de chez lui parce qu'il avait du mal au niveau des transferts parce qu'il avait du mal à se tenir sur ses jambes et tout donc de faire travailler ces gens. Mais il a compris que ça aller à faire des transferts, donc un peu plus d'autonomie donc. Maintenant, il est content de faire ses exercices.

BJM : Les patients vous vont-ils ressentir des peurs, des approches, appréhensions, un manque de motivation à l'idée de réalité, des exercices d'un certain niveau d'intensité ?
MK9
Ouais bah ils ont peur de l'échec, toujours parce que ça leur rapporte, ça les ramène à leur pathologie, en fait donc. Et donc faut essayer de les motiver dans le bon sens, en les encourageant et en évitant de les mettre dans une situation d'échec. Donc pas en mettant des exercices trop intenses au début pour éviter que Ben tout de suite soit dans l'échec et du coup, Ben dans la démotivation après.

BJM : Et vous, avez-vous des freins à faire de l'exercice plus intense à ses patients ?
MK9
Non, non. Je veux bien qu'il transpire et que je me repose.

BJM : La sévérité du handicap pourrait-elle être un frein ?
MK9
Euh non, après faut adapter en fonction des patients, quoi, c'est tout.

BJM : Et par rapport au trouble cognitif ?
MK9
Ou ça, ça freine. Oui, un peu oui, mais après, on arrive toujours à bah à s'adapter. Enfin, c'est après, c'est à nous de nous adapter à ça quoi ! Mais ça n'empêche pas de faire des renforcements après faire des séances plus intenses.

BJM : Est-ce que la motivation, la fatigue, la douleur ou l'anxiété pourrait constituer un frein pour ses patients ?
MK7
Oui
BJM : Les conséquences que cela voudrait engendrer au niveau cardiaque autres pourraient-elles. Être un frein ?

MK9 : Ouais oui, à force. Fatalement, si vous allez trop loin, ça peut effrayer s'il meurt. Mais faut éviter d'aller justement dans ces tranches, quoi ! Il faut que ça reste intense, mais pas une avec une fréquence cardiaque maxi quoi ! Comme généralement, ce sont des patients qui sont assez grabataires et qui n'ont pas plus trop l'habitude d'activité physique. Il faut y aller progressivement.

BJM : Il est recommandé de faire 20 Min d'activité physique d'intensité modéré en séance de rééducation pour Sébastien. Pensez-vous que l'on doive l'intégrer à la pratique de routine, au traitement kinésithérapique, ou bien Pensez-vous que cela devrait faire l'objet de séances complémentaires, comme des séances d'activités physiques adaptées ?
MK9
Moi je pense que c'est bien de la mettre avec les séances de kiné, ça fait partie en mélangeant justement avec de la proprio quoi. Ça égaie un peu les séances quoi.

BJM : Prescrivez-vous des exercices que le patient doit faire chez lui ?
MK7
Oui

BJM : À quelle fréquence ?
MK9
En Ben généralement je leur dis de faire ça tous les jours pendant 5 Min à peu près quand Ben après ça dépend aussi des patients. Généralement, c'est ça.

BJM : Et à quelle intensité ?
MK9
Ben reproduire les séances qu'on fait un peu au cabinet mais dans des conditions un peu plus adaptées. Donc, quand il y a un trouble de l'équilibre bien assis, donc c'est un peu plus faible aussi quoi ! Modéré on va dire.

BJM : Donc, c'est la fin de l'entretien, je vous remercie pour ce temps d'échange passé avec donc souhaitez-vous ajouter une dernière remarque concernant le thème que nous avons évoqué aujourd'hui ?
MK9
Ben si vous avez besoin. D'autre chose, vous me dites. Il n'y a pas de souci.

BJM : D'accord ? Où avez-vous des questions à me poser ?
MK9
Non, c'est bon.

Retranscription Entretien 10 (Enquête 10, MK10)
Entretien réalisé le 13 avril 2023
Durée 34minutes 34 secondes
Présentation des interlocuteurs : Bénédicte JEAN MICHEL (BJM), Masseur-Kinésithérapeute (MK10)
Fichier audio
audio1083033493.m4a

BJM : Pour débuter cet entretien, puis s'il vous plaît, vous demandez de vous présenter de façon assez brève, votre âge, la date de votre diplôme, votre expérience professionnelle et vos formations complémentaires.
MK10
Alors j'ai 46 ans bientôt, j'ai donc j'ai fait mes études en Belgique. Je suis diplômée de 1999. Et j'ai travaillé presque 20 ans dans un centre de rééducation fonctionnelle. Donc j'ai l'occasion bien sûr de travailler beaucoup neuro. Et aujourd'hui, je suis dans un cabinet en libéral et je travaille depuis bientôt 6 ans. Voilà donc j'ai une patientèle assez variée, il me reste encore un petit peu de neuro. J'ai quelques patients chroniques sur l'hémiplégie donc des AVC notamment bien sûr. Et voilà donc en en formation complémentaire, c'est plutôt du Mackenzie ou CGE pour l'épaule, mais autrement le gros de mes formations en euros, c'était durant. Euh, mon exercice en éducation fonctionnelle, en Charente, voilà.

BJM : Combien de patients post AVC suivez-vous actuellement ?
MK10
Alors le patient ? Le nombre de patients, alors j'en ai un, je n'en ai pas beaucoup, j'en avoir 2 ou 3 pour l'instant actuellement, hein !

BJM : Combien cela représentera votre patientèle ? En termes de pourcentage.
MK10
Oh là là, Ben je ne sais pas, ça doit faire pour l'instant 2%. 2 ou 3%, ça ne fait pas beaucoup, c'est variable hein. Et là je suis sur des patients chroniques. Le Monsieur qui a été vu par Stéphanie par exemple, je le vois plus. Donc voilà, c'est vrai que ça reste un pourcentage très restreint par rapport à mon exercice que j'avais dans le centre de rééducation, ouais.

BJM : Combien de fois les voyez-vous par semaine et combien de temps dure la séance ?
MK10
Je les vois 2 fois par semaine, 1h. 2h par semaine.
BJM : Est-ce un groupe ou en individuel ?
MK10
En individuel.
BJM : Sur combien de séances les voyez-vous habituellement ?
MK10
Bah 2 par semaine.
BJM : Est-ce un nombre de séance limité dans le temps, où voyez-vous ses patients en continu ?
MK10
Ah non, c'est chronique, c'est toutes les semaines ils sont récurrents.
BJM : Donc, vous les voyez sur plusieurs fois par mois ou sur plusieurs années ?
MK10
Ah oui, sur plusieurs années.
BJM : Donc, connaissez-vous des recommandations en termes d'intensité d'exercice pour cette population ou pas du tout ?
MK10
Les recommandations d'exercice Bah... Ah donc là, ce que j'ai expliqué, c'est que je devais redéfinir à chaque séance ou à peu près des objectifs réalisables pour le mot patient, pour qu'ils ne se trouvent pas en situation d'échec, parce que bien souvent, ils ont une baisse de morale par rapport à leurs incapacités dans leur quotidien. Et donc les exercices doivent être adaptés pour leur permettre de d'avoir un sentiment de progresser. Donc, que ce soit sur le plan physique ou même moral ?

BJM : Quelle importance accordez-vous à l'intensité des exercices pour ses patients et pourquoi ?
MK10
Eh bien, j'ai répondu un petit peu à la question avant, ce sont des exercices qui ne doivent pas durer trop longtemps non plus parce que sur le plan fatigue musculaire, ils peuvent vite être arrivé à leurs limites. Et puis ils ne doivent pas trop s'ennuyer non plus, donc je leur, je leur laisse à peu près un nombre de répétitions sur les exercices de renforcement musculaire ou après d'équilibre, j'essaie de varier à peu près toutes les toutes les 5 Min, Quoi, 5 10 Min.

BJM : À la suite des résultats préliminaires de l'étude Observationnelle à laquelle vous avez participé, nous avons remarqué que l'intensité de ces séances différait d'un patient à l'autre. Quel regard portez-vous sur ce constat ?
MK10
Eh bien, encore une fois, ça dépend des objectifs du patient, des capacités de mon patient. Je sais que certains n'auront pas les mêmes capacités motrices physiques que d'autres. Donc je ne vais pas pouvoir adapter ma séance de la même façon.

BJM : Nous avons récemment vu le bilan de la séance que nous avons observé avec votre patient Mr X. À votre avis, la séance que nous avons observée était-elle, intense ou pas du tout ?
MK10
Ben disons que la séance que j'ai faite avec lui n'était pas très intense. En plus, c'était un petit peu adapté. Après, avec le test donc. Même si on essaie de rester sur des exercices qui se rapprochent de de ce qu'on fait actuel. Enfin, CE Monsieur, je le vois plus, hein, de toute façon, mais non, ce n'était pas complètement différent de ce qu'on faisait habituellement. Donc oui elle s'en rapprochait.

BJM : Donc pourquoi, avec ce Monsieur, la séance n'était-elle pas intense ?
MK10
Eh bien. Parce que derrière, je pense que c'était pour lui permettre d'être encore alerte par rapport à au bilan qui allait y avoir avec Stéphanie. Pourquoi est-ce que ce n'était pas intense et pourquoi est-ce que j'ai l'impression que la séance peut l'être davantage ? Parce que oui, c'est ça. Il faut tout mettre en place. Donc, en fonction de la fatigue du patient, par exemple s'il a une infection, s'il est fatigable déjà avant d'arriver, s'il a une douleur quelconque, ça peut entraver la séance et donc j'essaie de m'adapter. Et si je vais essayer de lui faire travailler plus sur le plan physique, donc cardiovasculaire, les déplacements des transferts et tout ça, ça va lui demander une source d'énergie plus importante. Et puis voilà, il faut aussi que j'arrive à le faire travailler sur le plan moteur, en analytique. Je le fais sur table. Après, si je le mettre en place un peu plus d'équilibre, tout ça, ça lui demande beaucoup d'efforts. Ça peut être une séance plus complète, ouais.

BJM : Qu'est-ce qu'une séance intense selon vous ?
MK10
Ben une séance intense, c'est une séance qui doit avoir un rapport cardiovasculaire plus intense quoi ! ça veut dire que déjà, sortir de son fauteuil marche et se déplacer. Tous ces transferts, tout ça ce sont déjà des choses qui sont fatigantes.

BJM : Selon vous, quels sont les freins ou obstacles en termes d'équipement à la mise en œuvre d'une séance avec un certain niveau d'intensité ?
MK10
Ben il faut déjà avoir si l'endroit est adapté. Donc s'il y a un seuil à franchir en rentrant dans le cabinet s'il y a par exemple là dans notre cabinet, il y a un plan incliné qu'il doit utiliser pour accéder à la rampe de marche. Euh donc tout ça peut être des obstacles et des freins, mais ça peut être aussi des éléments de travail quoi, des objectifs à atteindre pour notre patient.

BJM : Et voir en pratique, avez-vous besoin de matériel spécifique pour atteindre un certain niveau d'intensité ?
MK10

En soit du matériel, on n’en a pas besoin énormément. On s'adapte avec l'environnement ou on peut faire une séance debout. Voilà d'équilibre. On peut faire du travail assis, debout, avec ou sans accoudoir, en utilisant que le fauteuil du patient. S'il arrive déjà avec un fauteuil ou s'il arrive sans son fauteuil, voilà après, si on a une table qui est électrique ou pas, ça va changer aussi les choses par rapport au transfert. Donc oui, après au plus on a de petits matériels et de coussins adaptés pour installer notre patient correctement, ça va faciliter les choses, mais je reste persuadée qu'on peut s'adapter en toute circonstance, que ce soit dans un cabinet avec beaucoup de matériel ou avec peu de matériel ou juste à 2.

BJM : En ce qui concerne l'intensité de vos séances le manque d'espace est-il une limite ?
MK10
Ben en fonction des objectifs. C'est effectivement Variable. Mais non, si je n’ai pas beaucoup d'espace, je peux m'adapter encore une fois. On va travailler d'autres choses quoi.

BJM : Le temps dont vous disposez en séance vous semble-t-il suffisant ?
MK10
Eh bien, pas toujours, parce que le temps que j'ai à disposition pour mon patient est variable en fonction aussi des aléas, c'est-à-dire ? Que le patient n'est pas toujours à l'heure ou moi même que. On va avoir peut-être plus de difficultés ce jour-là sur un transfert parce qu’il est fatigable ou parce qu’il y a eu un accident. Voilà une fuite ou autre, et donc il faut qu'il passe plus de temps aux toilettes. Enfin voilà ce sont des aléas qui peuvent arriver quoi hein ! Donc mais le temps que je dispose globalement est suffisant normalement pour faire ce que j'ai à faire et pour faire travailler mon patient, oui.
Alors reste globalement une demi-heure, vraiment avec mon patient et sur l'heure totale, je le fais travailler tout seul, peut-être 20 Min quoi. Et les 10 Min qui restent vont correspondre à son installation entre la sortie du taxi VSL et de l'endroit où il est dans le cabinet pour rejoindre son VSL quoi.

BJM : Y a-t-il un type de patients auquel vous vous faites faire de l'exercice intense ?
MK10
Oui, ça va être sur des patients qui ont beaucoup plus de capacités motrices. Peut-être plus jeune. Moi, euh. Monsieur X c'était quelqu'un qui était quand même rapidement fatigable, qui avait peu de capacités motrices. Qui voulait néanmoins rester le plus autonome possible. Donc voilà, j'ai une autre dame qui est assez fatigable mais parce qu'elle est relativement âgée.

BJM : En plus de du critère de l'âge, avez-vous d'autres critères pour sélectionner ces patients ?
MK10
Pour avoir une séance plus intense. Eh bien, euh. Il faut, je pense. Éviter que voilà y ait des contraintes, comme la spasticité qui peut entrer en ligne de compte. Après voilà, on peut faire une séance plus intense sur table. Si on fait faire les transferts ou travailler le relevé du sol, tout ça, ça peut être très intense mais du coup peut durer moins longtemps sur la séance. C'est une dépense énergétique qui va être variable en fonction du type de patient. Si c'est un patient qui doit absolument être capable de rentrer par exemple dans une voiture avec des contraintes, ça va être un objectif de travail. Donc il faut qu'on soit adapté aux besoins du patient.

BJM : Accordez-vous la même importance à l'intensité de la séance en phase chronique, en phase subaiguë après AVC ?

MK10
Ah oui, oui, il ne faut peut-être pas trop fatiguer le patient en post AVC. Même si la simulation reste très importante sur les 6 premiers mois, le patient doit récupérer un maximum. Si c'est possible, mais encore une fois, on va essayer de pas trop le fatiguer sur le plan cardiovasculaire, je pense aussi et sur le plan musculaire, il ne faut pas que ça soit trop fatigable pour éviter qu'il y ait une augmentation la spasticité. Si toutefois il en y a. Après en post AVC plus tardif après 6 mois, je pense qu'on peut pousser un peu plus l'intensité du travail. Ouais je pense à un autre patient qui est plus jeune actuellement et qui est capable sur 1h de faire des passages d'obstacles, de monter descendre une marche, de faire un petit peu de travail, de coordination, d'échelle, de rythme, mais parce qu'il a déjà beaucoup plus de capacités que et il est plus jeune, donc voilà, avec ce patient-là, je vais faire un travail plus intense physiquement, oui.

BJM : Avez-vous besoin que le patient ait effectué une épreuve d'effort ou que le médecin vous ait donné l'autorisation pour augmenter l'intensité de l'effort ?
MK10
Et bien je vais quand même me renseigner sur l'origine de son AVC, mais c'est toujours plus rassurant d'avoir ce type d'élément. Après, si je ne les ai pas en ma possession, je vais quand même me méfier et surveiller mon patient. Je ne vais pas lui faire faire un relevé du sol ou un retournement tout de suite. Je vais d'abord faire connaissance sur quelques séances avant de pousser l'effort. Ça c'est sûr.

BJM : Leur faîtes-vous faire un test d'effort maximal comme le test de marche de 6 Min ou le test du levée de chaise ou autre ?
MK10 :Non, je n’ai pas de test spécifique mais par expérience je pense savoir si le patient va au-delà de ses limites ou pas. Donc j'y vais doucement, quoi. Je fais connaissance.

BJM : Évaluez-vous l'intensité des séances chez vos patients ?
MK10
Non, je n’ai pas de test spécifique.

BJM : Donc, quel matériel utilisez-vous pour augmenter l'intensité de vos séances ? Est-ce que vous utilisez des cyclo ergomètres, tapis roulant ou autre ?
MK10
Alors, le tapis roulant, j'évite parce que j'ai déjà eu une mauvaise expérience. Euh donc je n'utilise plus le tapis roulant, non. Le tapis de marche, non. Le vélo, pour ceux qui peuvent un petit peu oui. Mais pas de tapis de marche, non.

BJM : Lorsque vous prévoyez un exercice intense pour un patient, sur quels critères adaptez-vous l'intensité ?
MK10
Eh Ben sa fatigabilité, l'état de ses fatigues motrices. Sa fatigabilité en général, de sa fatigabilité motrice. Ouais, c'est plutôt ça.

BJM : Adaptez-vous l'intensité des exercices en fonction de l'état physique du patient ? Comment ajustez-vous les séances en termes de durée, d'intensité et de fréquence ?
MK10

Eh bien, j'y vais progressivement. Je travaille d'abord sur table, allongée puis assise et puis debout. Je peux rajouter des contraintes avec la pesanteur en fonction des postures. Et je vais éviter de mettre trop de résistance. Avec des poids hormis le poids du corps quoi. Là je ne veux pas laisser le patient.

BJM : Quels critères de surveillance utilisez-vous lorsque vous augmentez l'intensité des séances ?
MK10
Eh bien, encore une fois, je vais rester à côté de mon patient. Je le connais et donc je vois s'il est en capacité sur le nombre de répétitions ou sur des compensations qu'il va avoir et donc je vais me dire, là, il est temps qu'il arrête ou il va faire une petite pause, il recommencera après.

BJM : Donc vous utilisez la perception d'effort du patient ?
MK10
Oui
BJM : Vous sentez-vous compétent en matière d'augmentation de l'intensité en séance de ses patients ?
MK10
Bah, encore une fois je ne sais pas, je vais voir jusqu'où il peut aller. Mais bien souvent ce sont des patients chroniques, je ne vais jamais vraiment aller au-delà de ce qu'on connaît et de ce qu'on a déjà fait ensemble, hein. Il m'est arrivé d'emmener mes patients à l'extérieur du cabinet, pour faire par exemple une marche dans le quartier. Et donc du coup, là, je vais évaluer le périmètre de marche et les difficultés sur l'environnement. Si c'est un trottoir qui est plat, s'il y a des franchissements de trottoir, s'il y a un banc à disposition. Donc oui là je vais voir en fait en arrivant si le patient est donc en capacité ou pas de faire ce type d'effort supplémentaire en dehors de ses habitudes quoi.

BJM : Au cours de votre formation initiale, le niveau d'intensité des séances des patients post AVC a-t-il été abordé ?
MK10
Oui, on avait des tests, mais je les utilise pas vraiment. Aujourd'hui, après c'étaient plus des tests d'équilibre et des tests à l'effort en soit. Non, c'était plus utilisé avec les APA.

BJM : Vous a-t-on encouragé à la mettre en œuvre ?
MK10
Oh Ben, de toute façon, on avait une série de de bilans à faire, donc oui, ça devait faire partie de notre protocole, en fait. Mais c'était imposé par le centre, c'étaient des prises en charge qui étaient très, protocolaire, qu'on n'a pas en libéral.

BJM : Vous a-t-on enseigner de quelle façon la mettre en œuvre ?
MK10
Non à mis à part dans certaines formations, oui. Et ces formations étaient encore assez spécifiques aussi. On les a mises peut-être en place par la suite. Mais voilà, non. En tout cas ce n'est pas dans mon quotidien actuellement en tout cas.

BJM : Est-ce que les patients ont des freins à faire des exercices plus intenses ?
MK10

Euh bah ça dépend encore une fois à de qui on parle. Si c'est un patient jeune qui a très envie de récupérer très vite, oui, non, il n'a pas de frein. Lui, on peut y aller. Et puis si ce sont des patients plus âgés, un peu dépressif, oui, à chaque fois, il y a des freins parce qu'ils ne sont pas motivés.

BJM : Les patients vous font-ils ressentir des peurs, des appréhensions, un manque de motivation à l'idée de réaliser des exercices d'un certain niveau d'intensité ?
MK11
Oui
BJM : Et vous, avez-vous des freins à faire faire de l'exercice plus intense à ces patients ?
MK10
Bah je vais y aller progressivement, mais encore une fois, tout dépend de l'objectif du patient. Si l'objectif c'est de marcher, de se déplacer sans canne, on va y aller progressivement. On va d'abord travailler de l'assurance. Et on y va, on accompagne le patient. Si son objectif, c'est de pouvoir se lever sans accoudoir, Eh bien ça va être un gros effort, on va le faire progressivement et voilà. Mais c'est en accord avec le patient, c'est une question de motivation.

BJM : La sévérité du handicap, pourrait-elle être un frein ?
MK10
La sévérité ? Ah oui, bien sûr.

BJM : Et les troubles cognitifs ?
MK10
Et également. Bien sûr.
BJM : Et concernant la fatigue, la douleur et l'anxiété. Est-ce que cela peut constituer aussi des freins ?
MK10
Ben oui.

BJM : Les conséquences que cela pourrait engendrer au niveau cardiaque ou autres pourrait-elle être un frein ?
MK10
Euh bah je dirais que oui, on ne va pas pousser le patient si on sait qu'il a des problèmes cardiaques. Enfin, si c'est ponctuel, on ne va pas le mettre en danger mais si c'est connu, on va avoir nos limites qu'on ne va pas peut-être dépasser quoi ou on va y aller encore une fois prudemment.

BJM : Il est recommandé de faire 20 Min d'activité physique d'intensité modérée en séance de rééducation pour ses patients. Pensez-vous que l'on doive l'intégrer à la pratique de routine, au traitement kinésithérapique ? Ou bien Pensez-vous que cela devrait faire l'objet de séances complémentaires, comme des séances d'activités physiques adaptées ?

MK10 : Je pense que tout dépend encore une fois du niveau du handicap du patient. Mais si déjà pour lui se lever le matin, être autonome, prendre enfin faire sa toilette, l'habillage, être autonome sur son petit déjeuner, de pouvoir se déplacer tout seul et avoir un périmètre de marche suffisant pour aller, pour avoir un objectif précis. Ça, ça peut déjà correspondre à minimum 20 Min par jour, Hein. Au-delà du travail sur un vélo ou du travail spécifique en kiné, ce sont déjà des

dépenses d'énergie importantes pour ce type de patient. Donc si déjà il fait tout ça tout seul lui-même c'est déjà bien. Tous les jours et le supplément, c'est ce qui va travailler en kiné et en APA.

BJM : Prescrivez-vous des exercices que le patient doit faire chez lui ?
MK10 : Oui. Déjà des auto-mobilisations et donc un entretien articulaire et musculaire avec des exercices de temples. Ça peut être fait allonger, ça peut être fait assis en bord de lit, ça peut être fait sur une chaise face à une table, oui.

BJM : À quelle fréquence ? Et à quelle durée ?
MK10
Bah disons que ça peut être fait au moins une fois par jour, voire 2 peut-être 10 Min 1/4 d'heure Grand Max.

BJM : Et quelle intensité ?
MK10
Bah ça dépend du nombre de répétitions ou ça dépend du type d'effort. C'est variable mais une intensité modérée en tout cas.

BJM : Bon, c'est à la fin de l'entretien, je vous remercie pour ce temps d'échange, souhaitez-vous ajouter une dernière remarque concernant le thème que nous avons évoqué aujourd'hui ?

MK10
Non. Bah visiblement ça, l'air d'être assez précis. Le thème est quand même sur l'intensité de l'effort et tout est ciblé là-dessus. Mais c'est vrai que c'est très variable d'un patient à l'autre. Donc pour faire une généralité, il vous faut beaucoup de cas quoi évidemment. Mais si moi je dois faire un constat sur mes patients, encore une fois, les patients les plus âgés et les plus chroniques sont les plus difficiles à motiver. Dans la progression parce qu'il se sent déjà au bout de leur limite quoi. Et les plus jeunes avec plus de capacités motrices, bien évidemment. Sont plus faciles à emmener dans une intensité à l'effort.

BJM : Donc avez-vous des questions à me poser ?
MK10
Ben non, bon courage.
BJM : Ouais, je vais éteindre l'appareil d'enregistrement.

Retranscription Entretien 11 (Enquête11, MK11)
Entretien réalisé le 14 avril 2023
Durée 28 minutes 47 secondes
Présentation des interlocuteurs : Bénédicte JEAN MICHEL(BJM), Masseur-Kinésithérapeute (MK11)
Fichier audio
audio1330109423 1.m4a

Transcription

BJM

Pour débuter cet entretien, puis-je, s'il vous plaît, vous demander de vous présenter de façon assez brève, votre âge, la date de votre diplôme, votre expérience professionnelle et vos formations complémentaires.

MK11

D'accord, alors du coup je m'appelle X, j'ai 34 ans, je travaille au Palais-Sur-Vienne à côté de Limoges. Je suis installée en libéral depuis 5 ans maintenant, je suis diplômée depuis 2012 et avant j'ai fait des remplacements et plusieurs assistanats avant de m'installer. Au niveau des formations complémentaires, ce sont plutôt des formations du coup assez courtes : soit la formation au niveau des ligaments croisés du genou, soit la kiné respiratoire pour les nourrissons. Ce sont des formations, en ligne et régulières tous les ans pour continuer la reconnaissance de mon diplôme.

BJM

Sur cent patients. Combien avez-vous de patients post AVC dans votre patientèle ?

MK11

Sur 100, je dois en avoir 5.

BJM : Combien de fois les voyez-vous par semaine et combien de temps dure la séance ?

MK1

Alors 2 fois par semaine chacun et la séance dure à peu près 3/4 d'heure.

BJM : Est-ce un groupe ou en individuel ?

MK11

En individuel.

BJM : Y-a-t-il un nombre limité de séances ou bien les voyez-vous en continu ?

MK11

Alors je les vois en continue de manière chronique, ouais toute l'année.

BJM : Donc, connaissez-vous des recommandations en termes d'intensité d'exercice pour cette population ou pas du tout ?

MK11

Non, pas spécialement.

BJM : Donc, à votre avis, la séance que nous avons observée avec votre patient, avec Mme X était intense ou pas du tout ?

MK11

Il n'était pas intense. Parce qu'en fait, après je m'adapte à la demande du patient.

MK11

Oui, ce que je veux dire, est-ce qu'on parle des 2 patients qu'on a été vus quand ils sont venus ? ou oui, alors sur les 2 patients déjà aujourd'hui que qui avaient été vus, y en a une qui a clairement abandonné au niveau de la Kiné quoi, qui ne progressait plus et qui avait plus de plus d'envie. Ça a duré des mois ou elle ou a raté une séance, alors raté deux, c'était de la faute de l'ambulancier, c'était sa faute à elle. Elle était complètement démotivée et j'avais essayé de faire une pause pendant quelques semaines en lui disant « vous revenez mais vous revenez, on n'arrête plus et on continue pour au moins maintenir tout ce qu'on a gagné ». Aujourd'hui, on a tout arrêté parce qu'elle n'avait plus envie vu qu'elle ne progressait pas. Elle espérait récupérer beaucoup plus. Sur les 2 patients

qui avaient été vus à l'étude, il n'y en a qu'un que je continue à voir. Voilà après juste pour vous préciser.

BJM : Était-elle représentative de ce que vous faites habituellement avec ces patients ?
MK11
Alors ça représentait le travail qu'elle fait dans mes séances, mais clairement. Je pense que l'intensité, si on reste sur du cardiaque, n'était pas intense quoi. Le souci que j'ai avec mes 2 patients, un petit peu moins, le monsieur que la dame, c'est qu'à la maison à côté ils ne font pas grand-chose même rien. Donc en fait, ce que je fais mais en séance ne peut pas suffire. Ils ont plutôt un objectif passif quand ils viennent en séance : c'est moi qui travaille et qui travaille les rétractions au niveau de la mobilité plutôt que de l'actif. J'arrive à faire faire un peu de vélo, un peu d'exercice, mais, je pourrais faire beaucoup plus s'ils étaient demandeurs.

BJM : Selon vous, quels sont les freins ou obstacles en termes d'équipement à la mise en œuvre d'une séance avec un certain niveau d'intensité ?
MK111
On peut avoir le matériel, au cabinet, j'ai 2 vélos. Normalement avec 2 vélos, si on veut faire de l'intensité, on est capable de faire de faire quelque chose sur 1/4 d'heure, 20 min avec des programmes où ils peuvent se mettre au niveau cardio, augmenter un petit peu le cardio et se mettre en un effort plus élevé. Mais, ce n'est pas forcément leur souhait.

BJM : En termes de matériel, avez-vous besoin de matériel spécifique ou atteindre un certain niveau d'intensité ?

MK11
Alors si après l'idéal est ce que ce serait d'être équipé de tous les matériels qui peuvent capter au niveau fréquence cardiaque, moi les 2 vélos que j'ai, ils captent la fréquence cardiaque dans la paume des mains à chaque fois c'est vraiment en positionnant et je ne trouve pas ça fiable du tout. On voit que la fréquence varie quand on fait un effort, mais on n'a pas vraiment de, on va dire de feedback de retour qui nous dit qu'ils sont trop en intensité ou alors qu'ils nous montrent qu'ils sont une intensité. Il faudrait du matériel qui soit capable de plus, bah plus mesurer leur effort au final.

BJM : Non ? Quel matériel pourriez-vous besoin ?
MK11
Bah tout ce qui va être plutôt au niveau des soit une montre cardio ou les bracelets qui peuvent se mettre au niveau de la poitrine et qui auraient un retour. Je ne sais pas par quel système. Sur écran, sur une tablette, sur les appareils que j'ai, que je ne peux pas mettre en place aujourd'hui quoi !

BJM : En ce qui concerne l'intensité de vos séances, le manque d'espace est-il une limite ?

MK111
Le manque d'espace que vous m'avez demandé. Oui, alors aussi oui, moi j'ai là où je suis dans mon cabinet, j'ai un petit moi, j'appelle ça un petit gymnase en fait, où j'ai des barres parallèles où ils peuvent marcher. Après, c'est vrai que si on avait plus de place. Sûrement que on pourrait marcher un peu plus et de manière plus intense si on avait plus d'espace. Oui, ça c'est sûr.

BJM : Donc, quel espace vous faudrait-il ?

MK11
Quel espace ? Ben voyez de 2 fois plus grand que ce que j'ai aujourd'hui, de voilà au moins 60, 70 m². Pour pouvoir faire des allers-retours ou des couloirs, des voilà.

BJM : Donc le temps dont vous disposez en séance vous semble-t-il suffisant ?
MK11
Alors ce n'est jamais suffisant. Enfin, par rapport à des patients comme ça, faudrait qu'ils fassent plus après moi, j'essaie de passer suffisamment de temps avec eux après ce qu'il faut, ce que fait plus le Monsieur que la dame. Et je pense que c'est ce qu'il faut après quand je propose quelque chose, le vélo, je ne peux pas rester à côté d'eux quoi. Donc en fait il faut qu'il soit motivé et que pendant 1/4 d'heure, ils se donnent sur le vélo. Alors que des fois, ce n'était pas toujours le cas, ils ont tendance des fois à s'arrêter à attendre. Ils ont besoin de beaucoup de surveillance des fois.

BJM : Donc combien de temps vous faudrait-il ?
MK11
Bah après déjà là, en en 3/4 d'heure 1h ils ont le temps normalement de travailler suffisamment 2 fois par semaine pour moi. Enfin, on peut toujours faire plus, après moi, ce que j'aimerais qu'il fasse plus et surtout ce que je leur demande, c'est de faire à la maison quoi, faut sortir, marcher dehors, il faut monter ses escaliers, il faut faire des exercices à côté et ça ne peut pas, ça ne peut jamais suffire sur ces rééducations-là, et sur beaucoup bien sûr que de simplement ce qu'on fait en séance de kiné quoi !

BJM : Donc vous prescrivez des exercices faire à la maison, à vos patients ?
MK11
Oui ouais, ils en ont, surtout le Monsieur. La Dame, elle n'est pas trop réceptive donc j'ai eu beau en parler je pense qu'elle en fait pas. Après le Monsieur, il a tout à la maison, il s'est équipé avec des ballons, des élastiques pour faire travailler son bras. Des legos aussi pour tout ce qui est motricité fine. Et après lui, le défaut c'est peut-être voilà vu qu'il a des un peu des pertes d'équilibre, il n'arrive pas forcément à marcher tout seul dehors dans la rue donc. Tant que sa femme n'est pas là, il est un peu limité quoi. Mais sinon, il essaie de y aller régulièrement, donc ça c'est bien.

BJM : Donc à quelle fréquence ils font leurs exercices à la maison ?
MK11
Alors moi j'aime bien que ce soit fait, ils en connaissent ou il faut faire des exercices de base, de de mobilité au niveau des jambes et de la marche que je conseille tous les jours voire 2 fois par jour. Je pense que le monsieur les fait, la dame, je ne pense pas. Et après la marche, pareil, c'est quotidien. Quoi ! Après, ils ont toujours des bonnes raisons, malheureusement des excuses. On va dire parce qu'il ne fait pas beau parce qu'il fait pas ci. Voilà ça, je ne peux pas l'empêcher.

BJM : Et en termes de durée, l'intensité.

MK11
De ce qu'ils font eux, chez eux. On avait vu que l'intensité de l'effort était pas du tout élevée quoi. C'était une intensité faible, moyenne, mais ouais plutôt faible que moyenne. Donc je pense qu'ils sont très sédentaires, oui. Que ce soit des gestes du quotidien dans la maison, cuisine, salle de bain, salle à manger, chambre, ça reste des déplacements très limités dans un espace.

BJM : Y a-t-il un type de patients auxquels vous faites faire de l'exercice intense ?
MK11
Mais après, c'est beaucoup, c'est ce que je vous disais. Déjà sur le retour du patient quoi, si lui va en faire, moi j'ai beaucoup d'idées d'exercices à lui donner et lui faire faire. Après, c'est vraiment la motivation. Donc en fait je me base normalement plus on est jeune, plus on pourrait faire de d'exercice et mais ce n'est pas forcément vrai. Des fois, j'ai des patients qui sont arrivés là récemment ou qui ne faisaient pas partie de l'étude qui ont quasiment le double d'âge de la dame ou et qui ont été beaucoup plus actifs qu'en séance parce qu'ils se sont donné les moyens. Et je me base plutôt sur le bilan que j'ai fait au départ qui me montre s'il y a d'autres soucis de santé, s'il y a des antécédents. Et après, sur le l'envie et la motivation du patient quoi ! Ces capacités et son envie.

BJM : Accordez-vous la même importance à l'intensité de la séance en phase chronique qu'en phase subaiguë après AVC ?
MK11
Oui. Je n'ai pas forcément de différence. Je me bats plus à ce qu'il demande sur le moment, que l'intensité peut être la même en fait, en subaigu qu'en chronique suivant le comment eux sont moteurs de haut niveau des séances quoi.

BJM : Donc avez-vous besoin que le patient ait effectué une épreuve d'effort ou que le médecin vous ait donné l'autorisation d'intensifier l'effort ?
MK11
Alors oui bah si oui. Même avant de leur faire faire du vélo tout ça, je demande l'aval du médecin parce qu'on ne sait jamais. Parce qu'aujourd'hui on est en libéral donc on n'est pas forcément de lien avec le CHU, avec l'identité des autres structures qui accueille ses patients une fois qu'ils ont fait leur AVC. Et en fait des fois ils ont des antécédents, quand on leur pose la question, est-ce qu'il y a des soucis de santé ? Il n'y en a pas, n'y en a pas, mais au final ils ont un traitement pour le cœur. Ils ont d'autres choses. Donc en fait je demande toujours un avis ou du spécialiste, du neurologue au niveau du CHU ou du médecin traitant qui va me dire oui, on peut y aller ou attention il peut être fragile de ce côté-là ou sur telle ou telle chose. Ouais, je les surveille beaucoup au tout départ. Savoir s'il se sent bien, on en fait 5 Min au début. Après on fait 10 Min, on essaie d'augmenter l'intensité aussi. J'arrive pas dès le début, dès la première séance à la même intensité qu'au bout d'une dizaine de séances, forcément.

BJM : Alors faites-vous faire un test de poste maximal comme le test de la marge 6 Min ou le test de levée de chaise ou un autre test ?

MK11
Alors franchement, c'est ce qu'avait fait du coup à l'étude là vos collègues qui étaient venus, je ne fais pas faire les tests comme ça. J'ai un bilan qui reste un bilan, on va dire libéral, je n'ai pas un repère comme ça de ce test de 6 Min ou d'aller-retour, de chronométrer des choses comme ça ou après je vois, moi on va dire le déplacement quand on est dans le cabinet et qu'on travaille tous les. 2 je n'ai pas un test. Voilà, ça reste assez à mon observation à moi et sachant que je les vois, c'est le même kiné qui les suit toute l'année, je sais le niveau qu'ils ont au jour. Il n'y a rien de noir sur blanc quoi, pour être honnête, on n'utilise pas vraiment de test. J'ai un bilan que tout fait, mais je n'ai pas de test spécifique comme cela, quoi !

BJM : Évaluez-vous l'intensité des séances chez vos patients ?

MK11
Avec un outil ? Non, enfin, je l'évalue, moi de manière on va dire subjective. Je vois si en fait s'ils sont fatigués à la fin de la séance, on voit l'attitude qu'ils ont. Si pendant la séance, en fait on voit qu'il faut les freiner et qu'ils sont montés un petit peu trop haut ou en fait ils repartent, ils ne sont pas forcément fatigués quoi. C'est plus une appréciation subjective, on va dire, je ne l'évalue pas avec quelque chose de concret.

BJM : quelle matériel utilisez-vous augmentez l'intensité de vos séances est-ce que vous utilisez des ergomètres d'aspérule ou autres matériels ?
MK11
Pour calculer l'intensité.

BJM : Pour augmenter l'intensité ?
MK11
Pour l'augmenter, si ça. Voulait dire. Pour le moment, et après ce que j'augmente, j'augmente soit le nombre de répétitions dans les exercices que je demande, la répétition ou la durée. Et après, si c'est du vélo ou un autre appareil, ça peut être augmenté la résistance que je leur propose. Après, des fois, on fait varier les séances qui sont des fois moins intenses quand on fait plus le bras. Quand c'est les exercices plutôt avec des ballons, des élastiques pour travailler le renforcement et la mobilité du bras. Donc ça c'est toujours un peu moins intense forcément pour eux. Après, dès qu'on va travailler la marche et l'équilibre ou passer des obstacles, des échelles de rythme, tout ça. Là, ça devient beaucoup plus intense. Forcément, des cas du déplacement.

BJM : Lorsque vous prévoyez un exercice intense pour un patient, sur quels critères adaptez-vous ? L'intensité ?

MK11
Les critères, c'est surtout l'état on va dire, le ressenti et la condition physique du jour. Il y a des fois, ils arrivent, ils sont fatigués, ils ont d'autres soucis. On s'adapte, on va dire au ressenti du patient et à la capacité au jour le jour.

BJM : Donc comment ajustez-vous les séances en termes de durée, d'intensité et de fréquence ?
MK11
Alors j'ajuste, si on voit qu'aujourd'hui ça ne va pas forcément on fait assez peu d'exercice ou on en fait moins, on fait que le vélo, voilà. On adapte aux besoins et voilà et à la demande du patient sur le moment. Après des fois j'essaie moi de pousser un peu plus. Et quand c'est des séances qui se répètent plusieurs, on voit qu'on peut plus faire de vélo ou que y a des choses qui veulent plus faire là par contre j'essaie d'aller à l'inverse, d'être assez moteur et de et de leur dire ça fait longtemps qu'on a pas fait, il faut en faire quoi ?

BJM : A la suite des résultats préliminaires de l'étude Observationnels à laquelle vous avez participé, nous avons remarqué que l'intensité des séances différait d'un patient à l'autre, Quel regard portez-vous sur ce constat ?

MK11

L'intensité varie d'un patient à l'autre ? Que pour moi, ce Conseil est valable pour tous les patients, dans toutes les rééducations. On parle de l'AVC aujourd'hui, mais en fait, si on prend tous les patients différents qu'on a au cabinet il y en a qui, on va dire, veulent prendre un petit peu les choses en main et ont compris que ce qu'on faisait ne suffisait pas et d'autres qui arrivent à être beaucoup plus intenses et à se donner les moyens en séance ou c’est très patient-dépendant et pas que dans cette pathologie. Pour moi, ça peut s'étendre à notre travail au quotidien. Après, ça reflète quand même bien ce que moi je pensais. Voilà, je vois très bien dans les séances quand ça ne progresse pas et qu'on stagne, c'est aussi qu'à côté on ne fait pas. Donc en fait l’intensité ne peut pas augmenter si en fait on se voit que 2 fois par semaine, il y a tous les autres jours de la semaine. Au fur et à mesure, on ne peut pas avoir une intensité qui augmente énormément si derrière on n'arrive pas à entretenir et maintenir tout ce qu'on peut gagner sur une séance quoi !

BJM Donc, quelle importance accordez-vous à l'intensité des exercices pour ses patients ?
MK11
Pour moi, ça serait l'intensité importante. Après ce que je trouve qui manque surtout pour certains patients, c'est la régularité quoi ! Ce n’est pas forcément de faire un effort intense, c'est qu'il faut qu'il le fasse. C'est du quotidien. Voilà, c'est des maladies, ils ne vont pas s'en sortir. Il y a qui ont très peu de séquelles et d’autres qui ont beaucoup de séquelles. En fait, si aujourd'hui ils ne se prennent pas aux mains et ça ne devient pas du quotidien, ça faut que ce soit des exercices de tous les jours, même si ce n’est pas aussi intense qui peuvent le faire. Si c'est fait tous les jours, ce sera bénéfique alors que des fois... Pourquoi ils reviennent en séance ? Ils m'ont dit qu'ils avaient été marcher pendant 1h, mais par contre, 4 jours après, ils ne faisaient plus rien. Dans 4 jours. Ils étaient fatigués, ils étaient crevés. Donc pour moi ce n’est pas bénéfique, soit on y va tous les jours 1/4 d'heure et dans ce cas-là c'est super, on va s'entretenir et être qu'après on fera 20 Min, une demi-heure, des fois ils font trop d'un coup et après ils sont plus capables de faire.

BJM : Donc, lorsque vous augmentez l'intensité des séances, ce qu'elle, quel critère de surveillance utilisez-vous, est-ce que vous utilisez la fréquence cardiaque, la saturation ou la perception d’effort ?
MK11
Là, c'est plus la perception à l'effort. Au final, je n'utilise pas d'appareils qui capte leur fréquence cardiaque. Mais on se rend compte que les patients nous au quotidien, moi ceux que j'ai au cabinet ou à domicile, ils ne vont pas se mettre dans le rouge. Voilà, clairement, ils ne vont pas au-delà du ressenti. On va dire d'une intensité, d'un effort d'intensité moyenne quoi.

BJM : Donc, vous sentez-vous compétent en matière d'augmentation de l'intensité en séance de ces patients ?
MK11
Alors compétente, je ne suis pas sûre, on peut toujours faire plus, hein. Après le souci c'est qu’il faut qu’eux aient envie de le faire. Après, il faut avoir un peu plus de voilà comme on disait tout à l'heure de moyens, de temps. Peut-être de connaissances sur certaines choses aussi, peut-être qu'y a des choses que j'ignore et qui pourraient m'aider à les inciter un peu plus.

BJM : Au cours de votre formation initiale, le niveau d'intensité des séances des patients pour s'aviser a-t-il été abordés ?
MK11
Clairement non, je ne crois pas. Enfin ça ne m'a pas parlé. Après c'est beaucoup la rééducation des AVC en fait on n'a pas eu, on a des cours pour moi très... Alors ça fait du coup 10 ans que je suis diplômée. C'étaient plutôt des cours théoriques sur pourquoi on avait un AVC, comment faire un AVC, quelle est la pathologie après ça reste des pathologies où ça reste une maladie ou on peut tout faire quoi ! Il faut travailler le bras, la jambe, il faut travailler la coordination. Et voilà, la marche, le transfert, l'équilibre, la préhension fine. En fait, on a un panel d'exercices et de potentiel qui est énorme donc ce sont des patients en fait qui méritent beaucoup d'attention et beaucoup de soins. Et après on adapte en fait nos techniques qu'on connait et qu'on utilise à tout le monde à ce dont ils ont besoin. Voilà, c'est beaucoup une adaptation aux patients, mes séances ne sont pas les mêmes d'un patient à l'autre, surtout dans ces maladies-là.

BJM : Donc, avez-vous fait des formations complémentaires à ce sujet ?

MK11
Sur la AVC ? Non, enfin pas pour des adultes. J'ai fait une formation complémentaire en fait sur tous les troubles du neurodéveloppement, en fait des enfants, parce que j'ai des enfants aussi. Dans ce cas-là, avec des hémiplégies, des hémi parésies, et là, j'avais fait une formation en plus. Ouais. Pour connaître. On se rencontre que ça ne fonctionne pas de la même manière que c'est un peu différent quand on les prend très jeunes ou quand ça leur arrive et qui sont déjà adultes.

BJM : Est-ce que les patients ont des fins à faire ? Des exercices plus intenses ?

MK11
Ils ont des freins, ils ont parfois, ils n'ont pas envie quoi. Je crois clairement, ce n'est même pas dû la capacité parce que je leur ne demande pas si elle peut marcher, on n'a rien besoin quoi. Enfin, je n'ai pas besoin, ils ont leur technique. S'ils ont une canne ou un déambulateur ou autre chose pour marcher, ça aide là. À la maison après le frein, c'est clairement l'envie d'y aller. Parfois, c'est des personnes aussi un peu isolées ou qui vivent seuls donc quand il n'y a pas un entourage stable, ils ne sont pas simulés, donc le frein peut être aussi le fait d'avoir quelqu'un derrière de moteur au niveau familial. Après ce n'est pas un frein matériel en tout cas pour moi.

BJM : Donc les patients vont-ils ressentir des peurs, des appréhensions, un manque de motivation à l'idée de réaliser des exercices plus intenses ?
MK11
Ah oui, oui, ouais, clairement oui. Après la peur, c'est toujours compliqué parce qu'on ne peut jamais savoir comment ils vont récupérer. La seule chose qu'on sait, c'est que sur les patients qu'on a au stade chronique, quand on a dépassé un an, ouais, voire 6 mois, un an après, c'est compliqué de récupérer beaucoup. On récupère des choses mais pas une fonctionnalité comme il voudrait. Donc en fait quand des fois il manque quelque chose de moteur qui est assez important au quotidien et qui les handicaps, il lâche en voyant que ça n'avance pas quoi !

BJM : Et vous, avez-vous des freins à faire faire de l'exercice plus intense à ces patients ?
MK11

Alors moi des freins, c'est toujours ce qu'on disait tout à l'heure, hein, moi, sans avoir allumer le sang, je ne vais pas trop en intensité. Aujourd'hui, par peur qui se passe autre chose quand ils ont parce que là quasiment tous ceux que j'ai ont eu des problèmes cardiaques et même l'AVC est venu pendant une opération ou des choses comme ça, qui du cœur donc. Clairement, ce n'est pas quelque chose. Moi je les mettrai pas en difficulté là-dessus. Il y a pour moi, il y a un niveau si c'est des patients qui arrivaient à avoir déjà une activité à côté, qui était très bonne, là on pourrait faire plus aujourd'hui, s'ils ne font pas plus à côté et s'ils n'ont pas une intensité déjà moyenne en dehors de mes séances, je peux pas faire l'intensité élevée pendant mes séances quoi !

BJM : Donc les conséquences que cela pourrait engendrer au niveau cardiaque pourrait constituer un frein ?
MK11
Oui.

BJM : Donc est-ce que la sévérité du handicap ou leurs troubles cognitifs peuvent constituer un frein ?

MK11
Non, assez peu finalement. Moi, dans les patients que j'ai en ce moment, je n'ai pas forcément beaucoup de troubles cognitifs. C'est là où j'ai des freins, parfois, c'est quand c'est des plutôt un milieu social un peu plus défavorisé aussi. Peut-être voilà où il y a, ce qu'on disait tout à l'heure, un foyer et une famille pas forcément stable au niveau relationnel après. Je n'ai pas forcément de patients avec des troubles cognitifs, j'ai des patients qui ont des troubles de la parole, des aphasies, mais on arrive à communiquer, on arrive, voilà à se comprendre et à discuter donc. On passe outre, ce n'est pas ce n'est pas un frein, non ! Ils comprennent mes exercices. Et puis même si au début ils ne comprennent pas, on fait, on refait, on refait finalement avec la répétition, c'est des choses qu'ils ont acquis et qu'ils arrivent à faire très, très bien.

BJM : Et concernant la fatigue, la douleur et l'anxiété, des patients pourraient-ils constituer des fonds ?

MK11
Alors la douleur, je les trouve pas très douloureux moi finalement. C'est pas des patients qui me signifient beaucoup de douleur, c'est plutôt des patients qui vont être fatigués très vite, ça c'est sûr. La fatigue arrive beaucoup plus vite qu'un autre patient. Après, c'est ça qui est un frein quoi, quand ils sont fatigués, si y a autre chose qui ne va pas, ça va prendre le dessus. Voilà, on a du mal à dans les séances à être efficace. S'il y a un contexte où ils sont pas très bien.

BJM : Donc il est recommandé de faire 20 Min d'activité physique d'intensité modérée en séance de rééducation. Pensez-vous que l'on doive l'intégrer dans la pratique de routine du au traitement kinésithérapique ou bien Pensez-vous que cela devrait faire l'objet de séances complémentaires par exemple des séances d'activités physiques adaptées ?

MK11
Alors pour moi, ça peut être les 2 quoi. Enfin en fait, moi ce que je fais quand je vous dis je les garde 45 Min, c'est que déjà pendant 1/4 d'heure 20 Min je mobilise et je m'occupe de la jambe donc ça ce n'est pas du bras ou de la jambe. Et après ça fait à peu près ça, ça fait à peu près 20 Min

de mouvement d'efforts. Après le souci, ce sont des patients pour moi qui sont confrontés à des kinés qui ne veulent pas toujours les prendre en charge en Libéral, hein, qui clairement ont des fois des refus, donc ça c'est un peu dommage. Parce qu'on sait que ce sont des patients, une fois qu'on accepte qu'on aura longtemps très longtemps, quoi. Pendant des années et des années donc. Après, c'est des pour moi, ce sont des patients qui pourraient très bien faire nos 2 séances de kiné. Et puis à côté, faire de l'activité physique adaptée en plus hein, pour moi c'est très complémentaire quoi.

BJM : En résumé, qu'est-ce qu'une séance intense selon vous ?
MK11
Qu'est-ce qu'une séance intense pour une séance intense ? C'est quand le patient, on voit que déjà il est un peu plus, comment dire un peu plus marqué au niveau physique. Et qu'il se met au-delà de ces limites finalement, qu'il essaie de frôler ces limites au niveau équilibre, au niveau marche, au niveau physique. Bah plus, on a atteint ses limites, plus on la repousse quoi. Ce que on ce qu'on fait, je ne pense pas assez au niveau des séances en libéral et en moi en tout cas, au sein du cabinet quoi !

BJM : Donc, c'est la fin de l'entretien, je vous remercie pour ce temps d'échange, souhaitez-vous ajouter une dernière remarque concernant le thème que nous avons évoqué aujourd'hui ?
MK11
Non, pas spécialement, c'est.

BJM : Donc pas de question non plus
MK11
Non pas de questions non plus, non, non, peut-être, peut-être si. Il n’a pas trop été abordé, c'est que j'en ai maintenant que je vois à domicile aussi les patients AVC. Donc là c'est encore différent, c'est encore d'autres prises en charge parce qu’à domicile on a un peu moins de temps. Voilà là l'intensité encore, ça pourrait être aussi une étude en évolution de voir comment ces patients, quand ils vieillissent, parce que là, clairement c'est un monsieur, moi qui a vieilli et qui peut plus conduire, donc je vais chez lui ou un autre qui malheureusement a subi le confinement et qui est devenu plus sédentaire et qui peut plus se déplacer vers moi. Voilà, mais là, l'intensité est encore différente quoi. C'est encore autre chose de ce que nous on propose parce qu'on n'a pas de matériel, on est chez eux et de ce qu’eux peuvent faire à la maison quoi. Voilà juste. Pourrez voilà faire des ouvertures si voilà, si une autre étude est prévue.

BJM : D'accord, je vais éteindre là, justement.

Retranscription Entretien 12 (Enquête 12, MK12)
Entretien réalisé le 14 avril 2023
Durée 20 minutes 41 secondes

Présentation des interlocuteurs : Bénédicte JEAN MICHEL(BJM), Masseur-Kinésithérapeute (MK12)
Fichier audio
audio1340294270.m4a
<u>Transcription</u>

BJM : Donc pour débuter cet entretien, puis-je, s'il vous plaît, vous demander de vous présenter de façon assez brève : votre âge, la date d'obtention de votre diplôme, votre expérience professionnelle et vos formations ?
MK12
D'accord, alors moi je m'appelle X, je suis diplômée de 2017. J'ai 38 ans et j'ai alterné un mode d'exercice libéral et salarié. J'ai été notamment salariée dans un centre de rééducation où j'ai fait beaucoup de neurologie. Et ça fait 4 ans et demi que je suis revenue en libéral où j'ai fait d'autres spécialisations, notamment tout ce qui est pelvipérinologie, cancer du sein et développement neuromoteurs de l'enfant.

BJM : Donc, depuis combien de temps exercez-vous en libéral ?
MK12
Alors tout confondu, j'ai exercé en libéral pendant, ça fait 6 ans et demi que je fais du libéral sur toute ma carrière.

BJM : Combien de patients posent AVC suivez-vous actuellement.
MK12
Actuellement, j'en ai en. J'en ai 2, non 3 pardon.

BJM : Et ça, on présente combien en proportion sur votre patientèle ?
MK12
Allez, 3 à 5%. Vraiment pas grand-chose maintenant.

BJM : Combien de fois les voyez-vous par semaine et combien de temps dure la séance ?
MK12
J'en ai, j'ai plutôt en entretien et je les vois 2 fois par semaine pendant 1h chacun. Avec plusieurs patients et il y en a un qui est qui a fait son AVC l'été dernier, donc qui a moins d'un an et du coup je le vois 4 à 5 fois par semaine pendant 1h.

BJM :Donc c'est un groupe ou en individuel ?
MK12
En groupe.
BJM : Ce combien de séances les voyez-vous habituellement ?

MK12
Et Ben entre 2 et 4 fois par semaine.

BJM : Ce nombre de séances, limité dans le temps, voyez-vous ces patients en continu ?
MK12
Non, non, il n'y a aucune limitation en termes de nombre de séances. À l'heure actuelle, mais patients chroniques, je ne suis que leur seule ressource pour faire du sport entre guillemets, un entretien.

BJM : Les voyez-vous sur plusieurs mois ou sur plusieurs années ?
MK12
Oui, le plus ancien que j'ai, ça fait 3 ans.

BJM : Connaissez-vous des recommandations en termes d'intensité d'exercice pour cette population ou pas du tout ?
MK12
Non, pas plus que ça. J'essaie d'appliquer des recommandations qui, en fonction de leurs facteurs de risque correspondant aux recommandations de la population générale. S'ils n'ont pas de facteurs de risque supplémentaire, j'applique ceux de la population générale en termes d'intensité.

BJM : Quelle importance accordez-vous à l'intensité des exercices pour ces patients ?
MK12
Bah ça dépend du profil de mes patients. Il y a des patients qui en fonction de leur profil, ne peuvent pas. C'est vraiment aléatoire. Il y a certains patients que je peux pousser et il y en a autres ou ce n'est pas leur demande et ça colle pas leur projet. Donc ce n'est pas mon importance qui est la plus importante à mon à mon sens, c'est l'importance quand je leur présente leur programme de rééducation. Important, ce qu'ils ont eux, à une progressivité et une intensité spécifique. Il y en a qui peut qui, qui ne supportent pas ou psychologiquement ou physiquement parlant, du moins surtout, psychologiquement parlant.

BJM : À la suite des résultats préliminaires de l'étude Observationnelle à laquelle vous avez participé, nous avons remarqué que l'intensité de ces séances différaient d'un patient à l'autre. Quel regard portez-vous sur ce constat ?
MK12
Donc bah qui a des patients qu'on arrive à maintenir et à faire bouger et qu'il y a des patients qu'on n'arrive pas du tout à être adhérent à certaines choses après. Il y a un

aspect psychologique un peu un peu compliqué. Je le trouve dans par rapport à mes patients qui ont participé à l'étude et la limite cognitive en est une.

BJM : À part les limites cognitives, y a-t-il d'autres freins que vous rencontrez par rapport à ces patients pour faire de l'exercice plus intense ?
MK12
En fonction du handicap, ils ont besoin d'un entourage qui va participer activement et ce n'est pas toujours le cas. Et ou l'entourage n'est pas n'est pas là, n'est pas présent.

BJM : Nous avons récemment envoyé le bilan de la séance que nous avons observée avec votre patient, Monsieur le Maire, David était, elle, intense ou pas du tout ?
MK12
Moyennement intense

BJM : Pourquoi elle était moyennement intense ?
MK12
Parce que c'est un patient qui a des freins au niveau cognitif et au niveau émotionnel. Donc je ne peux souvent pas aller au-delà de ce frein-là.

BJM : Était-elle représentative de ce que vous faites habituellement pour cette population ?
MK12
Certaines non. Justement à cause des freins cognitifs, émotionnels et comportementaux.

BJM : Qu'est-ce qu'une séance intense selon vous ?
MK12
C'est fait et c'est une séance où le patient et Ben va être solliciter suffisamment et on va le voir plus ou moins essoufflé ou le rythme cardiaque va augmenter de façon significative.

BJM : Selon vous, quels sont les freins ou obstacles en termes d'équipement à la mise en œuvre d'une séance avec un certain niveau d'intensité ?
MK12
Au cabinet, on n'en a pas forcément. Moi, je ne considère pas qu'il en est au cabinet. On est plutôt bien équipé, on a la possibilité de le faire chez tout le monde.

BJM : Avez-vous besoin de matériel spécifique pour atteindre le niveau d'intensité ?
MK12
Non, non, non, puisqu'on en a suffisamment.

BJM : Alors, quelles sont les matériels que vous utilisiez pour atteindre ce niveau d'intensité ?
MK12
Alors ou les équivalents de Motorhead membre Sup membres chez à Quadriceps plus ou moins aussi le poids du corps des résistances externes. Tous les niveaux d'évolution moteur de l'enfant et du moins tous les nouveaux d'évolutions moteurs, donc en fonction du nombre de retournements à la vitesse. D'exécution on n'a pas forcément besoin d'avoir beaucoup plus de matériel, je rajoute des poids aussi éventuellement.

BJM : Selon vous, quel est le matériel nécessaire à l'atteinte d'un certain niveau d'intensité pendant les séances de rééducation de ses patients ?
MK12
Tout le matériel que j'ai déjà cité, que j'utilise déjà en fait.

BJM : En ce qui concerne l'intensité de vos séances, le manque d'espace est une limite ?
MK12
Non parce qu'on a un grand cabinet et on a beau couloir dans lesquels on les fait beaucoup marcher aussi.

BJM : Donc le temps dont vous disposez en séance vous semble-t-il suffisant ?
MK12
Oui, globalement oui.

BJM : Il y a-t-il un type de patients auxquels vous faites faire de l'exercice intense ?
MK12
Globalement à. La clinique. On teste petit à petit. Moi, je suis quelqu'un d'assez précautionneux, donc j'augmente mes charges de travail avec mes patients de façon progressive.

BJM : Donc vous vous utilisez quel type de test ?
MK12
Quel type de test ? Pas de test normé. Ça dépend des possibilités du patient. Euh, et des freins, éventuellement, quand ils mènent leurs courriers en termes cardiaques, quand j'ai un courrier spécifique du cardiologue ou du neurologue.

BJM : Accordez-vous la même importance à l'intensité de la séance en phase chronique, en phase subaiguë après AVC ?

MK12
Oui. Je considère qu’en phase sub chronique, subaigu, pardon, le patient va pouvoir pleinement maintenir ou acquérir certaines capacités en face chronique et se conserver.

BJM : Avez-vous besoin que le patient ait effectué une épreuve d'effort, que le médecin vous ait donné l'autorisation pour augmenter l'intensité de l'effort ?
MK12
Non en principe, c'est un accord avec le patient et quand j'augmente un effort, je reste bien en contrôle pour vérifier certains paramètres, garder l’essoufflement ne serait-ce qu'en clinique et éventuellement. Euh prendre certaines certains paramètres, que ce soit la saturation ou la fréquence cardiaque.

BJM : Donc voilà, fréquence cardiaque la saturation. Quels sont les autres critères de surveillance utilisez-vous lorsque vous augmentez l'intensité des séances ?
MK12
Il n’y en a pas d'autres, mise à part la surveillance clinique, vérifier s’il n’y a pas trop de tirage au niveau thoracique, au niveau respiratoire, changement de coloration, etc.

BJM : Vous n'utilisez pas la perception d’efforts du patient ou la vôtre ?
MK12
Oui, on pose la question. Ah si, si je lui pose la question. Je lui demande comment il perçoit l'effort, surtout si ça devient trop intense ou qu'il a une difficulté, ne serait-ce qu'à me répondre avec un ou 2 mots d'affiler, on arrête l'exercice. On fait une pause.

BJM : Donc le fait vous faire un test d'effort sous maximal comme le test de marque de 6 Min ou le teste de levée de chaise ou un autre test ?
MK12
Non, je fais aucun test normé ou apparenté.

BJM : Utilisez-vous des matériels augmenter l'intensité de vos séances comme des ergomètres, de des tapis roulants ou autre ?
MK12
Tapis roulant, vélo. Plus ou moins les résistances, donc les bras avec le motorhead ou les membres inférieurs aussi. Après, ce sont plutôt des appareils type appareil de musculation. S'ils peuvent s'en servir, chaise à quadriceps. Après, j'utilise aussi des dynamomètres.

BJM : Lorsque vous prévoyez un exercice intense pour un patient sur quel critère adaptez-vous l'intensité ?

MK12
J'augmente progressivement de séance en séance, le nombre de séries ou le temps de d'exercice.

BJM : Adaptez-vous l'intensité des exercices en fonction de l'état physique du patient ?
MK12
Oui. En fonction de sa présentation en début de séance, s'il est fatigué ou pas fatigué.

BJM : Comment ajustez-vous les séances en termes de durée et d'intensité et de fréquence ?
MK12
Alors, on j'augmente globalement plutôt le temps de pause et je raccourcis un petit peu certaines. Je diminue d'une ou 2 séries l'exercice en question. En accord avec le patient.

BJM : Vous sentez-vous compétent en matière d'augmentation de l'intensité de séance de ces patients ?
MK12
Oui, je me suis. Jamais posé la question faut que j'irais plutôt oui. C'est plutôt intuitif.

BJM : Au cours de votre formation initiale, le niveau d'intensité des séances des patients post AVC a-t-il été abordé ?
MK12
En formation initiale, c'était y a longtemps, hein ! Oh là, très peu parce qu'on n'était pas sur ce mode de fonctionnement là.

BJM : Donc, avez-vous fait des formations complémentaires à ce sujet ?
MK12
Oui. J'ai fait une réévaluation. J'ai refait une formation sur la prise en charge de l'AVC en 2016.

BJM : Avez-vous des freins à faire, de l'exercice plus intense à ses patients ?
MK12
Mise à part eux, c'est le seul frein, moi en tant que praticien, non !

BJM : Est-ce qu'il vous faut ressentir des peurs, des appréhensions, à un manque de motivation, à l'idée de réaliser des exercices d'un certain niveau d'intensité ?
MK12
Certains, souvent avec des troubles cognitifs justement.

BJM : A part les troubles cognitifs, est-ce que la C débité du handicap pour la motivation ou bien encore la fatigue pourrait constituer un frein ?
MK12
La fatigue, non, pas plus que ça parce que j'ai l'impression qu'ils se donnent quand même et qu'ils font leur maximum. Cause, conséquences, corrélation, je ne sais pas, une similarité entre les troubles cognitifs et la motivation en général. Ça va de pair. Et avec l'inhibition des mouvements chez l'hémiplégique droit plus que le gauche. Avec un manque, je ne trouve pas mes mots, avec un défaut de prise d'initiative souvent.

BJM : Est-ce que la douleur, l'anxiété ou elle constitue un frein également ?
MK12
Oui
BJM : Les conséquences que cela pourrait engendrer au niveau cardiaque ou autre pourraient-elles être un frein ?
MK12
Non, sauf contre-indication formelle du médecin et s'en est pas un. Pas pour moi.

BJM : Vous disiez tout à l'heure que. La douleur, l'anxiété pourrait constituer un frein. Que vous voulez-dire par là ?
MK12
La douleur et l'anxiété, ça peut constituer un frein parce qu'en fonction de la kinésie phobie que présente le patient ou des peurs engendrées par le mouvement. C'est plutôt ça le plus gros frein qu'autre chose.

BJM : Il est recommandé de faire 20 Min d'activité physique, d'intensité modérée en séance de rééducation pour ses patients. Pensez-vous que l'on doive l'intégrer à la pratique de routine, au traitement qui décidé rapide ou bien Pensez-vous que cela devait faire l'objet de séances complémentaires, comme des séances d'activités physiques adaptées ?
MK12
En phase chronique oui, qui y ait des séances en plus. Tant que l'état n'est pas consolidé, ça devrait rester en séance pour moi.

BJM : Prescrivez-vous des exercices que le patient doit faire chez lui ?
MK12
Auto, mobilisation, étirement.

BJM : À quelle fréquence et à quelle durée ?

MK12
Tous les jours et à la demande du patient en fonction du bien-être heureux senti et du besoin du moment.

BJM : Et à quelle intensité ?
MK12
Alors, ça, c'est une intensité faible vu que c'est que des exercices d'entretien.

BJM :D'accord, c'est la fin de l'entretien, je vous remercie pour ton échange de devoir ajouter une dernière remarque concernant le terme évoqué aujourd'hui ? Ou avez-vous des questions ?
MK12
Pas de question.
BJM : D'accord, je vais éteindre la parler d'enregistrement.
